# FBXW2 suppresses breast tumorigenesis by targeting AKT-Moesin-SKP2 axis

**DOI:** 10.1038/s41419-023-06127-x

**Published:** 2023-09-22

**Authors:** Ganesh Kumar Barik, Osheen Sahay, Anindya Mukhopadhyay, Rajesh Kumar Manne, Sehbanul Islam, Anup Roy, Somsubhra Nath, Manas Kumar Santra

**Affiliations:** 1 Cancer Biology Division, National Centre for Cell Science, Ganeshkhind Road, Pune, Maharashtra 411007 India; 2 Department of Biotechnology, Savitribai Phule Pune University, Ganeshkhind Road, Pune, Maharashtra 411007 India; 3 Saroj Gupta Cancer Centre and Research Institute, Kolkata, West Bengal 700063 India; 4 Department of Pathology, Nil Ratan Sircar Medical College and Hospital, Kolkata, West Bengal 700014 India; 5 Institute of Health Sciences, Presidency University, New Town, Kolkata, West Bengal 700156 India

**Keywords:** Breast cancer, Tumour-suppressor proteins, Oncogenes, Ubiquitylation, Ubiquitin ligases

## Abstract

Oncogene Moesin plays critical role in initiation, progression, and metastasis of multiple cancers. It exerts oncogenic activity due to its high-level expression as well as posttranslational modification in cancer. However, factors responsible for its high-level expression remain elusive. In this study, we identified positive as well as negative regulators of Moesin. Our study reveals that Moesin is a cellular target of F-box protein FBXW2. We showed that FBXW2 suppresses breast cancer progression through directing proteasomal degradation of Moesin. In contrast, AKT kinase plays an important role in oncogenic function of Moesin by protecting it from FBXW2-mediated proteasomal degradation. Mechanistically, AKT phosphorylates Moesin at Thr-558 and thereby prevents its degradation by FBXW2 via weakening the association between FBXW2 and Moesin. Further, accumulated Moesin prevents FBXW2-mediated degradation of oncogene SKP2, showing that Moesin functions as an upstream regulator of oncogene SKP2. In turn, SKP2 stabilizes Moesin by directing its non-degradable form of polyubiquitination and therefore AKT-Moesin-SKP2 oncogenic axis plays crucial role in breast cancer progression. Collectively, our study reveals that FBXW2 functions as a tumor suppressor in breast cancer by restricting AKT-Moesin-SKP2 axis. Thus, AKT-Moesin-SKP2 axis may be explored for the development of therapeutics for cancer treatment.

## Introduction

According to the global cancer statistics by GLOBOCAN 2020, female breast cancer has crossed lung cancer and become the most frequently diagnosed cancer. It remains the fifth leading cause of cancer-associated deaths worldwide [1]. Further, breast cancer remains the first rank cancer among women in terms of both the rate of incidence and rate of mortality [1]. Importantly, due to in-depth research, there are an increasing number of drugs approved by the United States Food and Drug Administration (FDA) to treat breast cancer patients. Though the breast cancer patient’s survival is better than other cancers, none of the drugs show 100% recovery from this deadly disease [2]. Therefore, it is of utmost importance to better understand the pathogenesis of breast cancer and identify the critical molecular players driving this disease, which will ultimately help us devise a better-targeted therapy to treat breast cancer.

Membrane-organizing extension spike protein (Moesin), a member of the ERM (Ezrin-Radixin-Moesin) family, is a membrane-cytoskeletal linker protein that plays pivotal roles in diverse physiological processes by linking the cytoplasmic actin filament with various plasma membrane-associated proteins [3–5]. Deregulation of Moesin leads to several pathological conditions, including cancer [5, 6]. A plethora of reports suggests that Moesin is overexpressed or aberrantly activated in diverse cancer types, including breast cancer, which is significantly associated with poor survival of cancer patients [5, 6]. AKT is a critical oncogenic kinase which is frequently activated in breast cancer [7]. A study reported that Moesin activates PI3K/AKT signaling pathway in glioblastoma [8]. Further, Moesin functions as a critical oncogene in breast cancer to drive growth, proliferation, invasion, and metastasis [6]. However, the posttranslational regulation of Moesin remains elusive.

Ubiquitination, one of the most studied posttranslational modifications (PTMs) in cell biology, plays a vital role in almost every cellular process. Ubiquitination refers to adding a 76 amino acid-long ubiquitin (Ub) molecule to the target protein. Ubiquitination is an ATP-dependent enzymatic process carried out sequentially by three classes of enzymes—Ub activating enzyme (E1), Ub conjugating enzyme (E2), and Ub ligase enzyme (E3) [9]. E3 ubiquitin ligases recognize the substrates those need to be ubiquitinated. There are almost 700 E3 ligases found in humans, and SKP1-Cullin1-F-box (SCF) E3 ligases are the most studied ones [10]. SCF E3 ubiquitin ligases consist of Cullin1 (the scaffold), SKP1 (the adaptor), and F-box (the variable component). F-box proteins are the substrate specifying subunits in SCF E3 ligases. There are around 70 F-box proteins encoded in the human genome [11]. Studies in the last two decades have shown that F-box proteins have essential roles in cancer progression; some act as oncogenes, some as tumor suppressors, while others act in a context-dependent manner [11–13]. However, the roles of many F-box proteins are either unknown or poorly studied.

In this study, we show that F-box and WD repeat-containing protein 2 (FBXW2) functions as a tumor suppressor in breast cancer. Through mass spectrometry analysis and other biochemical studies, we discovered Moesin as a direct target of FBXW2. Our in vitro and in vivo studies reveal that FBXW2 prevents breast cancer progression by promoting Lys-48-linked polyubiquitination to direct proteasomal degradation of Moesin through the canonical SCF complex. Further, FBXW2 is under expressed whereas Moesin is highly upregulated in breast cancer cell lines and patient samples and their converse correlation is closely associated with poor patient prognosis. We also identified an oncogenic AKT-Moesin-SKP2 axis which promotes breast cancer progression and FBXW2 restricts this axis to prevent cancer progression. Thus, this study demonstrates that AKT-Moesin-SKP2 axis can be explored to develop therapeutics for the betterment of cancer intervention.

## Results

### Moesin is a direct target of FBXW2 and they are conversely correlated in breast cancer

**Fig. 1:**
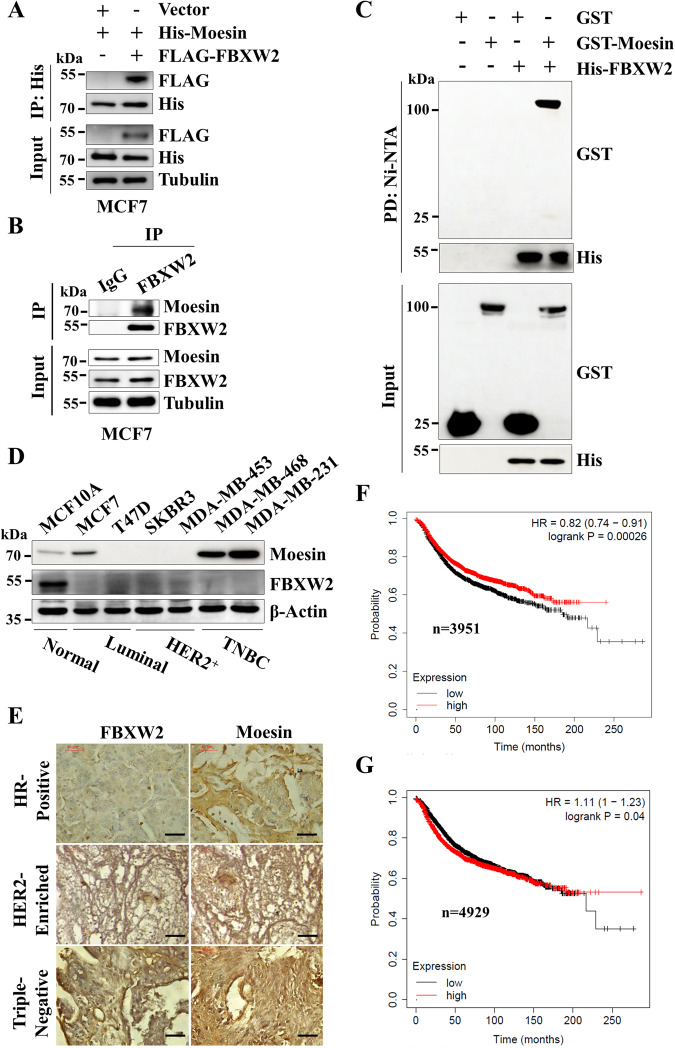
Moesin is a direct target of FBXW2 and they are conversely correlated in breast cancer. **A** MCF7 cells were transfected with indicated plasmids for 36 h followed by MG132 treatment for 6 h. Cells were harvested, whole-cell lysates were immunoprecipitated with anti-His antibody and then immunoprecipitates and input lysates were immunoblotted for the indicated proteins. **B** MCF7 cells were harvested and whole-cell lysates were immunoprecipitated with either anti-IgG or anti-FBXW2 antibody. Then, immunoprecipitates and input protein extracts were immunoblotted for the indicated proteins. **C** Recombinant purified GST and GST-Moesin proteins were incubated with Ni-NTA bead-bound FBXW2 at indicated combinations for 2 h at room temperature. Then, Ni-NTA beads were washed and pulled down proteins was eluted with SDS buffer, and elutes were immunoblotted for the indicated proteins. **D** Whole-cell lysates were prepared from normal breast epithelial cells (MCF10A) and different breast cancer cell lines as indicated, resolved in SDS-PAGE, and immunoblotted for the indicated proteins. **E** Representative immunohistochemistry (IHC) images showing FBXW2 and Moesin staining of tissue sections of three subtypes of breast cancer patients (HR positive, *n* = 7; HER2-enriched, *n* = 3 and TNBC, *n* = 3). Scale bars = 40 µM. Recurrence-free survival (RFS) of breast cancer patients having high or low mRNA expression of FBXW2 (**F**) and Moesin (**G**).

FBXW2 is a relatively less studied F-box protein. Recent studies showed that it could function as tumor suppressor or oncogene in multiple cancers [14–19]. However, its role in breast cancer progression is poorly understood. We speculated that FBXW2 may regulate several oncogenes to suppress breast cancer progression. Hence, FBXW2 was immunoprecipitated from MCF7 breast cancer cells. Immunoprecipitates of FBXW2 were resolved in SDS-PAGE, the unique bands present only in FBXW2 immunoprecipitates were excised, and subjected to mass spectrometry (LC-MS/MS) (Supplementary Fig. S1A). LC-MS/MS data analysis identified 84 interactomes of FBXW2 (Supplementary Table S1). While mining the literature for these interactomes, we discovered that several interactomes of FBXW2 act as oncogene in various cancers (Supplementary Table S2). We selected Moesin for further study because of its potential oncogenic activity in different cancers, including breast cancer [5, 6]. We first validated the interaction between FBXW2 and Moesin through co-immunoprecipitation (co-IP), followed by immunoblotting. The results revealed that FBXW2 interacts with Moesin at the exogenous level both in MCF7 and MDA-MB-231 cells (Fig. 1A and Supplementary Fig. S1B). In addition, FBXW2-Moesin interaction was also observed at the endogenous level in both MCF7 and MDA-MB-231 cells (Fig. 1B and Supplementary Fig. S1C, D). Finally, we examined their direct interaction using recombinant purified proteins His-FBXW2 and GST-Moesin. Immunoblotting results showed that FBXW2 directly interacts with Moesin under in vitro condition (Fig. 1C). Thus, Moesin is a direct target of FBXW2.

Next, we examined the expression levels of FBXW2 and Moesin in normal breast epithelial cell line and different subtypes of breast cancer cell lines (luminal, human epidermal growth factor receptor 2 positive (HER2^+^), and triple negative breast cancer (TNBC)). Immunoblotting results showed that Moesin expression is higher in luminal and highest in TNBC cells compared to normal cells (Fig. 1D and Supplementary Fig. S1E), suggesting that Moesin is highly expressed in 50% breast cancer cell lines tested. In contrast, FBXW2 is under expressed in all the breast cancer cell lines tested compared to the normal cell line indicating that ablation of FBXW2 might be responsible for accumulation of Moesin in breast cancer cells. We further examined their levels in a cohort of breast cancer patients with different subtypes (hormone receptor (HR) positive, HER2-enriched and TNBC) and observed similar results (Fig. 1E and Supplementary Fig. S1F). Further, Kaplan–Meier (KM) plot of The Cancer Genome Atlas (TCGA) datasets suggests that higher expression levels of FBXW2 are strongly associated with better recurrence-free survival (RFS) in breast cancer patients whereas higher expression levels of Moesin are strongly associated with poor patient RFS (Fig. 1F, G). To further evaluate the clinical relevance of these findings we have performed the KM survival analysis of GSE3494 dataset between high FBXW2/low Moesin and low FBXW2/high Moesin patients. We found that breast cancer patients with high FBXW2/low Moesin levels have significantly better RFS compared to those with low FBXW2/high Moesin (Supplementary Fig. S1G), suggesting that the expression levels of both FBXW2 and Moesin could be used as prognostic biomarkers in breast cancer. Taken together, FBXW2 directly interacts with Moesin and shows an inverse correlation with Moesin’s expression in breast cancer cell lines and patient samples.

### FBXW2 inhibits breast cancer progression by negatively regulating Moesin levels

**Fig. 2:**
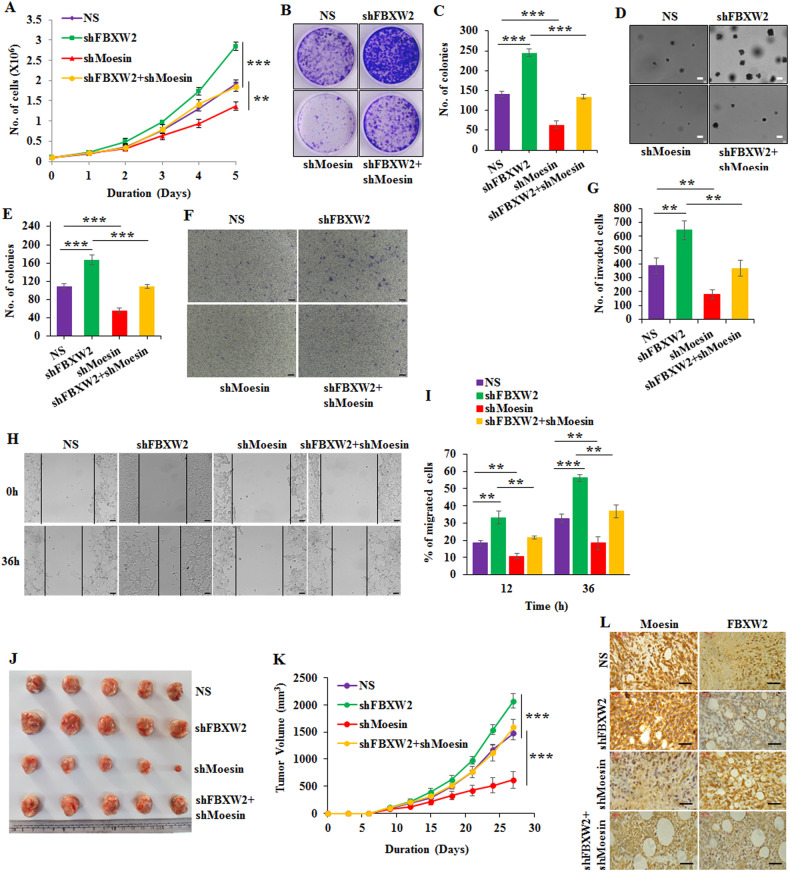
FBXW2 inhibits breast cancer progression through Moesin. **A** Trypan blue exclusion assay in MCF7 cells expressing either NS or shRNAs against Moesin or FBXW2 or both. **B** Long-term colony formation assay in MCF7 cells expressing either NS or shRNAs against Moesin or FBXW2 or both. **C** Quantification of colonies from (**B**). **D** Anchorage-independent soft agar assay in MCF7 cells expressing either NS or shRNAs against Moesin or FBXW2 or both. Scale bars = 8 µM. **E** Quantification of colonies from (**D**). **F** Invasion assay in MCF7 cells expressing either NS or shRNAs against Moesin or FBXW2 or both. Scale bars = 100 µM. **G** Quantification of invaded cells from (**F**). **H** Scratch wound healing assay in MCF7 cells expressing either NS or shRNAs against Moesin or FBXW2 or both. Scale bars = 100 µM. **I** Quantification of migrated cells from (**H**). **J** Xenograft assay showing tumors of 4T1 cells expressing either NS or shRNAs against Moesin or FBXW2 or both. Cells were orthotopically injected in mammary fat pad of NOD-SCID female mice (4–6 weeks old). **K** Xenograft assay shows the progression of tumor growth of 4T1 cells expressing either NS or shRNAs against Moesin or FBXW2 or both in NOD-SCID female mice. **L** Representative IHC staining of FBXW2 and Moesin in tumors derived from NOD-SCID mice. 4T1 cells expressing either NS or shRNAs against Moesin or FBXW2 or both were injected orthotopically. Scale bars = 40 µM.

Since Moesin is a well-established oncogene [5, 6] and FBXW2 is a putative tumor suppressor [14, 15, 17–19], we sought to determine whether FBXW2 regulates breast cancer progression through controlling the expression level of Moesin. To examine this possibility, we generated MCF7 cells with stable depletion of either FBXW2 or Moesin or both by using specific lentivirus short hairpin RNAs (shRNAs). We found that depletion of FBXW2 results in significant accumulation of Moesin (Supplementary Fig. S2A). We then performed a series of in vitro functional assays using these cells. Trypan blue cell count assay revealed that depletion of FBXW2 results in significant increased cell proliferation compared to the wild-type (NS) MCF7 and 4T1 (murine breast cancer cell line) cells (expressing scramble shRNA) (Fig. 2A and Supplementary Fig. S2B). In contrast, proliferation of Moesin-depleted cells is markedly decreased compared to NS cells (Fig. 2A and Supplementary Fig. S2B). Interestingly, depletion of Moesin in FBXW2 knockdown cells resumes the proliferation profile of NS cells. Further, we performed long-term colony formation assay to assess whether FBXW2 controls cancer cell proliferation through monitoring expression levels of Moesin. The results revealed that MCF7 and 4T1 cells form large number of colonies upon depletion of FBXW2, and conversely lesser number of colonies upon depletion of Moesin compared to NS cells; whereas cells depleted with both FBXW2 and Moesin form similar number of colonies to that of NS cells (Fig. 2B, C and Supplementary Fig. S2C, D). Taken together, these results show that FBXW2 controls the proliferation of breast cancer cells through controlling Moesin expression. Further, we examined the role of FBXW2 on breast cancer malignancy by monitoring anchorage-independent growth, migration, and invasion of MCF7 cells. Results revealed that anchorage-independent growth of MCF7 cells is increased following depletion of FBXW2. However, depletion of Moesin in FBXW2-depleted cells results in reduction of anchorage-independent growth (Fig. 2D, E). Similar results were also observed in invasion assay (Fig. 2F, G and Supplementary Fig. S2E, F) and wound healing cell migration assay (Fig. 2H, I and Supplementary Fig. S2G, H). So, these results showed that FBXW2 inhibits malignancy of breast cancer cells in vitro by negatively regulating Moesin. To further substantiate our observation, breast tumor growth in NOD-SCID mice was monitored following depletion of either gene or co-depletion of both Moesin and FBXW2. In agreement with the in vitro data, tumor growth of FBXW2-depleted cells is significantly increased compared to NS cells while co-depleted cells form tumor like NS cells (Fig. 2J, K and Supplementary Fig. S2I). Off note, the mice body weight remains unchanged (Supplementary Fig. S2J). Immunoblotting and IHC analysis of respective tumor samples showed efficient stabilization of Moesin upon depletion of FBXW2 (Fig. 2L and Supplementary Fig. S2K). Collectively, FBXW2 suppresses breast cancer progression both in vitro and in vivo by negatively regulating Moesin.

### FBXW2 negatively regulates Moesin levels through proteasome-mediated degradation

**Fig. 3:**
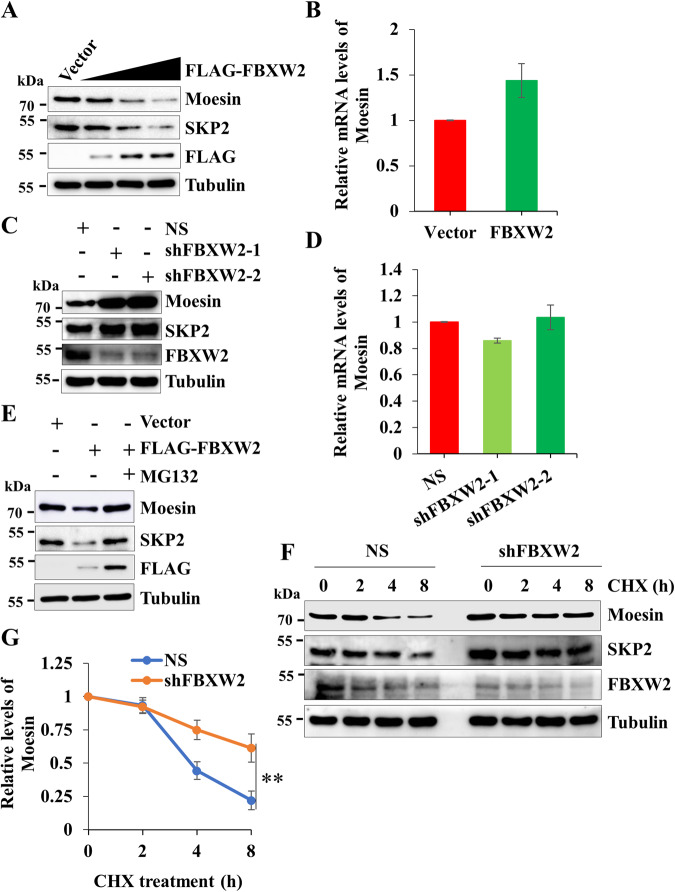
FBXW2 negatively regulates Moesin levels through directing proteasomal degradation. **A** Immunoblot showing the expression levels of Moesin, SKP2, FLAG-FBXW2 and Tubulin in MCF7 cells expressing either vector or different doses of FLAG-FBXW2. Tubulin was used as loading control throughout the study wherever mentioned. **B** Real-time qRT-PCR showing relative mRNA levels of Moesin in MCF7 cells expressing either vector or FLAG-FBXW2. β-Actin was taken as internal control. **C** Immunoblot showing the expression levels of Moesin, SKP2, FBXW2 and Tubulin in MCF7 cells expressing either scramble shRNA (NS) or FBXW2 specific two unrelated shRNAs. **D** Real-time qRT-PCR presenting relative mRNA levels of Moesin in MCF7 cells expressing either scramble shRNA (NS) or FBXW2 specific two independent shRNAs. mRNA level of β-Actin was taken as internal control. **E** MCF7 cells were transfected with either empty vector or FLAG-FBXW2 for 36 h. Then, cells were grown in the presence or absence of 5 μM of MG132 for additional 6 h as indicated. Whole-cell protein extracts were immunoblotted for the indicated proteins. **F** Control (NS) and FBXW2-depleted MCF7 cells were treated with 100 µg/ml of cycloheximide (CHX) for the indicated time periods and whole-cell lysates were immunoblotted for the indicated proteins. **G** Quantification of relative expression levels of Moesin in (**F**).

Next, we sought to explore how FBXW2 negatively regulates Moesin levels. Immunoblotting results showed that ectopic expression of FLAG-FBXW2 in increasing doses leads to a significant reduction in Moesin levels in both MCF7 and MDA-MB-231 cells (Fig. 3A and Supplementary Fig. S3A). Further, we noticed that FBXW2-mediated reduction of Moesin was not due to transcriptional repression since we did not find noticeable change at the mRNA level following ectopic expression of FBXW2 (Fig. 3B and Supplementary Fig. S3B). To further support the observation, expression levels of Moesin were examined following stable depletion of FBXW2 in MCF7 and MCF10A cells. We observed that depletion of FBXW2 leads to robust increased levels of Moesin in both the cell lines (Fig. 3C and Supplementary Fig. S3C). However, stable depletion of FBXW2 does not affect mRNA levels of Moesin (Fig. 3D). These data suggest that FBXW2 negatively regulates Moesin at the posttranscriptional levels. FBXW2 being a substrate-recognizing subunit of SCF E3 ligase and reported to target its substrates (SKP2, β-catenin, etc.) for degradation via the 26S proteasome pathway [14, 15, 17–19], we speculated that FBXW2 might negatively regulate Moesin via the proteasomal pathway. To assess this possibility, FBXW2 was ectopically expressed in MCF7 and MDA-MB-231 cells in the presence or absence of MG132 (a known pharmacological inhibitor of 26S proteasome). Overexpressed FBXW2 could not reduce the levels of Moesin in the presence of MG132, suggesting that FBXW2 facilitates the attenuation of Moesin through the proteasomal pathway (Fig. 3E and Supplementary Fig. S3D). To further support the proteasomal regulation of Moesin by FBXW2, we performed cycloheximide (CHX) pulse-chase assay. Immunoblotting results showed that depletion of FBXW2 significantly reduces the turnover of Moesin compared to the control (Fig. 3F, G and Supplementary Fig. S3E, F). Altogether, these observations indicate that FBXW2 facilitates the degradation of Moesin through the proteasomal pathway.

### FBXW2 facilitates proteasomal degradation of Moesin through Lys-48-linked polyubiquitination via the canonical SCF complex

**Fig. 4:**
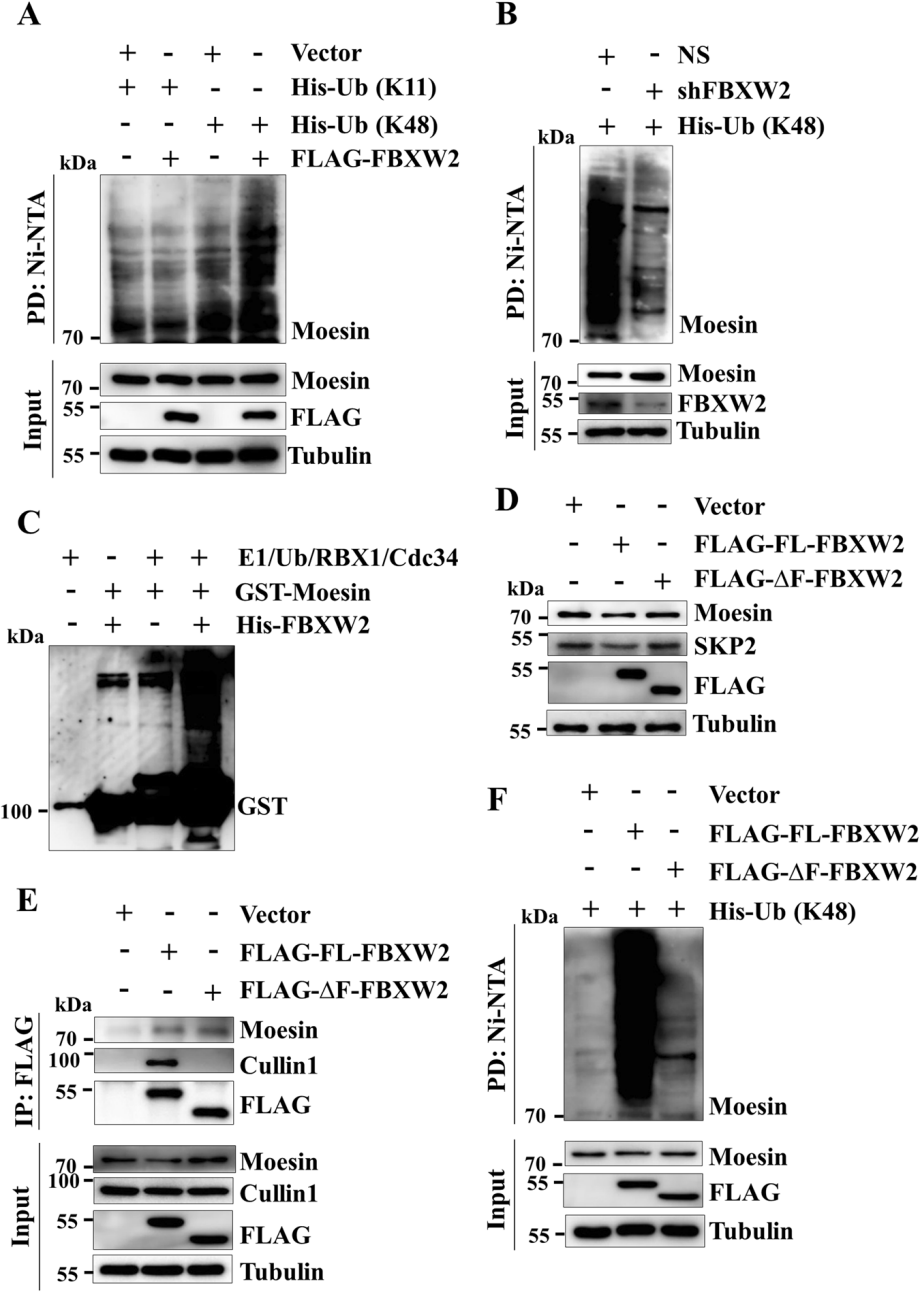
SCF^FBXW2^ facilitates proteasomal degradation of Moesin through K48-linked polyubiquitination. **A** MCF7 cells were transfected with indicated plasmids for 36 h. Transfected cells were then treated with 5 µM MG132 for 6 h. Ubiquitinated proteins were pull-down by using with Ni-NTA beads. Pulled down proteins and input protein extracts were immunoblotted for the indicated proteins. **B** NS and FBXW2 knockdown cells were transfected with His-Ub (K48) for 36 h. Transfected cells were then treated with 5 µM MG132 for 6 h and lysed under denaturing condition. Ubiquitinated proteins from whole-cell protein extracts were pulled down by using Ni-NTA beads. Pulled down proteins and input protein extracts were immunoblotted for the indicated proteins. **C** In vitro ubiquitination experiment was performed by using recombinant purified proteins. All the purified components and ATP were incubated with ubiquitination buffer (50 mM Tris, pH 8.0, 5 mM MgCl_2_, 1 mM β-mercaptoethanol, and 0.1% Tween 20) in indicated combination for 2 h at room temperature. Then, SDS sample buffer was added to stop the reaction and boiled for 5 min. The samples were then resolved in SDS-PAGE followed by western blotting with anti-GST antibody. **D** MCF7 cells were transfected with either vector, full length FBXW2 (FLAG-FL-FBXW2) or F-box motif-deleted FBXW2 mutant (FLAG-∆F-FBXW2) for 48 h. Whole-cell protein extracts were then immunoblotted for the indicated proteins. **E** MCF7 cells were transfected with either vector, full length FBXW2 (FLAG-FL-FBXW2) or F-box motif-deleted FBXW2 mutant (FLAG-∆F-FBXW2) for 36 h. Transfected cells were then treated with 5 µM MG132 for 6 h. Whole-cell protein extracts were immunoprecipitated with anti-FLAG antibody. Immunoprecipitates and input protein extracts were then immunoblotted for the indicated proteins. **F** MCF7 cells were transfected with the indicated plasmids for 36 h. Transfected cells were then treated with 5 µM MG132 for 6 h and lysed under denaturing condition. Ubiquitinated proteins from whole-cell protein extracts were pulled down by using Ni-NTA beads. Pulled proteins and input protein extracts were immunoblotted for the indicated proteins.

Generally, SCF E3 ubiquitin ligases direct polyubiquitination of their substrates to mark them for proteasomal degradation. It is reported that lysine-11 (K11) and lysine-48 (K48)-linked polyubiquitinated proteins undergo proteasomal degradation [20]. So, we asked whether FBXW2 promotes proteasomal degradation of Moesin via K11 or K48-linked polyubiquitination. To address this question, we examined polyubiquitination of Moesin using K11 (except K11, other 6 Lys residues of Ubiquitin are mutated to Arg) or K48 (except K48, other 6 Lys residues of Ubiquitin are mutated to Arg) ubiquitin mutants. Ubiquitin pull-down followed by immunoblotting results revealed that overexpressed FBXW2 significantly promotes K48-linked polyubiquitination of Moesin (Fig. 4A). Conversely, we found that K48-linked polyubiquitinated levels of Moesin are markedly declined in FBXW2-depleted cells (Fig. 4B), suggesting that FBXW2 facilitates K48-linked polyubiquitination of Moesin in vivo. To substantiate our observation, we performed in vitro polyubiquitination assay with the purified His-FBXW2 and GST-Moesin. The immunoblotting results showed that FBXW2 directly facilitates K48-linked polyubiquitination of Moesin (Fig. 4C). Thus, Moesin is a direct cellular target of FBXW2 for K48-linked polyubiquitination.

Preceding results demonstrated that FBXW2 directs proteasomal degradation of Moesin through promoting its K48-linked polyubiquitination. Previous studies showed that FBXW2 forms the SCF complex through its F-box domain to facilitate the proteasomal degradation of its substrates [14, 15, 18, 19]. We then speculated that FBXW2 might facilitate the degradation of Moesin through the SCF complex. To address this possibility, we generated F-box domain deleted FLAG-FBXW2 cDNA mutant (FLAG-∆F-FBXW2) and examined the levels of Moesin following overexpression of FLAG-∆F-FBXW2. Immunoblotting data showed that unlike full length FLAG-FBXW2 (FLAG-FL-FBXW2), FLAG-∆F-FBXW2 fails to attenuate the expression levels of Moesin (Fig. 4D). The incapability of FLAG-∆F-FBXW2 to degrade Moesin could be because of lack of interaction with Moesin or formation of SCF complex or both. To address these possibilities, we performed co-IP followed by immunoblotting. We found that FLAG-FL-FBXW2 could interact with Moesin and Cullin1 (the scaffold protein of the SCF complex). In contrast, FLAG-∆F-FBXW2 could interact only with Moesin but not with Cullin1 (Fig. 4E). In addition, we found that FLAG-∆F-FBXW2 fails to polyubiquitinate Moesin (Fig. 4F). Collectively, FBXW2 requires its F-box motif to form the canonical SCF complex to facilitate K48-linked polyubiquitination of Moesin and thereby promoting its degradation via the 26S proteasome.

### Moesin and SKP2 interact with and positively regulate expression of each other at the posttranscriptional levels

**Fig. 5:**
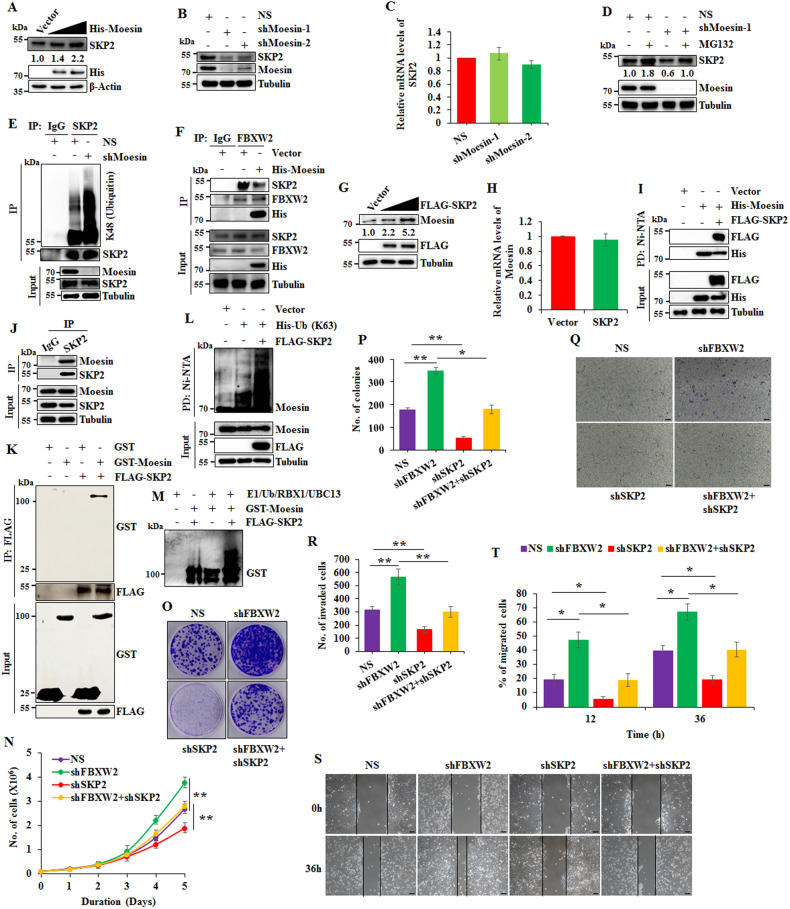
Moesin and SKP2 interact with and positively regulate each other’s expression at the posttranscriptional level. **A** Immunoblot showing the expression levels of SKP2, His-Moesin, and β-actin in MCF7 cells expressing either vector or different doses of His-Moesin. **B** Immunoblot showing the expression levels of Moesin, SKP2, and tubulin in MCF7 cells expressing either scramble shRNA (NS) or Moesin specific two independent shRNAs. **C** Real-time qRT-PCR showing relative mRNA levels of SKP2 in MCF7 cells expressing either scramble shRNA (NS) or two independent Moesin specific shRNAs. β-actin was used as internal control. **D** Control (NS) and Moesin-depleted MCF7 cells were grown in the presence or absence of 5 µM MG132 for 6 h as indicated. Whole-cell protein extracts were immunoblotted for the indicated proteins. **E** Control (NS) and Moesin-depleted MCF7 cells were grown in the presence of 5 µM MG132 for 6 h before harvesting. Whole-cell protein extracts were immunoprecipitated with either anti-IgG (control) or anti-SKP2 antibody. Immunoprecipitates were immunoblotted for K48-linked ubiquitin and input protein extracts were immunoblotted for the indicated proteins. **F** MCF7 cells were transfected with either vector or His-Moesin for 36 h. Transfected cells were then treated with 5 µM MG132 for 6 h. Whole-cell protein extracts were immunoprecipitated with anti-IgG or anti-FBXW2 antibody. Immunoprecipitates and input protein extracts were immunoblotted for the indicated proteins. **G** Immunoblot showing the expression levels of Moesin, FLAG-SKP2, and Tubulin in MCF7 cells expressing either vector or different doses of FLAG-SKP2. **H** Real-time qRT-PCR showing relative mRNA levels of Moesin in MCF7 cells expressing either vector or FLAG-SKP2. β-actin was used as internal control. **I** MCF7 cells were transfected with indicated plasmids for 36 h. Transfected cells were then treated with 5 µM MG132 for 6 h. His-Moesin were pulled down by using Ni-NTA beads from whole-cell protein extracts. Pulled down proteins and input protein extracts were immunoblotted for the indicated proteins. **J** MCF7 cells were harvested and whole-cell lysates were immunoprecipitated with either anti-IgG or anti-SKP2 antibody. Then, immunoprecipitates and input protein extracts were immunoblotted for the indicated proteins. **K** Recombinant purified GST and GST-Moesin proteins were incubated with Ni-NTA bead-bound FLAG-SKP2 at indicated combinations for 2 h at room temperature. Then, Ni-NTA beads were washed and pulled down proteins were eluted with SDS buffer. Elutes and input proteins were immunoblotted for the indicated proteins. **L** MCF7 cells were transfected with indicated plasmids for 36 h followed by addition of 5 µM MG132 for 6 h and lysed under denaturing condition. Ubiquitinated proteins were pulled down with Ni-NTA beads from whole-cell protein extracts. Pulled down proteins and input protein extracts were immunoblotted for the indicated proteins. **M** In vitro ubiquitination experiment was performed by using recombinant purified proteins. All the purified components and ATP were incubated with ubiquitination buffer (50 mM Tris, pH 8.0, 5 mM MgCl_2_, 1 mM β-mercaptoethanol, and 0.1% Tween 20) in indicated combination for 2 h at room temperature. Then, SDS sample buffer was added to stop the reaction and boiled for 5 min. The samples were then resolved in SDS-PAGE followed by western blotting with anti-GST antibody. **N** Trypan blue exclusion assay in MCF7 cells expressing either NS or shRNAs against SKP2 or FBXW2 or both. **O** Long-term colony formation assay in MCF7 cells expressing either NS or shRNAs against SKP2 or FBXW2 or both. **P** Quantification of colonies from (**O**). **Q** Invasion assay in MCF7 cells expressing either NS or shRNAs against SKP2 or FBXW2 or both. Scale bars = 100 µM. **R** Quantification of invaded cells from (**Q**). **S** Scratch wound healing assay in MCF7 cells expressing either NS or shRNAs against SKP2 or FBXW2 or both. Scale bars = 100 µM. **T** Quantification of migrated cells from (**S**).

Oncogene SKP2 is a known target of FBXW2 [14]. Serendipitously, in FBXW2 and Moesin co-expression study, we found that overexpression of Moesin leads to increased levels of SKP2 (Supplementary Fig. S4A), indicating that Moesin might protect SKP2 from its downregulation. To authenticate this observation, expression levels of SKP2 were examined following ectopic expression of Moesin. Western blotting data revealed that expression levels of SKP2 are increased in a dose-dependent manner upon Moesin overexpression (Fig. 5A). Conversely, expression levels of SKP2 are declined following depletion of Moesin in MCF7 and MDA-MB-231 cells (Fig. 5B and Supplementary Fig. S4B). However, overexpression or knockdown of Moesin does not affect the mRNA levels of SKP2 (Fig. 5C and Supplementary Fig. S4C) suggesting that Moesin positively regulates SKP2 levels at the posttranscriptional level. Then, we found that Moesin-depletion mediated attenuation of SKP2 is blocked following treatment with proteasome inhibitor MG132, indicating that SKP2 undergoes proteasomal degradation in the absence of Moesin (Fig. 5D). To substantiate our observation, we examined the polyubiquitinated levels of SKP2 in Moesin-depleted cells. Immunoblotting results revealed that K48-linked polyubiquitination of SKP2 is significantly increased upon depletion of Moesin (Fig. 5E) suggesting that Moesin stabilizes SKP2 by protecting it from proteasome-mediated degradation. Since SKP2 is a known target of FBXW2 [14], we hypothesized that Moesin prevents FBXW2-mediated proteasomal degradation of SKP2. Immunoprecipitation study shows that Moesin overexpression significantly attenuates FBXW2-SKP2 interaction (Fig. 5F), suggesting that Moesin protects SKP2 from its proteasomal degradation by FBXW2 by attenuating FBXW2-SKP2 interaction.

SKP2 is a well-known oncogene in breast cancer progression [21]. Previous studies showed that SKP2 promotes oncogenic function of oncogenes like AKT by increasing their expression level [22]. Further, our preceding results showed that Moesin protects SKP2 from proteasomal degradation. We then speculated that SKP2 might also protect Moesin from proteasomal degradation by FBXW2 to facilitate cancer progression. To examine this possibility, we assessed the expression levels of Moesin following overexpression of SKP2. Interestingly, SKP2 overexpression increases the expression levels of Moesin in a dose-dependent manner without affecting its mRNA levels (Fig. 5G, H), indicating that SKP2 and Moesin increase each other’s expression levels at the posttranslational level through a feed-forward loop. These observations prompted us to investigate the interaction of SKP2 and Moesin. Immunoblotting of Moesin-pull-down fractions revealed that Moesin interacts with SKP2 both at the exogenous and endogenous level (Fig. 5I, J). Further, their interaction was investigated using recombinant GST-Moesin and FLAG-SKP2 and immunoblotting results revealed that SKP2 directly interacts with Moesin (Fig. 5K). We then asked how SKP2 increases the expression levels of Moesin. It is reported that SKP2, being a well-known oncogenic F-box protein (FBXL1), stabilizes various oncogenic substrates via K63-linked polyubiquitination [22, 23]. So, we performed in vivo polyubiquitination assay by co-expressing FLAG-SKP2 and His-Ub-K63 constructs. The western blotting data showed that SKP2 overexpression facilitates K63-linked polyubiquitination of Moesin (Fig. 5L). To confirm this, we performed in vitro polyubiquitination assay with the purified GST-Moesin and FLAG-SKP2 and observed that SKP2 directly facilitates K63-linked polyubiquitination of Moesin (Fig. 5M). Taken together, these observations reveal that Moesin and SKP2 stabilize each other at the protein levels; Moesin prevents FBXW2 to target SKP2 and in return SKP2 stabilizes Moesin by facilitating its degradation defective ubiquitin linkage (K63-linkage).

In order to understand the biological relevance of FBXW2-Moesin-SKP2 regulation, we generated MCF7 cells with stable depletion of either FBXW2 or SKP2 or both by using specific lentivirus shRNAs (Supplementary Fig. S4D) and performed a series of phenotypic assays. The results revealed that depletion of FBXW2 increases proliferation and colony formation of MCF7 cells; in contrast, SKP2 depletion shows opposite phenotype (Fig. 5N–P). As anticipated, depletion of SKP2 in FBXW2-depleted cells resumes the proliferation and colony formation similar to NS cells. Similar results were observed in cell invasion (Fig. 5Q, R) and migration assay (Fig. 5S, T). Taken together, these observations suggest that FBXW2 prevents breast cancer progression, at least partly, by negatively regulating SKP2 levels.

### AKT prevents proteasomal degradation of Moesin by interrupting FBXW2-Moesin interaction

**Fig. 6:**
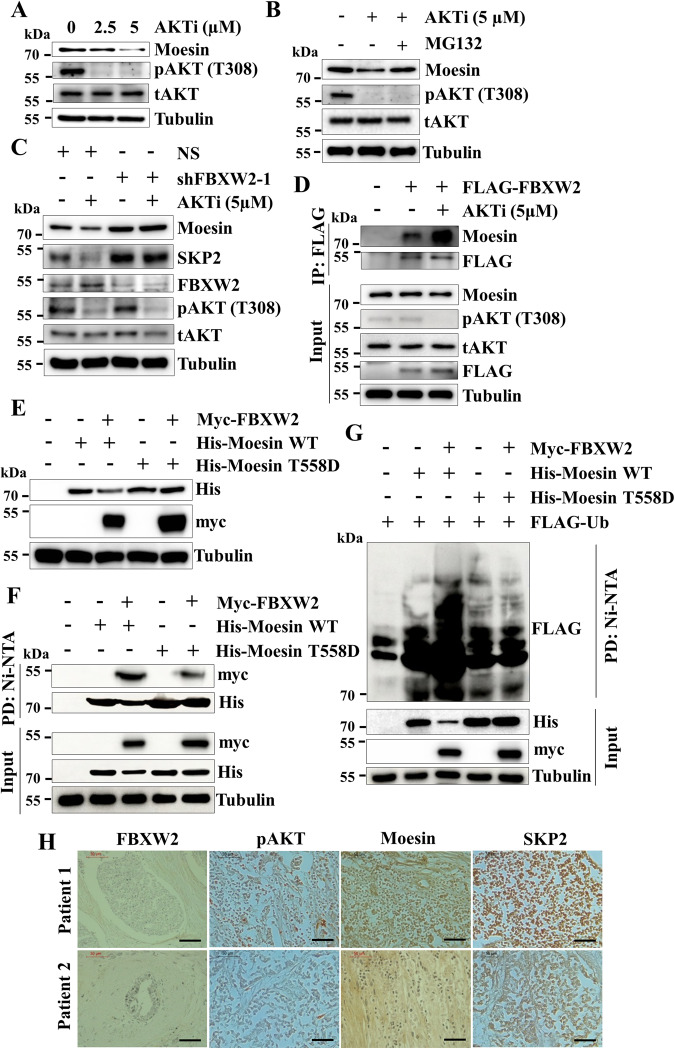
AKT stabilizes Moesin by interrupting the interaction between Moesin and FBXW2. **A** MCF7 cells were treated with increasing doses of AKT inhibitor (0, 2.5 and 5 µM) for 12 h and whole-cell protein extracts were immunoblotted for indicated proteins. **B** MCF7 cells were treated with 5 µM AKT inhibitor for 6 h followed by treatment with 5 µM of MG132 for additional 6 h as indicated. Then, whole-cell protein extracts were immunoblotted for the indicated proteins. **C** NS and FBXW2-depleted cells were grown in the presence or absence of 5 µM AKT inhibitor for 12 h and whole-cell protein extracts were immunoblotted for the indicated proteins. **D** MCF7 cells were transfected with either vector or FLAG-FBXW2 for 36 h followed by addition of 5 µM AKT inhibitor for 12 h as indicated. Whole-cell lysates were immunoprecipitated with anti-FLAG antibody. Immunoprecipitates and input protein extracts were immunoblotted for the indicated proteins. **E** MCF7 cells were transfected with indicated plasmids for 48 h. Whole-cell lysates were then immunoblotted for indicated proteins. **F** MCF7 cells were transfected with indicated plasmids for 36 h. Transfected cells were treated with 5 µM MG132 for additional 6 h and His-Moesin was then pulled down with Ni-NTA beads from whole-cell lysates. Pulled down proteins and input protein extracts were immunoblotted for the indicated proteins. **G** MCF7 cells were transfected with indicated plasmids for 36 h and lysed under denaturing condition. Transfected cells were treated with 5 µM of MG132 for additional 6 h and whole-cell lysates were then pulled down with Ni-NTA beads. Pulled down proteins and input protein extracts were immunoblotted for indicated proteins. **H** Representative IHC images showing FBXW2, Moesin, pAKT (T308) and SKP2 staining of tissue sections of HR positive breast cancer patients (*n* = 12). Scale bars = 50 µM.

Generally, SCF E3 ubiquitin ligases recognize phosphorylated substrates to ubiquitinate them [10]. Previous reports suggest that the ERM family members including Moesin become functional upon their phosphorylation [24]. Interestingly, it was shown that AKT kinase, a highly activated oncogenic kinase in breast cancer, phosphorylates both Moesin and SKP2 to activate their oncogenic potential [25, 26]. We therefore asked whether AKT kinase regulates the expression level of Moesin. Western blotting results showed that dose-dependent pharmacological inhibition of AKT reduces Moesin levels in both MCF7 and MDA-MB-231 cells (Fig. 6A and Supplementary Fig. S5A). Similarly, stable depletion of AKT in MDA-MB-231 cells also results in attenuation of Moesin levels (Supplementary Fig. S5B). These data indicate that AKT maintains high-level expression of Moesin in breast cancer. Then, we asked how AKT maintains the high expression levels of Moesin. Being a kinase, we speculated that AKT might regulate Moesin at the posttranslational level. Immunoblotting results reveal that AKT inactivation-mediated downregulation of Moesin is blocked following treatment of MG132, indicating that AKT prevents proteasomal degradation of Moesin (Fig. 6B). Proteasomal degradation of Moesin warrants its proteasomal degradation specific polyubiquitination. Interestingly, immunoblotting results show that K48-linked polyubiquitination of Moesin is increased upon AKT inhibition (Supplementary Fig. S5C).

Preceding results demonstrated that FBXW2 facilitates while AKT prevents the proteasomal degradation of Moesin. We then speculated that AKT might regulate Moesin by counteracting FBXW2-mediated Moesin regulation. For this, control and FBXW2-depleted MCF7 cells were treated with the AKT inhibitor. Immunoblotting results reveal that AKT inactivation-mediated ablation of Moesin levels is markedly blocked in FBXW2-depleted cells (Fig. 6C) indicating that FBXW2 plays key role in ablation of Moesin upon AKT inactivation. This observation prompted us to ask whether FBXW2 preferentially targets Moesin in the absence of AKT signaling. Immunoblotting of immunoprecipitates showed that FBXW2-Moesin interaction and FBXW2-mediated Moesin polyubiquitination is robustly enhanced upon silencing AKT expression or pharmacological inactivation of AKT signaling (Fig. 6D and Supplementary Fig. S5D–F). These data suggest that the oncogene AKT protects another oncogene Moesin from FBXW2-mediated proteasomal degradation.

As mentioned earlier, ERM family members including Moesin become functional upon their phosphorylation [24]. T558 of Moesin is a conserved phosphorylation site among various species [24] and a study suggests that AKT phosphorylates Moesin at T558 for its activation [25]. However, the biological significance of AKT-mediated Moesin phosphorylation at T558 in cancer is still unknown. Our previous data suggested that AKT prevents FBXW2-mediated Moesin degradation. So, we hypothesized that AKT phosphorylates Moesin at T558 to prevent its degradation by FBXW2. To understand this, we generated a phosphomimetic Moesin mutant (His-Moesin T558D) and co-expressed FBXW2 either with wild-type Moesin (His-Moesin WT) or with His-Moesin T558D. Immunoblotting data revealed that FBXW2 could degrade WT Moesin while could not degrade phosphomimetic Moesin (Fig. 6E). Further, FBXW2 shows significantly less interaction with phosphomimetic Moesin than the WT Moesin (Fig. 6F). Polyubiquitination assay revealed that FBXW2 polyubiquitinates phosphomimetic Moesin lesser than the WT Moesin (Fig. 6G). Collectively, these data reveal that AKT-mediated Moesin phosphorylation at T558 promotes its stability by attenuating its proteasomal degradation by FBXW2.

The preceding in vitro results suggest that activated AKT (pAKT) positively regulates Moesin. Moesin then positively regulates SKP2 and in turn SKP2 positively regulates Moesin. Hence, there exists an AKT-Moesin-SKP2 oncogenic axis in breast cancer, which is counteracted by FBXW2. To further validate this regulatory axis in breast cancer, we assessed the correlation among the expression levels of these four proteins (FBXW2, pAKT, Moesin and SKP2) in HR positive breast cancer patients. Interestingly, IHC results reveal the existence of a good positive correlation among AKT, SKP2 and Moesin (Fig. 6H and Supplementary Fig. S5G). As expected, we found converse correlation of FBXW2 with this oncogenic axis (Fig. 6H and Supplementary Fig. S5G). These observations suggest that FBXW2 prevents breast cancer progression by restricting the oncogenic AKT-Moesin-SKP2 axis.

## Discussion

**Fig. 7:**
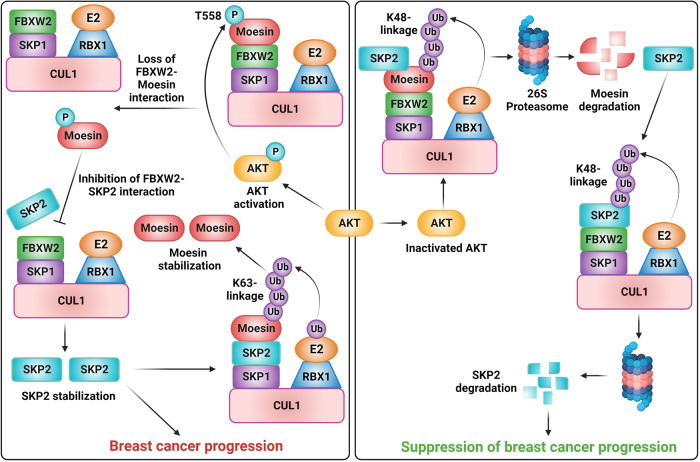
Model depicts the importance of AKT-Moesin-SKP2 axis in promoting breast cancer progression (left side) and its intervention by FBXW2 (right side). (Left) Activated AKT phosphorylates Moesin at T558 to disrupt FBXW2-Moesin interaction. Moesin then disrupts FBXW2-SKP2 interaction to promote SKP2 mediated breast cancer progression. In addition, SKP2 promotes accumulation of Moesin by promoting its K63-linked ubiquitination via a positive feed-forward loop. (Right) FBXW2 suppresses breast cancer progression by directing the proteasomal degradation of Moesin leading to further proteasomal degradation of SKP2.

Moesin functions as a potential oncogene in breast cancer by promoting cancer initiation, progression, and metastasis [6, 27–29]. Further, it was found that Moesin is highly expressed in hormone receptor positive as well as negative breast cancer patients and its high-level expression is closely associated with poor prognosis [30–32]. Our study, for the first time, reveals the underlying molecular mechanism of overexpression of Moesin in breast cancer. We identified tumor suppressor FBXW2 as the first bonafide E3 ligase for Moesin. We showed that FBXW2 acts as a negative regulator of Moesin at the posttranslational level and it is under expressed in breast cancer cell lines and patient samples (Fig. 7). Further, we observed a good converse correlation in the expression levels of Moesin and FBXW2 among the cell lines and breast cancer patients. Thus, restoration of FBXW2 expression could be a potential strategy to circumvent the potential threat by Moesin.

Previous studies showed that FBXW2 functions as putative tumor suppressor in many cancers through directing proteasomal degradation of several oncogenes including SKP2, β-catenin, and EGFR [14, 15, 17–19]. In this study, we found that oncogene Moesin is a cellular target of FBXW2. It was shown that overexpression of Moesin in multiple cancers is closely associated with activation of β-catenin [8, 33]. In addition to β-catenin, Moesin also positively regulates SKP2 at the posttranslational level. Mechanistic insight shows that Moesin prevents FBXW2-SKP2 interaction and attenuates FBXW2-mediated SKP2 polyubiquitination and degradation ultimately leading to tumor growth (Fig. 7). This is a similar observation to that of a recent study where activated Moesin competes with E3 ligase SPOP to prevent PD-L1 degradation and thereby promotes breast tumor progression [28]. These evidences suggest that Moesin might function as an upstream regulator to stabilize these oncoproteins (including SKP2), aggravate cancer progression and correlate with poor patient prognosis, and therefore could serve as a potential target for cancer therapy.

Recently, it was shown that FBXW2 suppresses breast cancer growth through controlling NF-κB p65 level [18]. It was shown that nuclear p65 functions as an oncogene [34–37]. However, its nuclear accumulation is context dependent. Nuclear accumulation of p65 mostly occurs during chemotherapy drugs treatment and acquired resistance. Moreover, nuclear accumulation of p65 is poorly observed in cancer patient samples. Further, it was reported that suppression of NF-κB can result in serious host toxicity with minimum effect on the tumor [38] and therefore p65 is not an established cancer marker to develop therapeutic drugs. On the other hand, irrespective of hormone expression status, Moesin is known to be involved in breast cancer progression and metastasis [6, 27–29, 39–43] and is highly expressed in breast cancer [28–30, 39, 44–46]. Therefore, limiting Moesin expression level in breast cancer could be beneficial for the management of cancer progression. In future, it would be interesting to understand the molecular pathways involved in attenuation of FBXW2 in cancer, which could be explored to restore the expression levels of FBXW2 to limit the Moesin expression levels.

High-level expression of Moesin is predominantly regulated at the posttranslational level in many cancers [8, 28, 29, 44, 45]. Previous study showed that AKT kinase plays crucial role in maintaining the high-level expression of Moesin [25]. Of note, AKT is highly activated in majority of breast cancer irrespective of subtypes. In the present study, we showed that AKT phosphorylates Moesin at Thr-558 position to prevent its association with FBXW2. Thus, AKT is playing critical role in maintaining high-level expression of Moesin by blocking its proteasomal degradation by FBXW2. In addition to AKT, present study reveals that oncogene SKP2 plays critical role on the proteasomal stability of Moesin by promoting Lys-63 linked ubiquitination. Further, SKP2 also plays crucial role in AKT activation by facilitating its Lys-63 linked ubiquitination [22]. In return, AKT stabilizes Moesin, which protects SKP2 from proteasomal degradation by FBXW2.

Overall, our study established tumor suppressor FBXW2 as a bonafide E3 ligase for Moesin in breast cancer. It also uncovers a hitherto unknown oncogenic AKT-Moesin-SKP2 axis that could be explored to combat cancer pathogenesis.

## Materials and methods

### Cell lines

Human embryonic kidney cell line HEK-293T and human breast epithelial cell line MCF10A were a kind gift from Prof. Michael R. Green (University of Massachusetts Medical School, USA). All the human breast cancer cell lines (MCF7, T47D, SKBR3, MDA-MB-453, MDA-MB-468, and MDA-MB-231) were obtained from National Centre for Cell Science cell repository. Murine breast cancer cell line 4T1 was a generous gift from Dr. Mohan R. Wani (National Centre for Cell Science, India). MCF10A cells were grown in DMEM/F-12 supplemented with 5% horse serum (Gibco, 16050-122), 100 ng/ml cholera toxin, 20 ng/ml EGF, 0.5 mg/ml hydrocortisone and 10 µg/ml insulin. HEK-293T and MCF7 cells were grown in DMEM (Gibco—12800017) whereas T47D, SKBR3, MDA-MB-453, MDA-MB-468, MDA-MB-231, and 4T1 breast cancer cells were grown in RPMI medium (Gibco—31800022) as monolayer supplemented with 10% Fetal Bovine Serum (Himedia—RM9955). All the cell lines were cultured with 100 µg/ml penicillin and 100 µg/ml streptomycin (Life Technologies, Inc., 15140-122s) at 37 °C, 5% CO_2_ atmosphere in a humid condition. Mycoplasma contamination of cell lines was regularly tested.

### Plasmids

pCMV-Entry-Myc-DDK-FBXW2 was purchased from OriGene (RC205840). FBXW2 was also subcloned in pCMV-Myc-FBXW2 using primers listed in Supplementary Table S3. pCMV3-His-Moesin was purchased from Sino Biological (HG13866-CH). pGEX4T1-Moesin was purchased from Addgene (Cat# 11637). FBXW2 was subcloned in pET28a at EcoRI site using primers listed in Supplementary Table S3. Wild-type ubiquitin (Ub) and mutant Ub constructs (His-Ub (K11), His-Ub (K48), His-Ub (K63)) were kind gifts from Dr. Wuhan Xiao (Chinese Academy of Science, China). F-box motif-deleted FBXW2 (ΔF-FBXW2) mutant was generated in the pCMV-Entry vector using primers listed in Supplementary Table S3. pCMV-Entry-Myc-DDK-SKP2 was purchased from OriGene. psPAX2 (Addgene plasmid # 12260) and pMD2.G (Addgene plasmid # 12259) were a kind gift from Prof. Didier Trono (Ecole Polytechnique Fédérale de Lausanne, Switzerland). Phosphomimetic mutant of Moesin at T558 (T558D) was generated in pcDNA4/V5-His A at AgeI restriction site using site directed mutagenesis (SDM) using primers listed in Supplementary Table S3.

### Antibodies

Antibodies against FBXW2 (sc-160326), SKP2 p45 (sc-7164), pAKT (S473) (sc-293125), GAPDH (sc-32233) and His (sc-8036) were obtained from Santa Cruz Biotechnology. Antibodies against pAKT (T308) (9275), pAKT (S473) (4058), Ubiquitin-K48 (8081), Cullin1 (4995) and HRP-conjugated anti-mouse (7076) secondary antibodies were purchased from Cell Signaling Technology. Antibodies against FLAG (F1804), Tubulin (T5168) and β-Actin (A2228) were purchased from Sigma. Anti-Myc (11667149001) antibody was from Roche and anti-GST (BB-AB0020) was from Bio Bharati, anti-FBXW2 (11499-1-AP) from Protein Tech, anti-Moesin (PA5-34666) from Thermo Fisher Scientific and HRP-conjugated anti-rabbit (1706515) secondary antibody was purchased from Bio-Rad.

### Transfection

Cells were transfected with desired cDNA constructs as described previously [47]. In brief, indicated plasmids were diluted in 150 mM NaCl and then Polyethyleneimine (PEI, 3 μg of PEI/μg of DNA) (PEI-25K, Polysciences-23966-1) was added. Transfection mixtures were incubated for 15 min at room temperature. In the meantime, complete media was replaced by media containing 0.5% FBS. After incubation, the transfection mixture was added to the cells and was incubated at 37 °C under 5% CO_2_ condition. At 12 h post transfection, the 0.5% FBS containing media was replaced with complete media, and transfected cells were collected at the indicated periods. Cells were treated with 10 μM proteasome inhibitor MG132 (Calbiochem, 474790) for 6 h whenever needed in the study.

### Generation of stable knockdown cells

Stable knockdown of FBXW2, Moesin, and AKT in the indicated cell lines was generated using the lentiviral transduction method as described previously [48]. In brief, HEK-293T cells were transfected with either the scrambled short hairpin RNA (shRNA) (NS) or 2 independent shRNAs (against FBXW2, Moesin, or AKT) (Supplementary Table S3) along with lent-viral packaging plasmids psPAX2 and pMD2.G using PEI. Virus-containing media was collected after 48 h of transfection, filtered through a 0.45 μM syringe filter and infected the host cells (MCF10A, MCF7, or MDA-MB-231 as indicated) using 8 μg/ml polybrene (Sigma, TR-1003). Transduced/infected cells were then grown in the presence of 1 µg/ml puromycin to select the transduced cells. Knockdown efficiency was determined through western blotting.

### Western blotting

Western blotting was performed as described previously [49]. Briefly, cells were collected, washed with ice-cold PBS buffer, and then lysed on ice for 20 min with lysis buffer (50 mM Tris-Cl, 5 mM EDTA, 250 mM NaCl, 50 mM NaF, 0.5 mM sodium vanadate, 0.5% Triton X-100 and protease inhibitor cocktail (Pierce-A32965)) followed by centrifugation at 20,000 × *g* for 20 min. The supernatant containing the total protein extract was collected. Protein concentration was measured using the Bradford method using bovine serum albumin (BSA) as the standard [50]. Then, samples were prepared in sample buffer (0.12% bromophenol blue, 250 mM Tris-HCl pH 6.8, 10% SDS, 50% glycerol, and β-mercaptoethanol) for SDS-PAGE. Cell lysates (20–25 μg/lane) were resolved in SDS-PAGE. The resolved proteins were then transferred onto an activated polyvinylidene difluoride (PVDF) membrane in buffer (26 mM Tris, 184 mM Glycine and 20% methanol (v/v), pH 8.8). The membrane was then blocked with 3% skimmed milk, followed by incubation with indicated primary antibody overnight at 4 °C. The next day, the membrane was washed 3–5 times with TBST (TBS buffer with 0.1% Tween 20), and then incubated with respective HRP-conjugated secondary antibodies for 1 h at room temperature. Finally, the membrane was washed again 3–5 times with TBST, and proteins were detected by AMERSHAM ImageQuant 800 (GE Healthcare) using a sensitive chemiluminescence substrate kit (Pierce, West Pico-34578 and West Femto-34096).

### Immunoprecipitation (IP)

Immunoprecipitation experiments were performed as described previously [48]. Briefly, 600–800 μg of protein extract was mixed with 2–3 μg of desired primary antibody in 600 μl IP lysis buffer (50 mM Tris-Cl, 5 mM EDTA, 250 mM NaCl, 50 mM NaF, 0.5 mM sodium vanadate and 0.05% Triton X-100 containing protease inhibitors) and incubated overnight at 4 °C with gentle rocking. The next day, washed recombinant protein G agarose beads (Pierce, 15920-010) were added to the antigen-antibody mixture and incubated for 1.5 h with gentle rocking at 4 °C. The beads were then washed three times with IP lysis buffer with gentle rocking for 5 min each. Finally, beads were resuspended in an SDS sample buffer and boiled for 5 min to release the immunoprecipitates in the solution. The immunoprecipitates were then resolved in SDS-PAGE and the gel was further processed for either western blotting or mass spectrometry.

### Mass spectrometry

To find out the FBXW2-interactomes, mass spectrometry was performed as described previously [51]. Briefly, the whole-cell lysates were prepared and IP was set up as described above. The immunoprecipitates were resolved in a 16 cm SDS-PAGE by electrophoresis. The gel was stained overnight with Gel Code Blue stain (Pierce, 24590). The unique bands found in FBXW2 immunoprecipitates lane were excised, in-gel digested with trypsin, followed by desalting using a Millipore Zip Tip C18 column and finally subjected to LC-MS/MS. The LC-MS/MS experiment was performed on an Orbitrap fusion mass spectrometer (Thermo Scientific) coupled to the Easy Nano LC system. Peptides were separated using an Easy-spray PEPMAP RSLC C182 µm, 50 cm (Thermo Scientific).

### Cycloheximide pulse-chase assay

Cycloheximide pulse-chase assay was performed as described previously [52]. MCF7 cells were treated with 100 μg/ml cycloheximide (Sigma-C1988) for the indicated time periods. Whole-cell lysates were prepared and resolved in SDS-PAGE followed by western blotting with the indicated primary antibodies. The protein levels of Moesin were normalized with tubulin (loading control) at each time point. The Moesin: tubulin ratio was set as 1 at the “zero” time point. The ratio was calculated at other time points with respect to the “zero” time point.

### In vivo polyubiquitination assay

MCF7 cells were transfected with the indicated plasmids for 36 h. Transfected cells were then grown in the presence of 5 μM MG132 for 6 h. Protein extracts (800 µg) were incubated with Ni-NTA beads in IP lysis buffer (50 mM Tris-Cl, 5 mM EDTA, 250 mM NaCl, 50 mM NaF, 0.5 mM sodium vanadate and 0.05% Triton X-100 containing protease inhibitors) for overnight at 4 °C with gentle rocking. The next day, the beads were washed with IP lysis buffer three times (5 min each) with gentle rocking. Finally, beads were resuspended in SDS sample buffer and boiled for 5 min to release the immunoprecipitates into the solution. The immunoprecipitates were then resolved in SDS-PAGE followed by western blotting with the indicated primary antibodies. We also performed ubiquitination assay under denaturing condition as described previously [53].

### Protein purification

Recombinant proteins (GST and GST-Moesin) were purified using a bacterial system (BL21 strain of E. coli) as described previously [47]. Briefly, BL21 cells were transformed with the desired recombinant plasmids and grown in LB media under antibiotic selection (Ampicillin/Kanamycin) at 37 °C until they reached the early log phase (λ600 ~ 0.4–0.6). Then, proteins were induced by adding IPTG (0.1 mM for Moesin, 0.5 mM for FBXW2, and 0.25 mM for GST) and allowed to grow further for 6–8 h. Cells were harvested by centrifugation at 6000 × *g* for 10 min and resuspended in lysis buffer (50 mM Tris, pH 8.0, 100 mM NaCl, 10 mM MgCl_2_, 0.1% β-mercaptoethanol and 1 mM phenyl-methyl sulfonyl fluoride) containing 0.4 mg/ml lysozyme. The cell suspension was incubated for 30 min on ice and then sonicated for six cycles with a 20-s pulse (i.e., 20 s ON and 20 s OFF). The cells were centrifuged at 12,000 × *g* for 30 min at 4 °C. The supernatant containing GST or GST-Moesin was incubated with anti-GST agarose beads. The recombinant proteins were eluted with 10 mM glutathione (pH 8.0). Similarly, His-FBXW2 was expressed in the presence of 0.5 mM IPTG and purified using Ni-NTA beads (Sigma-5893801001). Protein concentration was determined by the Bradford method using BSA as the standard [50].

### In vitro pull-down assay

Direct protein-protein interaction between FBXW2 and Moesin was determined using in vitro pull-down assay as described previously [47]. Briefly, Ni-NTA beads-bound His-FBXW2 was incubated with purified GST or GST-Moesin for 6 h at 4 °C in combinations as indicated. For control, Ni-NTA beads were also incubated with either purified GST or GST-Moesin. Then, the beads were washed thrice (5 min each) with IP lysis buffer, and proteins were eluted by boiling with SDS sample buffer and resolved in SDS-PAGE, followed by western blotting with the indicated primary antibodies.

### In vitro polyubiquitination assay

All the components (E1, K48-specific E2 (Cdc34), SKP1, Cullin1, and Ubiquitin (Ub)) required for the ubiquitination reaction were purified using a bacterial expression system as described above. RBX1 was purified using the mammalian expression system. All the purified components and ATP were incubated with ubiquitination buffer (50 mM Tris, pH 8.0, 5 mM MgCl_2_, 1 mM β-mercaptoethanol, and 0.1% Tween 20) in indicated combination for 2 h at room temperature. Then, SDS sample buffer was added to stop the reaction and boiled for 5 min. The samples were then resolved in SDS-PAGE followed by western blotting with anti-GST antibody.

### Quantitative real-time PCR (qRT-PCR)

Total RNA was isolated from cells using TRIzol according to the manufacturer’s protocol (Invitrogen, 10296028). One μg of isolated RNA was used to synthesize the cDNA using PrimeScript 1st strand cDNA Synthesis Kit (Takara, 6210A). Real-time qRT-PCR was then performed using SYBRmix (Takara, RR820). Primers used in the study is listed in Supplementary Table S5. β-actin was used as the internal control.

### Trypan blue exclusion cell counting assay

MCF7 cells expressing either NS or shRNAs against Moesin or FBXW2 or both (0.1 × 10^6^ cells/35 mm petri dish) were seeded. After every 24 h, cells were trypsinized and counted for 5 consecutive days using trypan blue dye. Graph was plotted using MS Excel. Cell counting was done in the same way for MCF7 cells expressing either NS or shRNAs against SKP2 or FBXW2 or both and for 4T1 cells expressing either NS or shRNAs against Moesin or FBXW2 or both.

### Colony formation assay

Colony formation assay was performed as described previously [51]. MCF7 cells expressing either NS or shRNAs against Moesin or FBXW2 or both (3 × 10^3^ cells/35 mm petri dish) were seeded. The cells were allowed to grow and form colonies for up to 3 weeks. The colonies were fixed with 3.7% formaldehyde and then stained with 0.1% crystal violet. Images were taken by scanning the petri dishes. Colonies were counted by ImageJ software and the graph was plotted using MS Excel. Colony formation assay was performed in the similar way for MCF7 cells expressing either NS or shRNAs against SKP2 or FBXW2 or both and for 4T1 cells expressing either NS or shRNAs against Moesin or FBXW2 or both.

### Soft agar colony formation assay

Soft agar long-term colony formation assay was performed as described previously [51]. Briefly, 35 mm petri dishes were filled with 0.6% base agar containing complete DMEM media (i.e., DMEM media containing 10% FBS) and allowed to solidify. The, MCF7 cells expressing either NS or shRNAs against Moesin or FBXW2 or both (3 × 10^3^ cells/35 mm petri dish) were resuspended in 0.3% top agar and placed on the top of the base agar. The cells were allowed to grow and form colonies for 3 weeks. Media was replaced every 2–3 days. Colonies were then stained with 0.1% crystal violet. Images were taken using an inverted phase contrast microscope (Olympus IX71, Shinjuku, Tokyo, Japan). Colonies were counted by ImageJ software and the graph was plotted using MS Excel.

### Wound healing migration assay

Wound healing cell migration assay was performed as described previously [54]. In brief, MCF7 cells expressing either NS or shRNAs against Moesin or FBXW2 or both were grown in 35 mm petri dishes for 24 h. Then, a scratch wound was created using a 10 µl pipette tip, and the cell debris was washed out with PBS. To block the contribution of cell proliferation in the cell migration process, the cells were grown in 0.5% DMEM media. The wound edges were photographed after indicated periods with an Olympus microscope and the scratch widths were analyzed using ImageJ software. Wound healing cell migration assay was performed in the similar way for MCF7 cells expressing either NS or shRNAs against SKP2 or FBXW2 or both and for 4T1 cells expressing either NS or shRNAs against Moesin or FBXW2 or both.

### Invasion assay

Invasion assay was performed as described previously [51]. In brief, MCF7 cells expressing either NS or shRNAs against Moesin or FBXW2 or both were serum-starved for 24 h, and 100,000 cells were then suspended in 200 μl of media containing 0.5% FBS in the upper chamber. In total, 600 μl Media containing 10% FBS was added to the lower chamber and cells were incubated for 36 h. Then, invaded cells were fixed with chilled methanol for 20 min, and stained with 0.5% crystal violet for 30 min. Different fields were randomly photographed using Nikon (ECLIPSE Ti2), and total number of invaded cells was plotted in graph using MS Excel. Invasion assay was performed in the similar way for MCF7 cells expressing either NS or shRNAs against SKP2 or FBXW2 or both and for 4T1 cells expressing either NS or shRNAs against Moesin or FBXW2 or both.

### Orthotropic mice model

Xenograft assay was performed in orthotropic mice model according to approved protocol by institutional animal ethics committee of National Centre for Cell Science. Briefly, 2 × 10^6^ 4T1 cells (NS, FBXW2 knocked down, Moesin knockdown and FBXW2 and Moesin co-knocked down cells) were injected in the mammary fat pads of NOD-SCID female mice (4–6 weeks old) (*n* = 5 for each group of mice). Upon palpable xenograft formed, tumor volume and mice weight were determined on every 3rd day by the formula, volume = (length*width^2^)/2.

### Immunohistochemistry (IHC)

Primary malignant breast tumor specimens were collected upon receiving the signed consent from all the participants (HR positive, *n* = 19; HER2-enriched, *n* = 3 and TNBC, *n* = 3). The study was approved by Institutional Ethics Committee of Saroj Gupta Cancer Centre & Research Institute, Kolkata, under regulation of the Govt. of India (Registration No. ECR/250/Inst/WB/2013/RR-20). Formalin-fixed and paraffin-embedded malignant breast tissue as well as mice tumor tissue specimens were cut into 5 μM sections. The sections were processed at 65 °C for 20 min, followed by de-paraffinization was done in two subsequent incubations with xylene and rehydration applying decreasing grades of ethanol. Temperature mediated antigen retrieval was performed using citrate buffer (10 mM sodium citrate, 0.5% Tween 20, pH 6.0) and the endogenous peroxidase was chemically quenched with 0.3% H_2_O_2_ (Merck). The sections were then incubated in primary antibodies against FBXW2 (11499-1-AP, Protein Tech), Moesin (PA5-34666, Thermo Fisher Scientific), SKP2 (sc-7164, Santa Cruz Biotechnology) and pAKT (T308) (13038, Cell Signaling Technology) at a dilution of 1:100 for overnight followed by incubation in HRP-conjugated secondary antibody (Sigma) for 2 h. The immuno-complexes were then visualized using diaminobenzidine (Sigma) and the nuclei were counterstained using Mayer’s hematoxylin (Merck). The sections were subsequently dehydrated using increasing grades of ethanol and subsequently mounted using DPX (Merck). Images were acquired using Leica DM750 light microscope at ×40 magnification and the data were processed using Leica LAS EZ version 3.4 software.

### Determining expression of FBXW2 and Moesin and breast cancer patient survival using online databases

The mRNA expression of FBXW2 and Moesin was determined in the TCGA breast cancer patients using a publicly available online database UALCAN (http://ualcan.path.uab.edu/analysis.html). The correlation between the expression of FBXW2/Moesin and recurrent-free survival (RFS) of breast cancer patients was determined using the database Kaplan–Meier (KM) plotter (https://kmplot.com/analysis/index.php?p=service&cancer=breast). Further, the correlation between the expression of low FBXW2/high Moesin and high FBXW2/low Moesin and the RFS of breast cancer patients was determined using the dataset GSE3494 and the survival curve was plotted using GraphPad Prism software.

### Statistical analysis

All the experiments in the study were performed at least three times and data were presented as mean ± S.D. The two-tailed Student’s *t* test was used to determine the statistical significance. *p* < 0.001(***), *p* < 0.01 (**), and *p* < 0.05 (*) were considered statistically significant.

### Supplementary information

Supplementary Information

## Data Availability

All datasets generated and analyzed during this study are included in this published article and its Supplementary Information files. Additional data are available from the corresponding author on reasonable request.
