# Supplementary material for: FBXW2 suppresses breast tumorigenesis by targeting AKT-Moesin-SKP2 axis
Source: Cell Death Dis. 2023 Sep 22;14(9):623. doi: 10.1038/s41419-023-06127-x (PMC10517019; doi:10.1038/s41419-023-06127-x)
Supplement: Supplementary file 1 — Supplementary Information [file 41419_2023_6127_MOESM1_ESM.pdf]

FBXW2 suppresses breast tumorigenesis by targeting AKT-Moesin-SKP2 axis

Ganesh Kumar Barik[1,2], Osheen Sahay[1,2], Anindya Mukhopadhyay[3], Rajesh Kumar Manne[1], Sehbanul Islam[1], Anup Roy[4], Somsubhra Nath[3,5] and Manas Kumar Santra[1*]

Supplementary Tables

Supplementary Table S1. Interactomes of FBXW2 (obtained from IP/MS)

Sr. No.	Protein Name	Gene Symbol	No. of Peptides	Sequest Score
1	**F-box/WD repeat-containing protein 2**	**FBXW2**	7	21.76
2	ATP synthase subunit alpha	ATP5A1	5	9.12
3	Zinc finger Ran-binding domain-containing protein 2	ZRANB2	4	6.78
4	Heterogeneous nuclear ribonucleoprotein H	HNRNPH1	4	8.47
5	RuvB-like 2	RUVBL2	2	4.19
6	RuvB-like 1	RUVBL1	1	2.38
7	**Moesin**	**MSN**	1	1.66
8	Serine/arginine-rich splicing factor 6	SRSF6	1	2.08
9	Lactotransferrin	LTF	1	1.94
10	Serine hydroxymethyltransferase, cytosolic	SHMT1	1	1.87
11	Heat shock 70 kDa protein 6	HSPA6	2	1.82
12	Heparin cofactor 2	SERPIND1	1	1.8
13	Bridging integrator 2	BIN2	1	1.68

14	Etoposide-induced protein 2.4 homolog	EI24	1	2.16
15	78 kDa glucose-regulated protein	HSPA5	2	2
16	Nucleophosmin	NPM1	1	1
17	Isoform 2 of Eukaryotic translation initiation factor 5A-1	EIF5A	1	1
18	Histone H2B	HIST1H2BN	2	2
19	Probable ATP-dependent RNA helicase DDX17	DDX17	2	2
20	Histone H4	HIST1H4A	1	1
21	Histone H2A.Z	H2AFZ	1	1
22	Chromobox protein homolog 1	CBX1	1	1
23	Peroxiredoxin-1	PRDX1	1	1
24	Pescadillo homolog	PES1	1	1
25	Heat shock protein HSP 90-beta	HSP90AB1	1	1
26	Isoform 2 of Heterogeneous nuclear ribonucleoprotein K	HNRNPK	2	2
27	Trypsin-3	PRSS3	1	1
28	Isoform 4 of Myosin-10	MYH10	2	2
29	Profilin-1	PFN1	2	2
30	Isoform 1 of Protein POF1B	POF1B	1	1
31	Lamin-B1	LMNB1	1	1
32	Phenylalanine--tRNA ligase beta subunit	FARSB	2	2
33	T-complex protein 1 subunit gamma	CCT3	1	1
34	Golgi resident protein GCP60	ACBD3	1	1
35	Cyclin-Y-like protein 2	CCNYL2	1	1
36	Solute carrier family 25 member 36	SLC25A36	1	1
37	Serine/threonine-protein kinase 36 (Fragment)	STK36	1	1

38	Elongation factor 1-beta	EEF1B2	1	1
39	Pyruvate kinase PKM	PKM	2	2
40	POTE ankyrin domain family member B3	POTEB3	1	1
41	SH3 domain and tetratricopeptide repeat-containing protein 1	SH3TC1	1	1
42	Transient receptor potential cation channel subfamily V member 5	TRPV5	1	1
43	Shugoshin 2	SGO2	1	1
44	ER degradation-enhancing alpha-mannosidase-like protein 1	EDEM1	1	1
45	E3 ubiquitin-protein ligase E3D	UBE3D	1	1
46	Zinc finger protein 250	ZNF250	1	1
47	Rho guanine nucleotide exchange factor 17	ARHGEF17	1	1
48	Probable tRNA N6-adenosine threonylcarbamoyltransferase, mitochondrial	OSGEPL1	1	1
49	Rootletin	CROCC	1	1
50	Desmocollin-3	DSC3	1	1
51	Nucleolin	NCL	2	2
52	U3 small nucleolar ribonucleoprotein protein IMP4	IMP4	1	1
53	Mastermind-like protein 2	MAML2	1	1
54	Dual specificity testis-specific protein kinase 1	TESK1	1	1
55	Transmembrane protein 145 (Fragment)	TMEM145	1	1
56	Protein DEK	DEK	1	1
57	Carbonic anhydrase 14 (Fragment)	CA14	1	1
58	Ankyrin repeat and protein kinase	ANKK1	1	1

	domain-containing protein 1			
59	RNA-binding protein 39	RBM39	1	1
60	5-methylcytosine rRNA methyltransferase	NSUN4	1	1
61	60S ribosomal protein L13a (Fragment)	RPL13A	1	1
62	DNA topoisomerase	TOP3B	1	1
63	GDH/6PGL endoplasmic bifunctional protein	H6PD	1	1
64	Protein AHNAK2	AHNAK2	1	1
65	Synemin	SYNM	1	1
66	Serine protease HTRA2, mitochondrial	HTRA2	1	1
67	DnaJ homolog subfamily B member 6	DNAJB6	1	1
68	Zinc finger and BTB domain-containing protein 4	ZBTB4	1	1
69	Isoform p21 of 7,8-dihydro-8-oxoguanine triphosphatase	NUDT1	1	1
70	Leucine zipper putative tumor suppressor 3	LZTS3	1	1
71	Homeobox protein HMX2	HMX2	1	1
72	Isoform 4 of Metastasis suppressor protein 1	MTSS1	1	1
73	Jouberin	AHI1	1	1
74	Isoform 5 of Afadin	AFDN	1	1
75	Protein disulfide-isomerase A2	PDIA2	1	1
76	Inositol polyphosphate 1-phosphatase	INPP1	1	1
77	Pregnancy-specific beta-1-glycoprotein 5	PSG5	1	1
78	Adenomatous polyposis coli protein 2	APC2	1	1
79	A disintegrin and metalloproteinase with thrombospondin motifs 20	ADAMTS20	1	1

80	Rabphilin-3A	RPH3A	1	1
81	Angiopoietin-1 receptor	TEK	1	1
82	A-kinase anchor protein 4	AKAP4	1	1
83	Inositol 1,4,5-trisphosphate receptor type 3	ITPR3	1	1
84	Isoform 2 of DNA repair protein RAD50	RAD50	1	1

Supplementary Table S2. Oncogenic interactomes of FBXW2 (obtained from IP/MS)

**Sr. No.**	**Protein Name**	**Gene Symbol**	**No. of Peptides**	**Sequest Score**
1	ATP synthase subunit alpha	ATP5A1	5	9.12
2	Heparin cofactor 2	SERPIND1	1	1.8
3	**Moesin**	**MSN**	1	1.66
4	RuvB-like 1	RUVBL1	1	2.38
5	RuvB-like 2	RUVBL2	2	4.19
6	Serine/arginine-rich splicing factor 6	SRSF6	1	2.08
7	Serine hydroxymethyltransferase, cytosolic	SHMT1	1	1.87

Supplementary Table S3. Primers used for cloning

**Primer Name**	**Sequence (5'-3')**
pCMV-Myc-FBXW2 F	CGACCGAGATCTCTCGAGGTATGGAGAGAAAGGACTTTGAG
pCMV-Myc-FBXW2 R	CTCGCCGGTACCTCGAGAGCCGTGCTCCTTCCACAACACC

pET28a - FBXW2 F	TCGCGGATCCGAATTCATGGAGAGAAAGGACTTTGAG
pET28a - FBXW2 R	GACGGAGCTCGAATTCTCAGCCGTGCTCCTTCCACAAC
pCMV-Entry-FBXW2 WT F	AGATCTGCCGCCGCGATCGCATGGAGAGAAAGGACTTTGAG
pCMV-Entry-FBXW2 WT R	GCGGCCGCGTACGCGTGCCGTGCTCCTTCCACAACACC
pCMV-Entry-FBXW2 ΔF F	CTCCTCAAGTGTAAAAATTTGGGCTGGCAG
pCMV-Entry-FBXW2 ΔF R	CAAATTTTTACACTTGAGGAGAGTCTCTAGG
pcDNA4/V5-His A Moesin WT F	TTCTACGCGTACCGGTATGGCCAAAACGATCAGTGTGCGTG
pcDNA4/V5-His A Moesin T558D R	GGTGATGATGACCGGTACCCATAGACTCAAATTCGTCAATGCGC TGCTTGGTGTTGCCCTGCCGGATCTGGCGCAGGTCCTTGTATTTG TCTCGGCC

Supplementary Table S4. Sequence of shRNAs

Gene Name	shRNA sequence (5' --- 3')
FBXW2 (Human)	AATGACGAGGTTTCAAAGG
FBXW2 (Human)	TTCATAGGCAACCAATTAG
FBXW2 (Mouse)	TCATTTTTCTGCAAGTCCG
FBXW2	TTAATAACAGACTCCATGT

(Mouse)	
Moesin (Human)	AATTCCAGGTTTCAATTTAGC
Moesin (Human)	AAGTCGCCATACTTAGACTGG
Moesin (Mouse)	AAGTCACCATACTTAGACTGG
Moesin (Mouse)	AAATGCTCTATTCTAGAGAGC
AKT	TCTTGAGGATCTTCATGGC
AKT	TCACCAGGATCACCTTGCC
SKP2	TATCATAGCACCGACTGAG
SKP2	TATCACTTAAGTCTAGATG

Supplementary Table S5. Primers used for real time qRT-PCR

**Primer Name**	**Sequence (5’-3’)**
β-Actin F	GCATGGAGTCCTGTGGCATC
β-Actin R	TTCTGCATCCTGTCGGCAAT
Moesin F	GAGGATGCTGTCCTGGAATATC
Moesin R	CACAGCTCTGAGCCTTTCTT
SKP2 F	GGGAAGTAATGTCGACGAAAGA
SKP2 R	CTTGTTGCTTTGGCTGTTGT

Supplementary Figure Legends

Supplementary Figure S1. FBXW2 directly interacts and conversely correlates with Moesin in breast cancer. A. Whole cell lysates from MCF7 cells expressing either empty vector or FLAG-FBXW2 were immunoprecipitated with anti-FLAG antibody. Elutes and whole cell lysates were resolved in SDS-PAGE, stained with Gel Code Blue stain, specific bands from IP lane excised and trypsin digested and then subjected to LC-MS/MS. Gel bands showing the position of FBXW2 and Moesin were indicated by black arrows. M, molecular weight marker. **B.** Whole cell lysates from MDA-MB-231 cells expressing either empty vector or myc-FBXW2 were immunoprecipitated with anti-myc antibody. Elutes and whole cell lysates were resolved in SDS-PAGE and immunoblotted for the indicated proteins. **C, D.** MCF7 (C) and MDA-MB-231 (D) cells were harvested and whole cell lysates were immunoprecipitated with either anti-IgG or anti-Moesin antibody. Then, immunoprecipitates and input protein extracts were immunoblotted for the indicated proteins. **E.** Quantification of protein expression of FBXW2 and Moesin shown in Fig. 1D. Their expression levels were normalized with that of loading control β-Actin. The arbitrary value in MCF10A was made as 1 and the value in other cell lines were calculated with respect to that of MCF10A. R^2, Regression coefficient. **F.** Quantification of IHC data in Fig. 1E. **G.** Correlation between the expression of low FBXW2/high Moesin and high FBXW2/low Moesin and the RFS of breast cancer patients

Supplementary Figure S2. FBXW2 inhibits breast cancer progression through Moesin. A. Whole cell lysates from MCF7 cells expressing either NS or shRNAs against Moesin or FBXW2 or both were immunoblotted for the indicated proteins. **B.** Trypan blue exclusion assay of 4T1 cells expressing either NS or shRNAs against Moesin or FBXW2 or both. **C.** Long term colony formation assay of 4T1 cells expressing either NS or shRNAs against Moesin or FBXW2 or both. **D.** Quantification of colonies from panel C. **E**. Invasion assay of 4T1 cells expressing either NS or shRNAs against Moesin or FBXW2 or both. Scale bars = 100 µM. **F.** Quantification of invaded cells from panel E. **G.** Scratch wound healing assay of 4T1 cells expressing either NS or shRNAs against Moesin or FBXW2 or both. Scale bars = 100 µM. **H.** Quantification of invaded cells from panel G. **I.** Tumor weight (gm) of 4T1 cells expressing either NS or shRNAs against Moesin or FBXW2 or both. **J.**

Body weight of NOD-SCID female mice injected with 4T1 cells expressing either NS or shRNAs against Moesin or FBXW2 or both. **K.** Tumor tissues of 4T1 cells expressing either NS or shRNAs against Moesin or FBXW2 or both were homogenized, lysed in lysis buffer, whole cell lysates were immunoblotted for the indicated proteins. GAPDH was taken as loading control.

Supplementary Figure S3. FBXW2 negatively regulates Moesin through proteasome. A. Whole cell protein extracts of MDA-MB-231 cells expressing either vector or myc-FBXW2 were immunoblotted for the indicated proteins. **B.** Real time qRT-PCR showing relative mRNA levels of Moesin in MDA-MB-231 cells expressing either vector or myc-FBXW2. **C.** Immunoblot showing the expression levels of Moesin, FBXW2 and β-Actin in MCF10A cells expressing either scramble shRNA (NS) or FBXW2 specific two independent shRNAs. **D.** MDA-MB-231 cells were transfected with either empty vector or myc-FBXW2. At 36 h post transfection cells were grown in the presence or absence of 5 μM of MG132 for additional 6 h and whole cell protein extracts were immunoblotted for the indicated proteins. **E.** MCF7 cells expressing vector or myc-FBXW2 were treated with 100 μg/ml of cycloheximide (CHX) for the indicated time periods and whole cell lysates were immunoblotted for the indicated proteins. **F**. Quantification of relative expression levels of Moesin in panel E.

Supplementary Figure S4. Moesin positively regulates SKP2. A. Immunoblot showing the expression levels of SKP2, His-Moesin, FLAG-FBXW2, and Tubulin in MCF7 cells expressing either vector or His-Moesin or different doses of FLAG-FBXW2. **B.** Immunoblot showing the expression levels of SKP2, Moesin, and Tubulin in MDA-MB-231 cells expressing either scramble shRNA (NS) or Moesin specific two independent shRNAs. **C.** Real time qRT-PCR showing relative mRNA levels of SKP2 in MDA-MB-231 cells expressing either scramble shRNA (NS) or two independent Moesin specific shRNAs. β-actin was used as an internal control. **D.** Whole cell lysates from MCF7 cells expressing either NS or shRNAs against SKP2 or FBXW2 or both were immunoblotted for the indicated proteins.

Supplementary Figure S5. AKT stabilizes Moesin by interrupting the interaction between Moesin and FBXW2. A. MDA-MB-231 cells were treated with increasing doses of AKT inhibitor (0, 2.5 and 5 μM) for 12 h and whole cell protein extracts

were immunoblotted for the indicated proteins. **B.** Whole cell protein extracts of MDA-MB-231 cells expressing either scramble (NS) or AKT-specific two independent shRNAs were immunoblotted for the indicated proteins. **C.** MCF7 cells were transfected with indicated plasmids for 36 h. Transfected cells were treated with 5 μM AKT inhibitor for additional 12 h as indicated. Ubiquitinated proteins were then pulled down with Ni-NTA beads from whole cell lysates. Pulled down fractions and input extracts were immunoblotted for the indicated proteins. **D.** MCF7 cells were transfected with indicated plasmids for 36 h. Transfected cells were treated with 5 μM of AKT inhibitor for additional 12 h and whole cell lysates were then immunoprecipitated with anti-FLAG antibody. Immunoprecipitates and input protein extracts were immunoblotted for the indicated proteins. **E.** NS and AKT depleted MCF7 cells were transfected with myc-FBXW2 plasmid for 36 h. Transfected cells were treated with 5 μM MG132 for additional 6 h and whole cell lysates were then immunoprecipitated with anti-myc antibody. Immunoprecipitates and input protein extracts were immunoblotted for the indicated proteins. **F.** NS and AKT depleted MCF7 cells were transfected with the indicated plasmids for 36 h. Transfected cells were treated with 5 μM MG132 for additional 6 h and whole cell lysates were then pulled down with Ni-NTA beads. Pulled down fractions and input protein extracts were immunoblotted for the indicated proteins. **G.** Quantification of IHC data in Fig. 6H.

Supplementary Figure S1

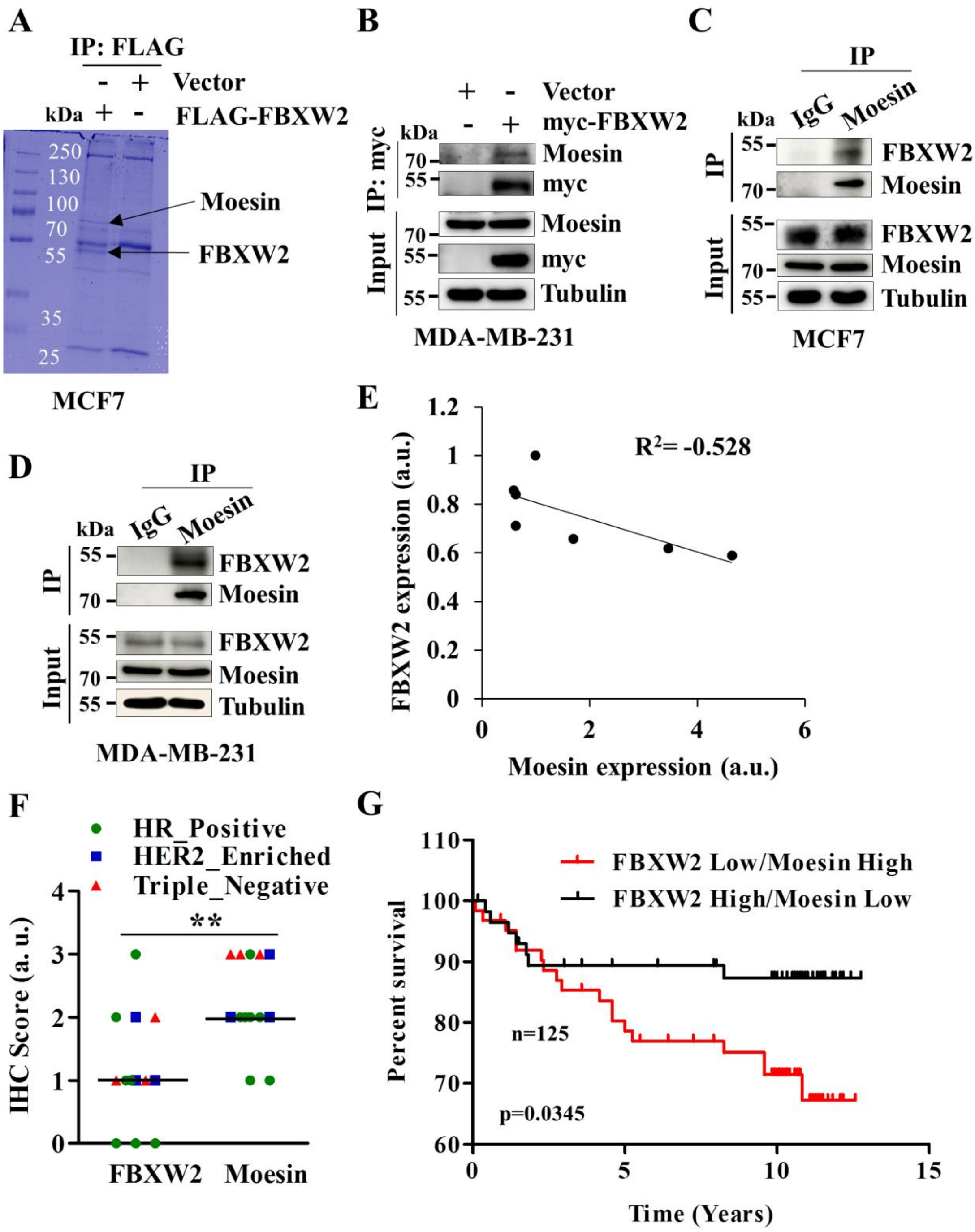

Supplementary Figure S2

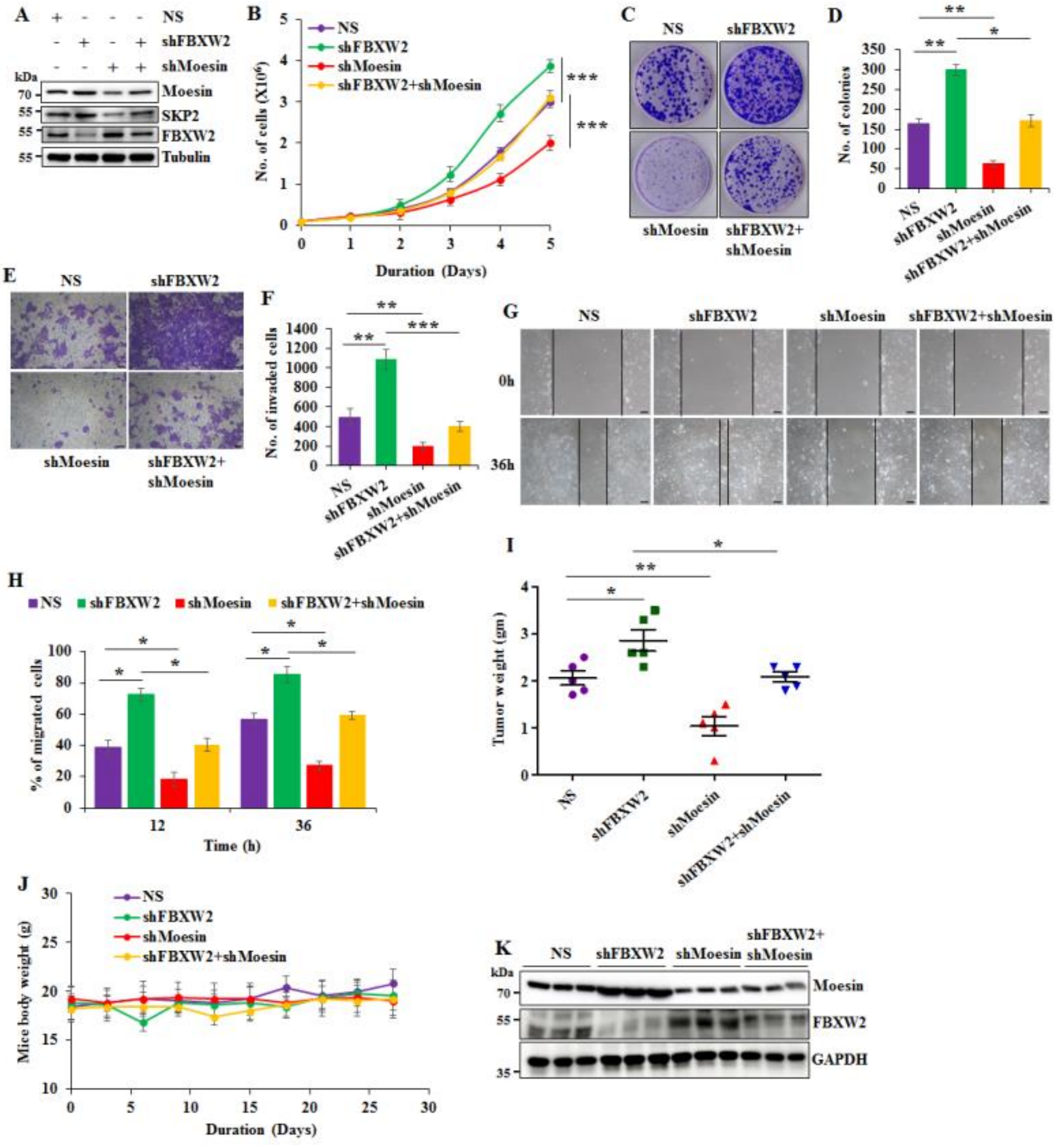

Supplementary Figure S3

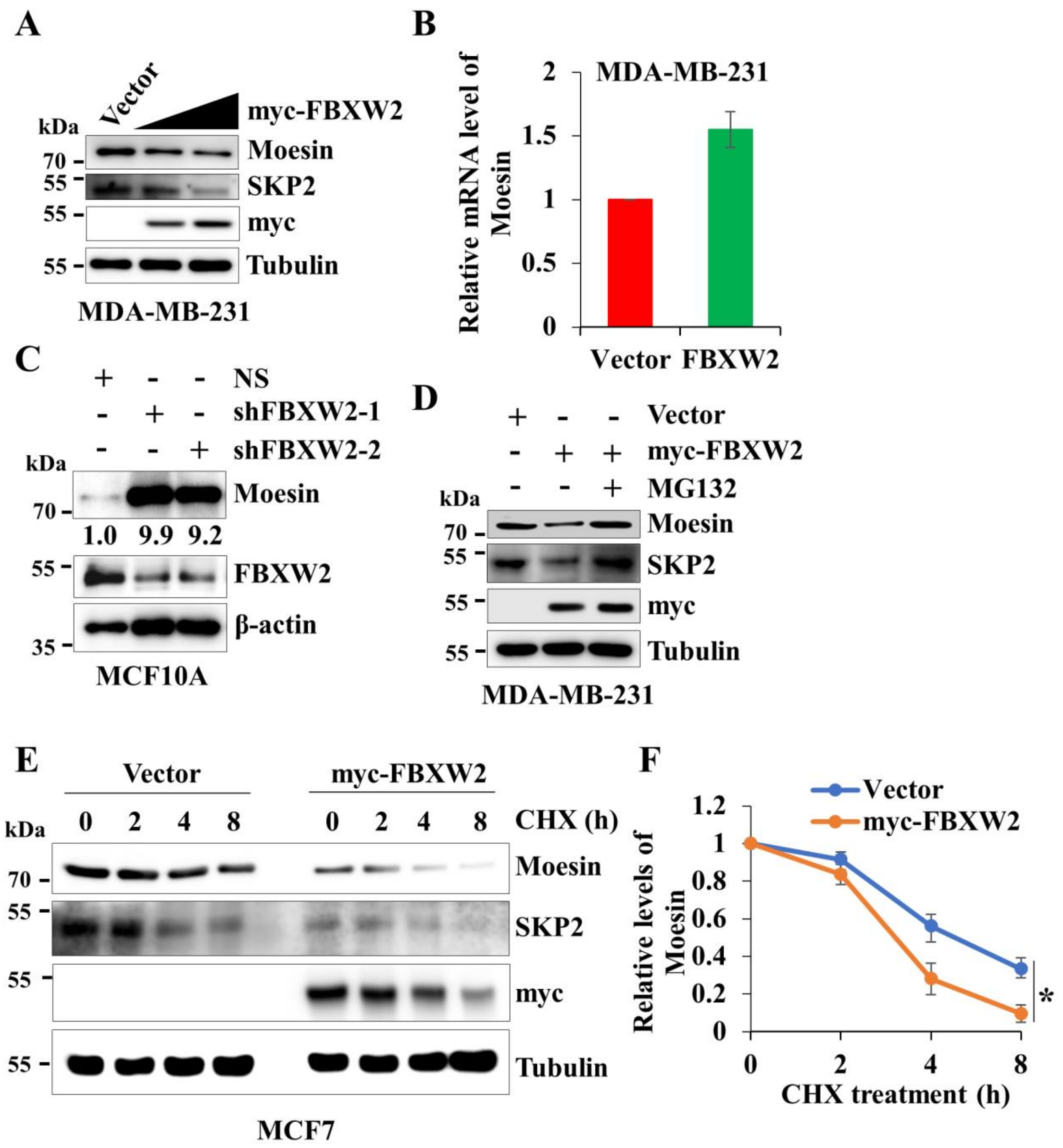

Supplementary Figure S4

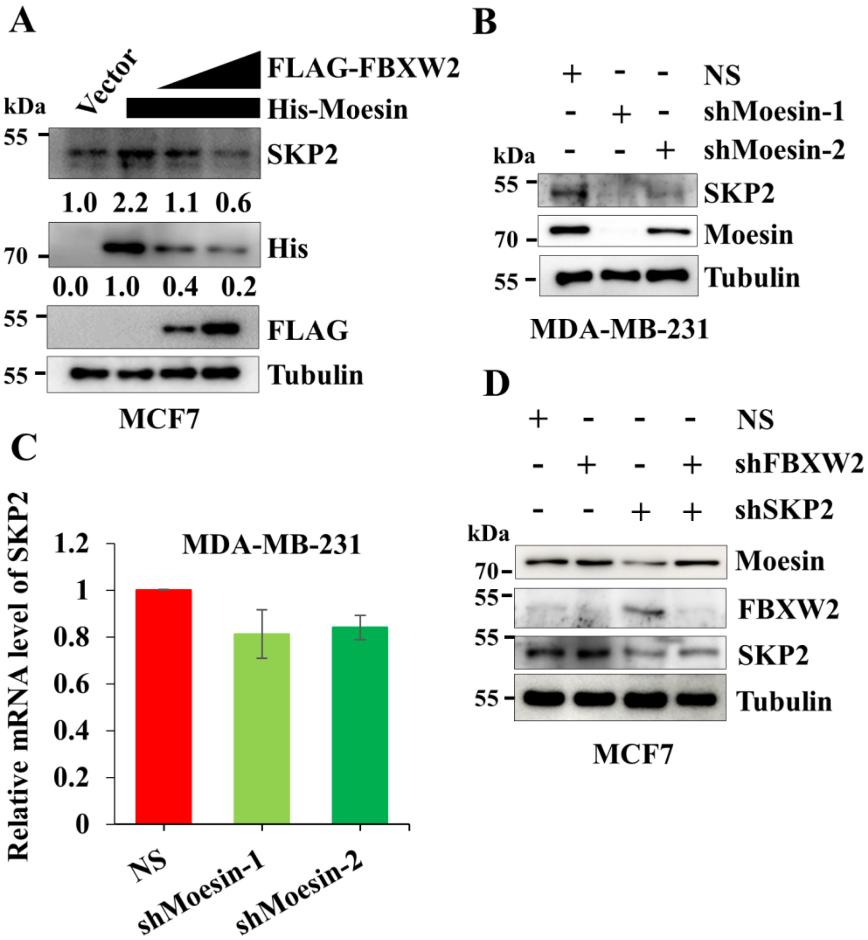

Supplementary Figure S5

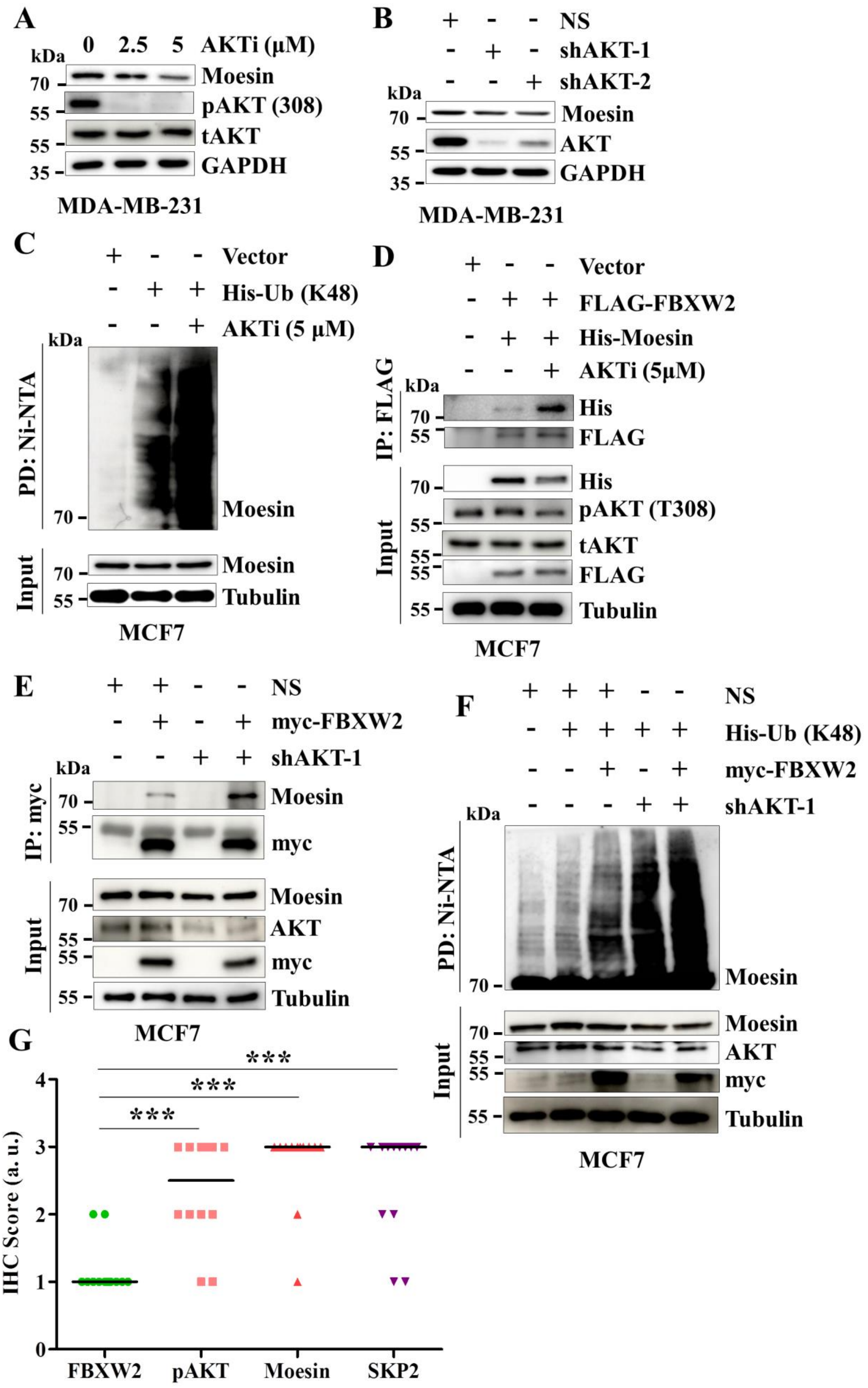

Original data files

Figure 1

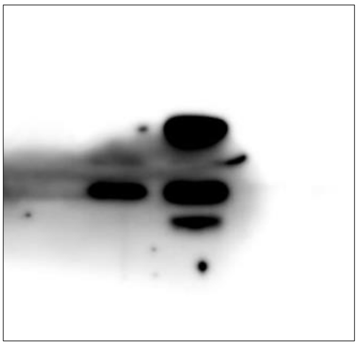

Figure 1A-IP-His, IB-FLAG

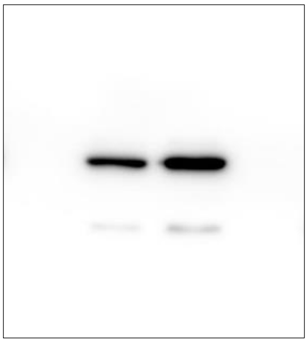

Figure 1A-IP-His, IB-His

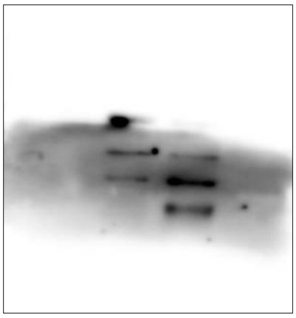

Figure 1A-Input, IB-FLAG

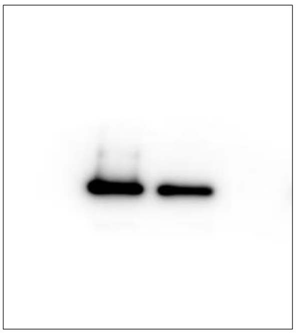

Figure 1A-Input, IB-His

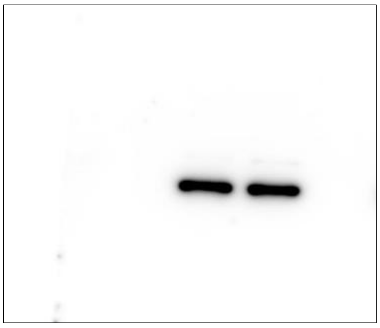

Figure 1A-Input, IB-Tubulin

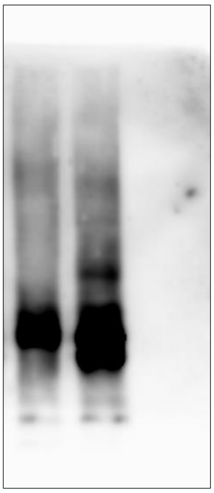

Fig. 1B-IP-FBXW2, IB-Moesin

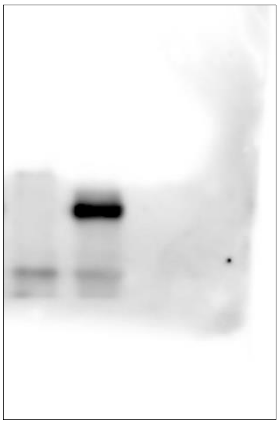

Fig. 1B-IP-FBXW2, IB-FBXW2

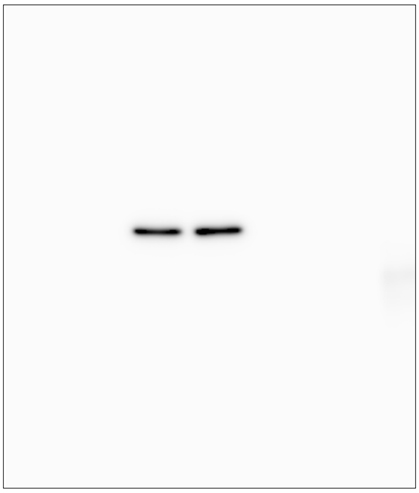

Fig. 1B-Input, IB-Moesin

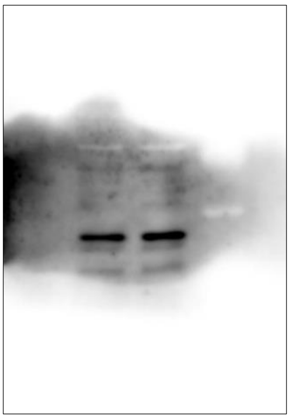

Figure 1B-Input, IB-FBXW2

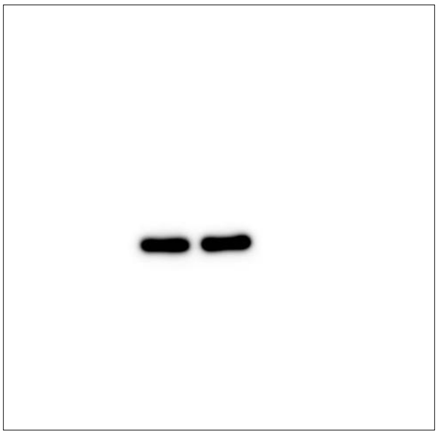

Figure 1B-Input, IB-Tubulin

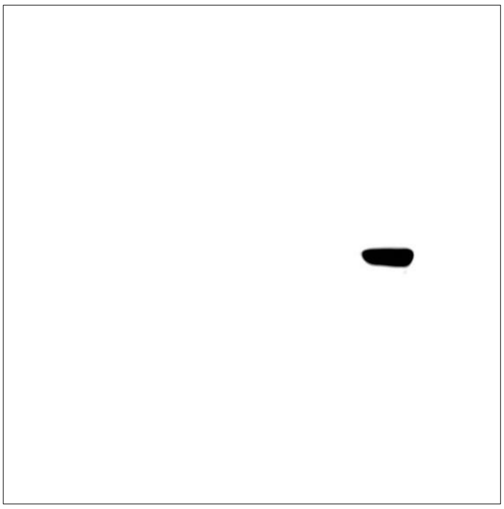

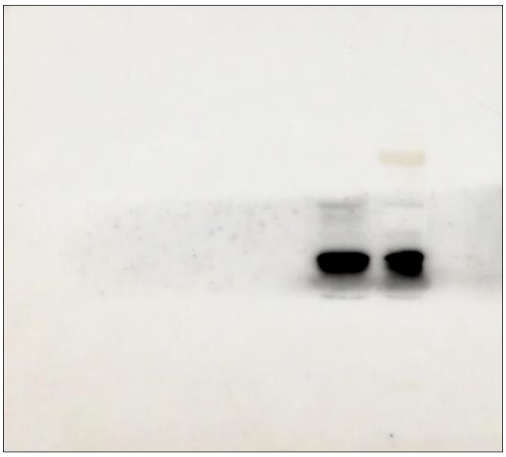

Figure 1C-PD-Ni-NTA, IB-GST

Figure 1C-PD-Ni-NTA, IB-His

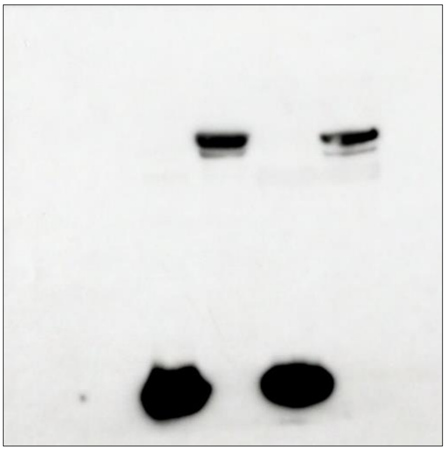

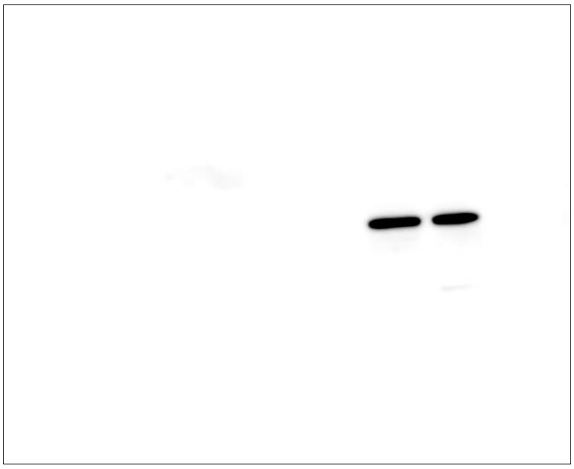

Figure 1C-Input, IB-GST

Figure 1C-Input, IB-His

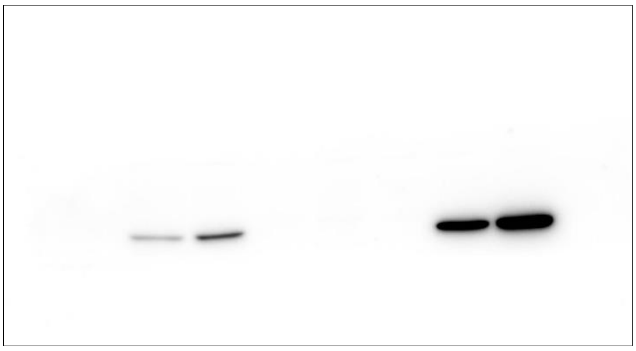

Figure 1D, IB-Moesin

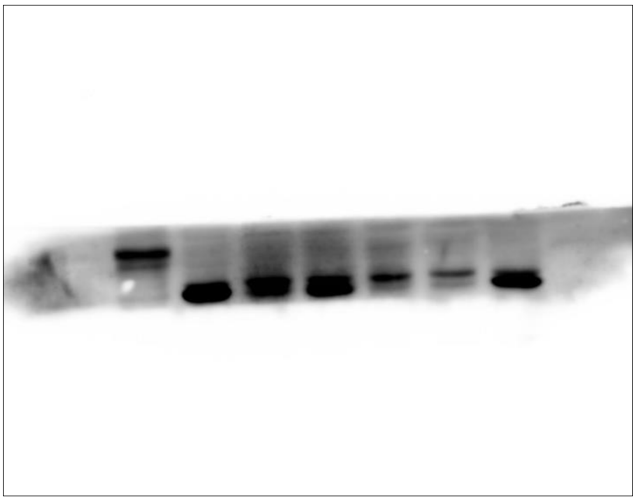

Figure 1D, IB-FBXW2

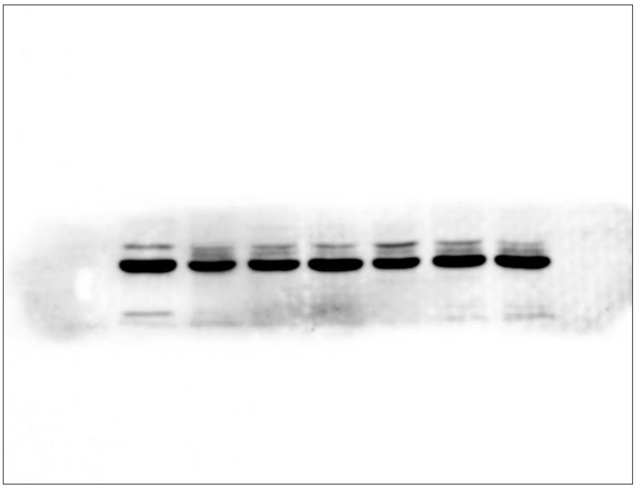

Figure 1D, IB-β-Actin

Figure 3

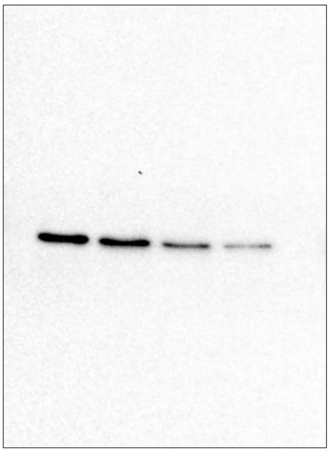

Figure 3A, IB-Moesin

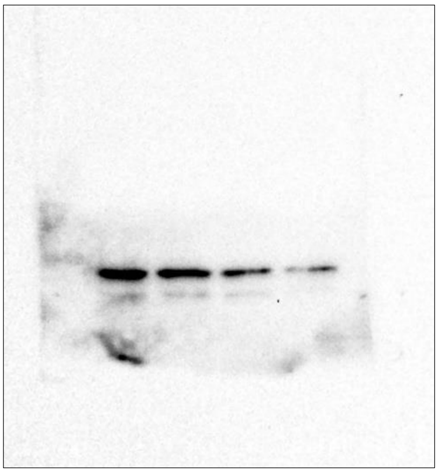

Figure 3A, IB-SKP2

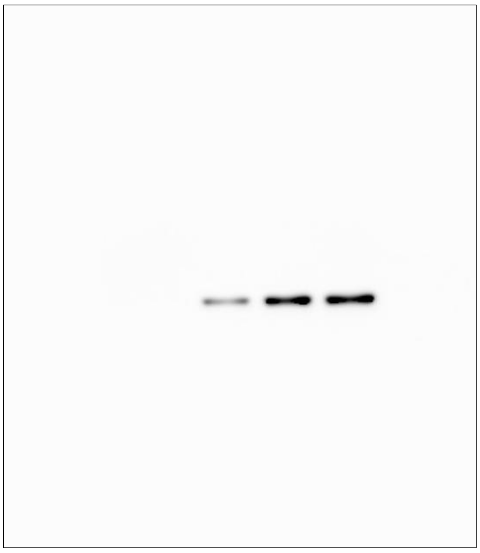

Figure 3A, IB-FLAG

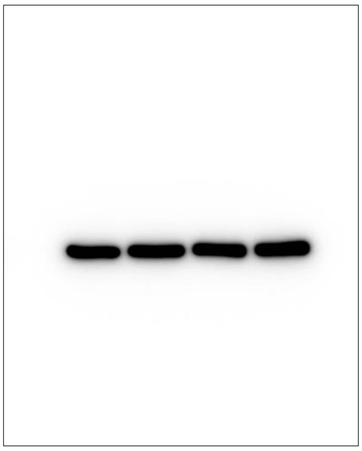

Figure 3A, IB-Tubulin

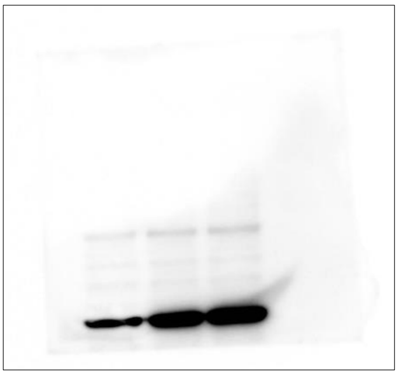

Figure 3C, IB-Moesin

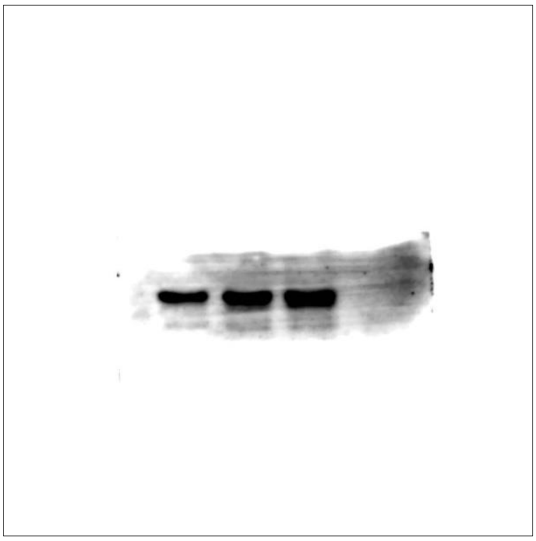

Figure 3C, IB-SKP2

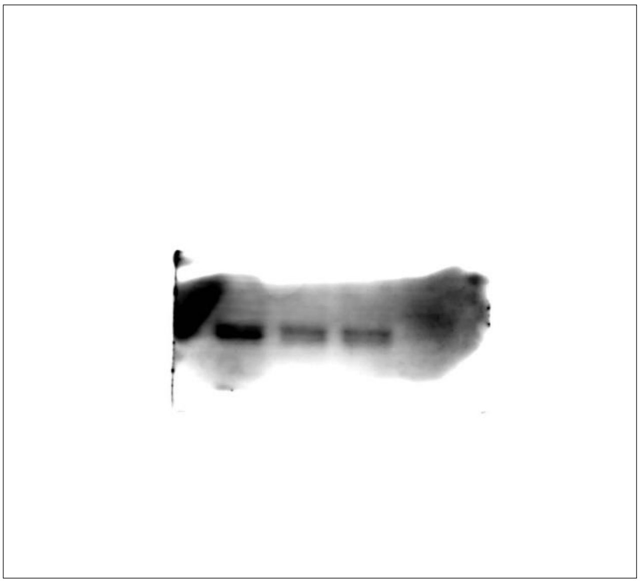

Figure 3C, IB-FBXW2

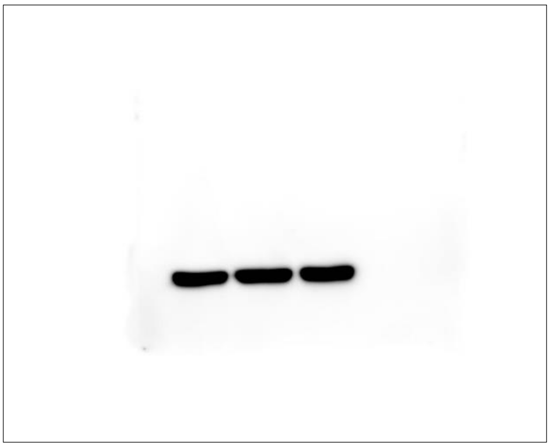

Figure 3C, IB-Tubulin

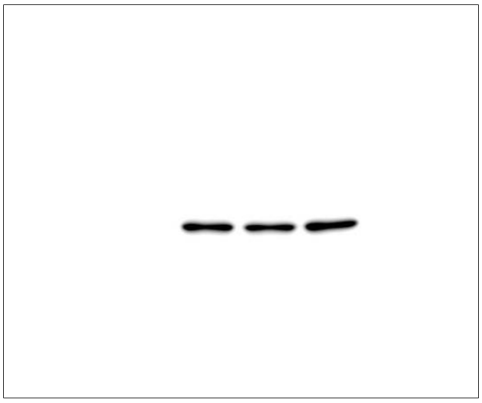

Figure 3E, IB-Moesin

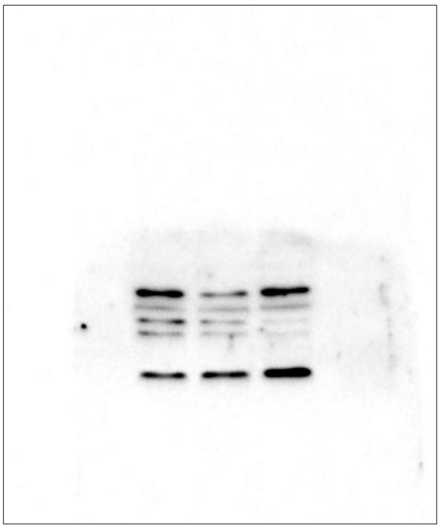

Figure 3E, IB-SKP2

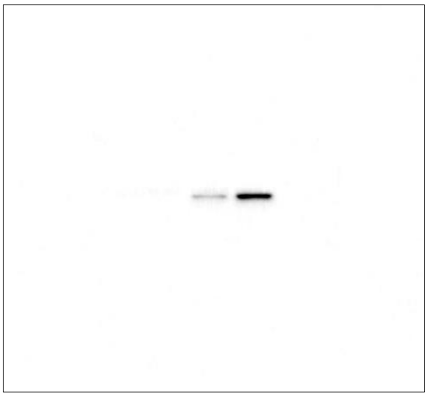

Figure 3E, IB-FLAG

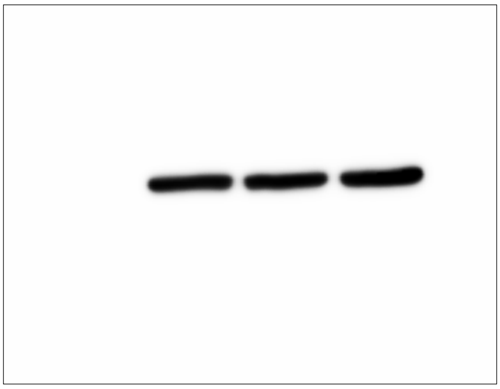

Figure 3E, IB-Tubulin

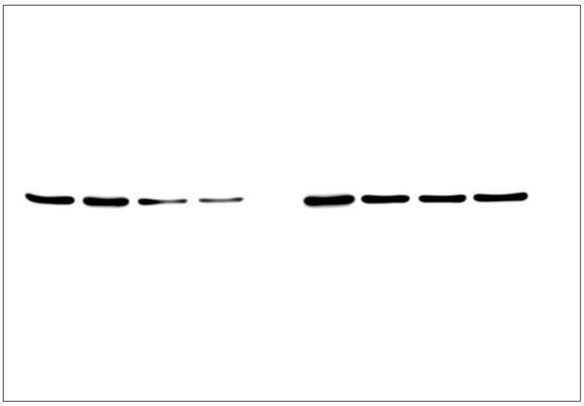

Figure 3F, IB-Moesin

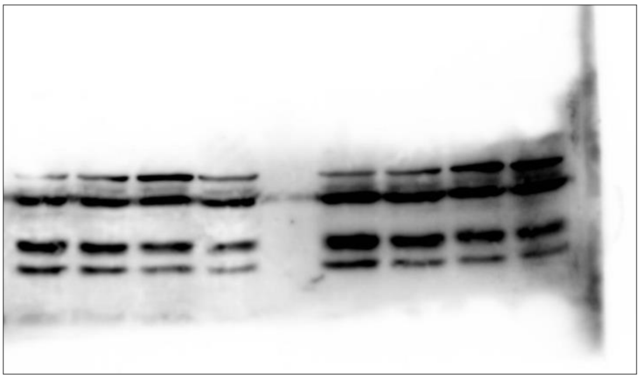

Figure 3F, IB-SKP2

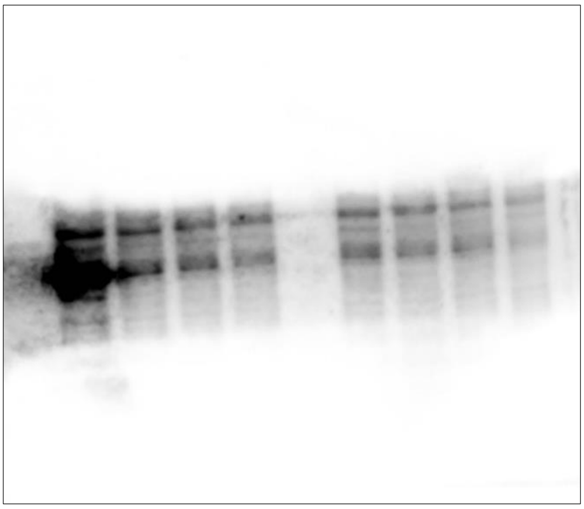

Figure 3F, IB-FBXW2

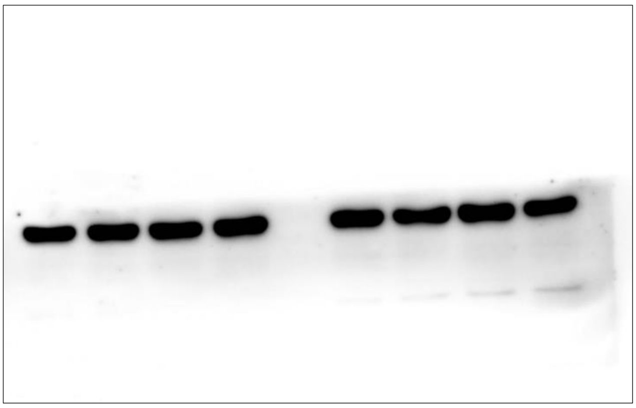

Figure 3F, IB-Tubulin

Figure 4

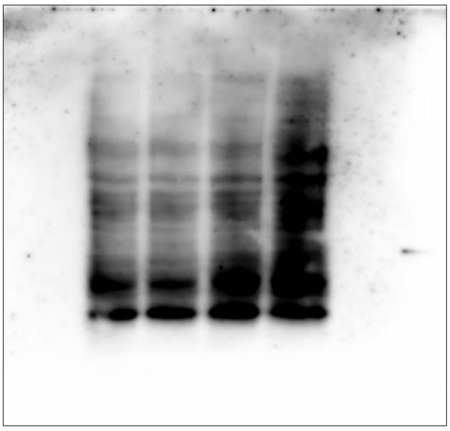

Figure 4A-PD-Ni-NTA, IB-Moesin

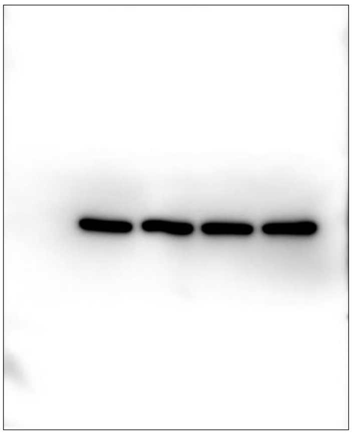

Figure 4A-Input, IB-Moesin

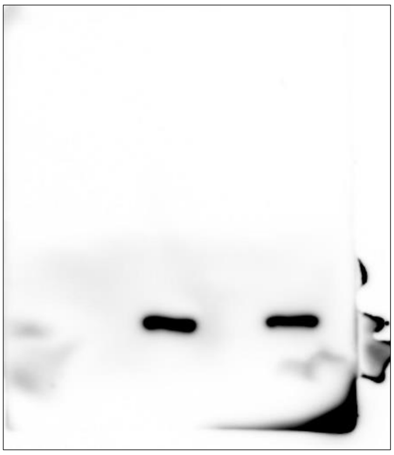

Figure 4A-Input, IB-FLAG

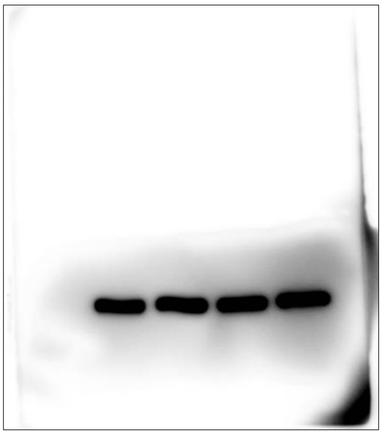

Figure 4A-Input, IB-Tubulin

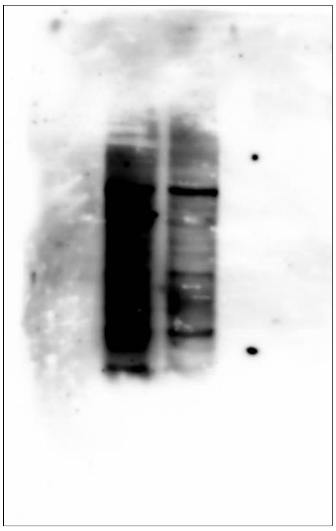

Figure 4B-PD-Ni-NTA, IB-Moesin

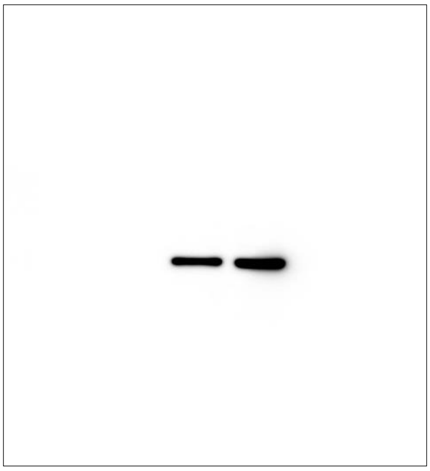

Figure 4B-Input, IB-Moesin

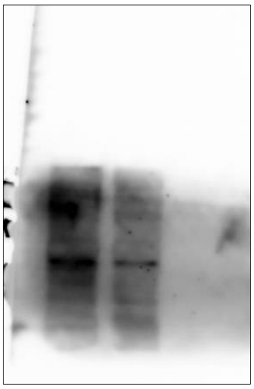

Figure 4B-Input, IB-FBXW2

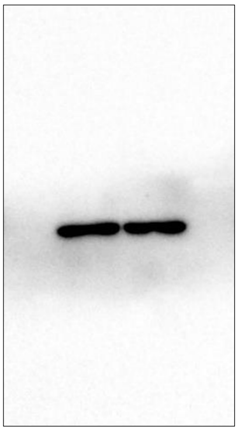

Figure 4B-Input, IB-Tubulin

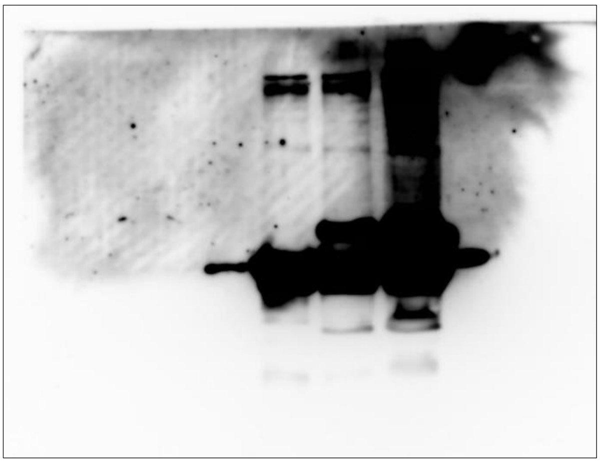

Figure 4C, IB-GST

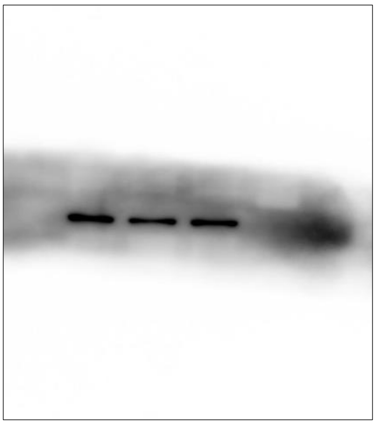

Figure 4D, IB-Moesin

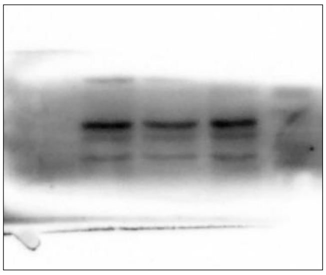

Figure 4D, IB-SKP2

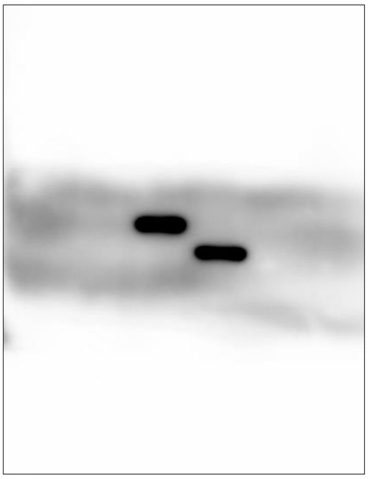

Figure 4D, IB-FLAG

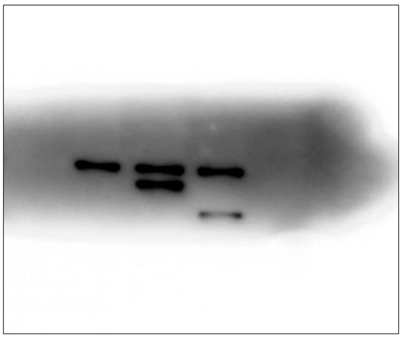

Figure 4D, IB-Tubulin

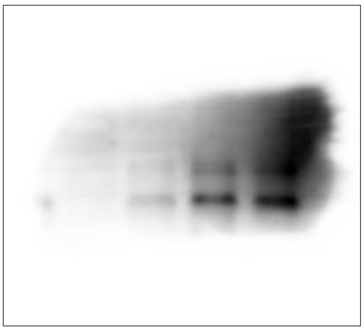

Fig. 4E-IP-FLAG, IB-Moesin

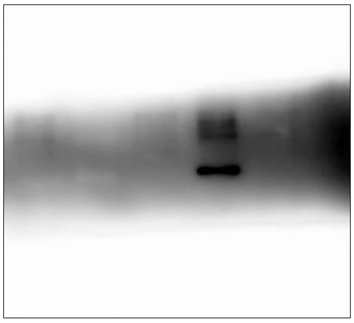

Fig. 4E-IP-FLAG, IB-Cullin1

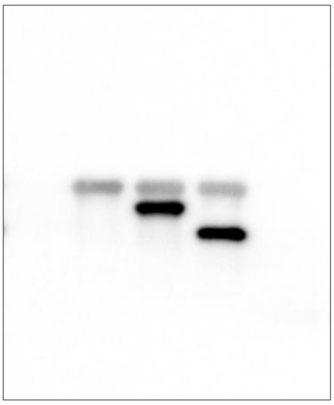

Fig. 4E-IP-FLAG, IB-FLAG

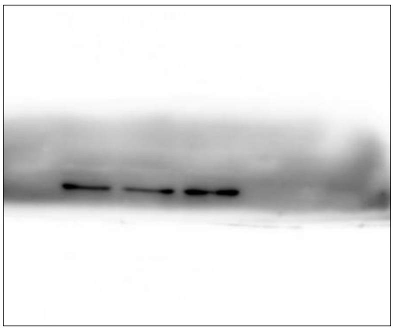

Figure 4E-Input, IB-Moesin

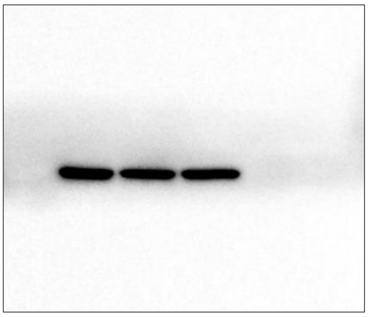

Fig. 4E-Input, IB-Cullin1

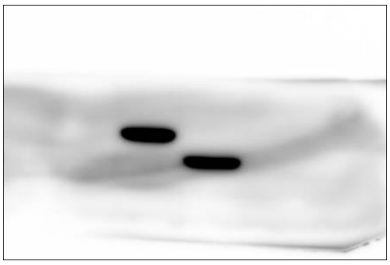

Fig. 4E-Input, IB-FLAG

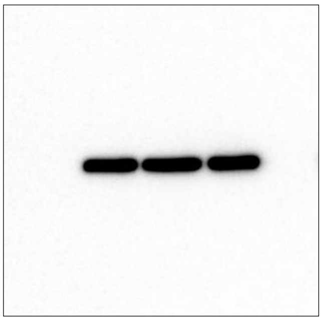

Fig. 4E-Input, IB-Tubulin

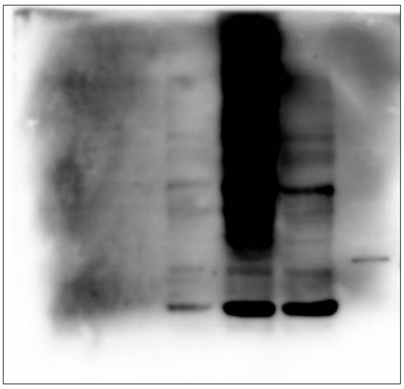

Figure 4F-PD-Ni-NTA, IB-Moesin

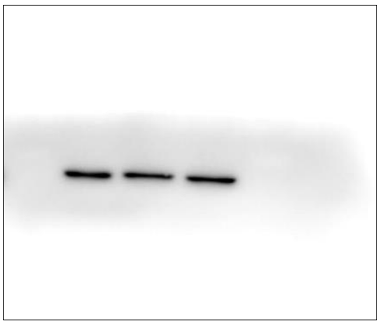

Figure 4F-Input, IB-Moesin

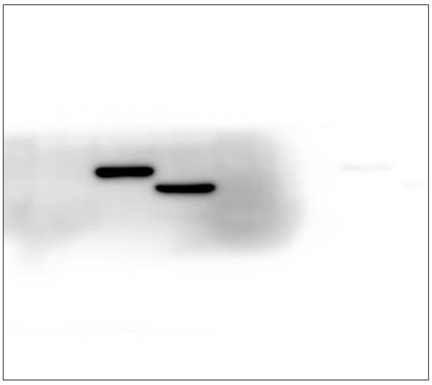

Figure 4F-Input, IB-FLAG

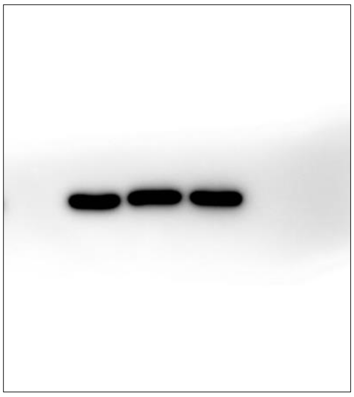

Figure 4F-Input, IB-Tubulin

Figure 5

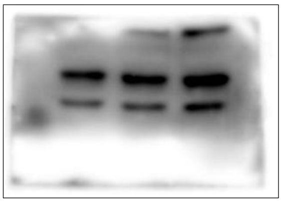

Figure 5A, IB-SKP2

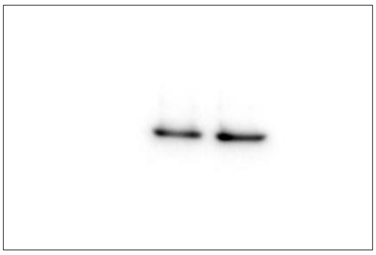

Figure 5A, IB-His

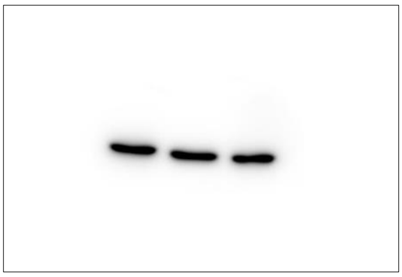

Figure 5A, IB-β-Actin

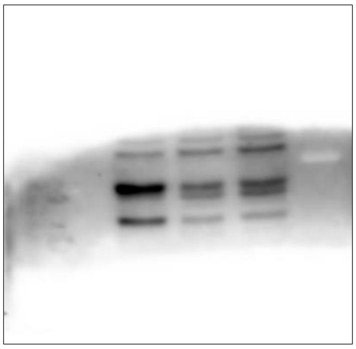

Figure 5B, IB-SKP2

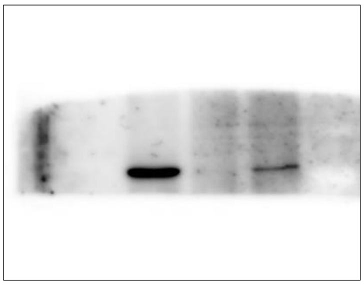

Figure 5B, IB-Moesin

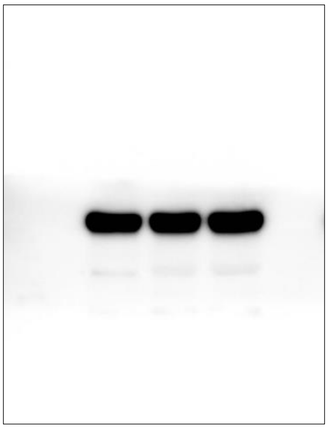

Figure 5B, IB-Tubulin

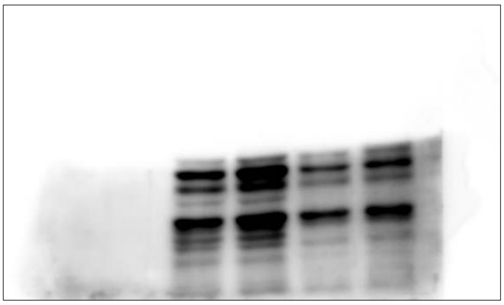

Figure 5D, IB-SKP2

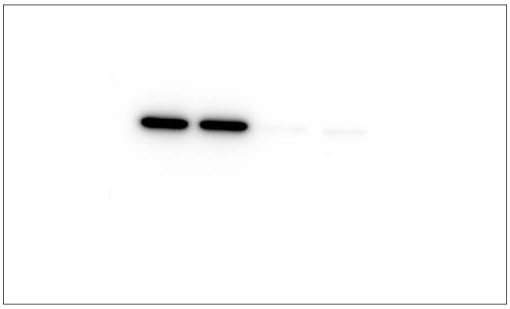

Figure 5D, IB-Moesin

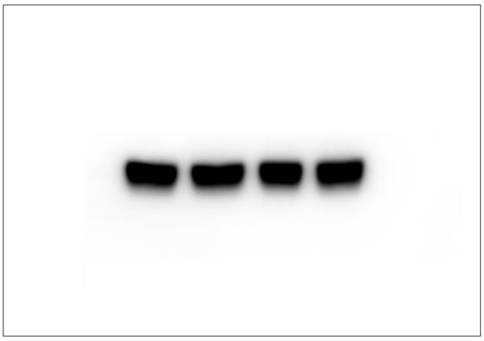

Figure 5D, IB-Tubulin

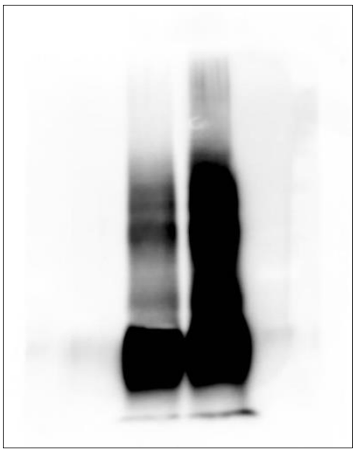

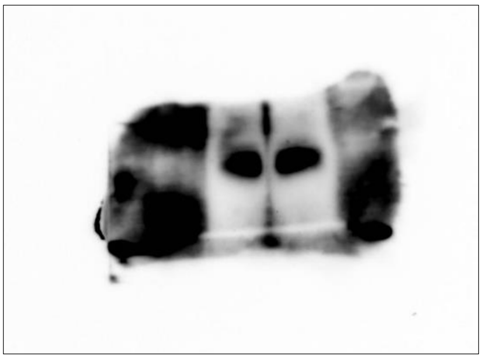

Figure 5E-IP-SKP2, IB-K48 Figure 5E-IP-SKP2, IB-SKP2

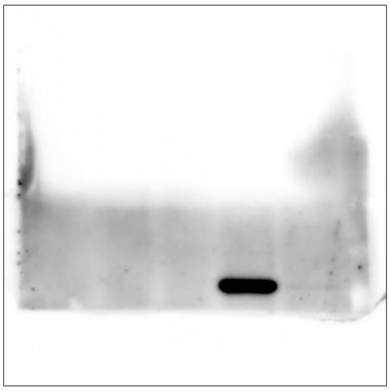

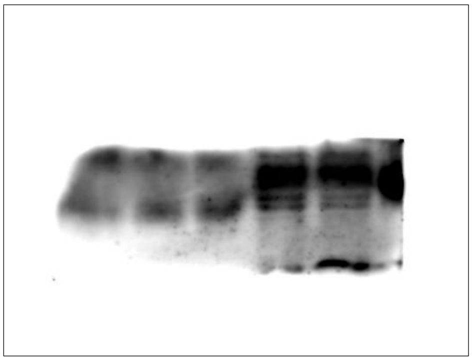

Figure 5E-Input, IB-Moesin Figure 5E-Input, IB-SKP2

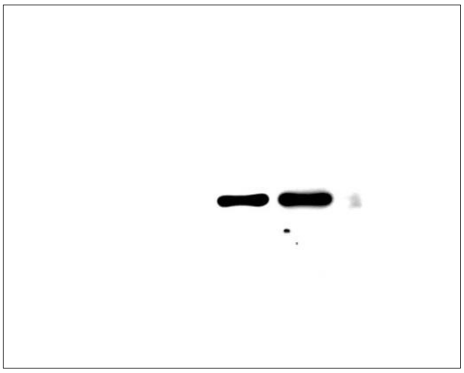

Figure 5E-Input, IB-Tubulin

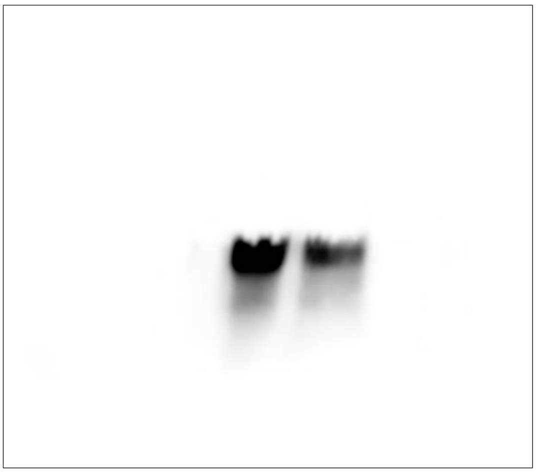

Figure 5F-IP-FBXW2, IB-SKP2

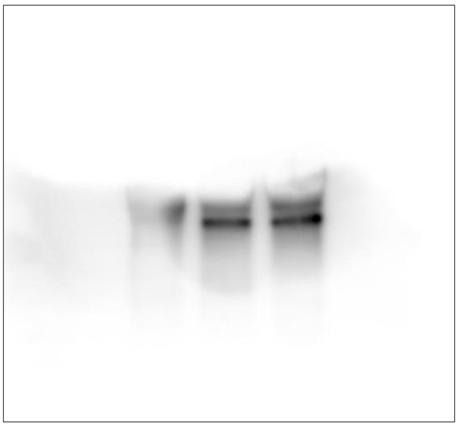

Figure 5F-IP-FBXW2, IB-FBXW2

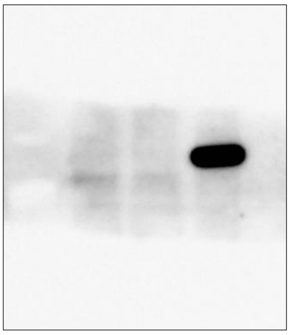

Fig. 5F-IP-FBXW2, IB-His

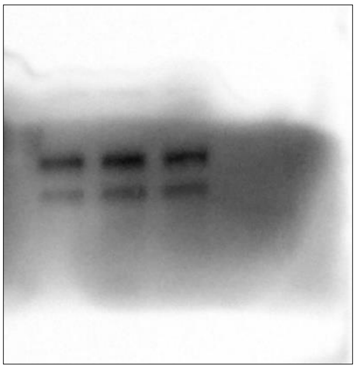

Fig. 5F-Input, IB-SKP2

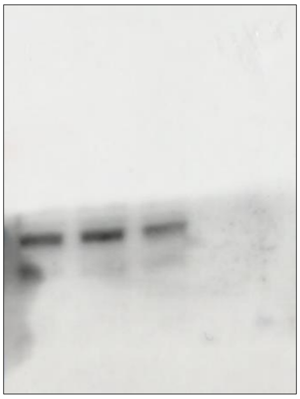

Fig. 5F-Input, IB-FBXW2

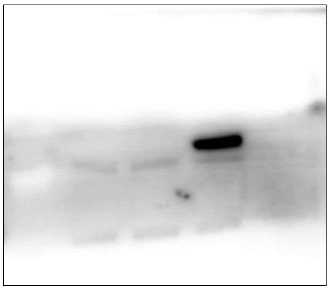

Figure 5F-Input, IB-His

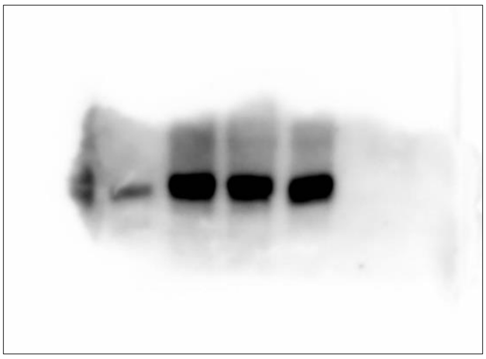

Figure 5F-Input, IB-Tubulin

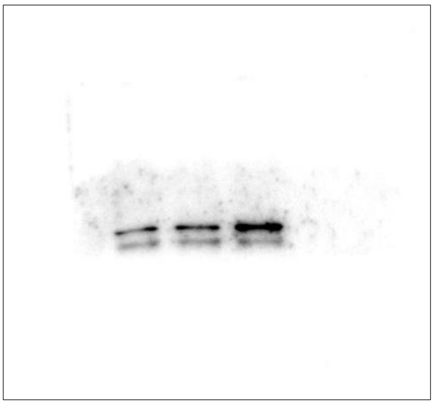

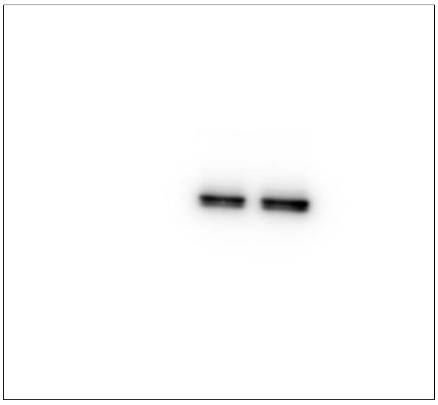

Figure 5G, IB-Moesin

Figure 5G, IB-FLAG

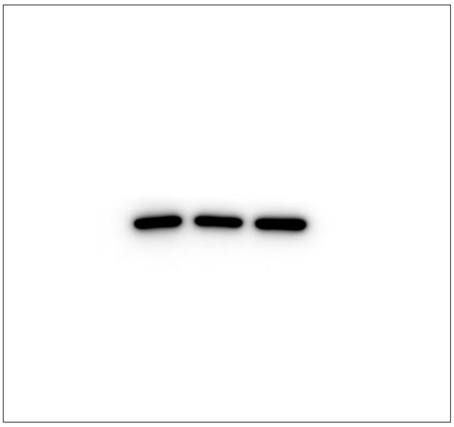

Figure 5G, IB-Tubulin

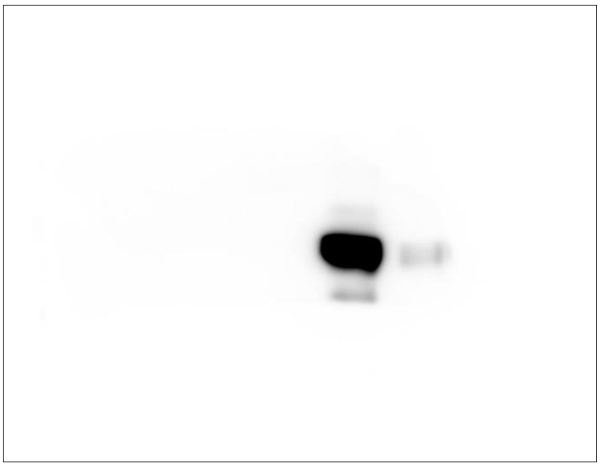

Figure 5I-PD-Ni-NTA, IB-FLAG

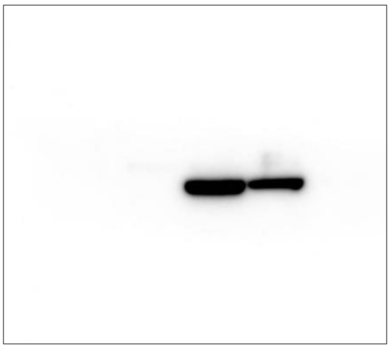

Figure 5I-PD-Ni-NTA, IB-His

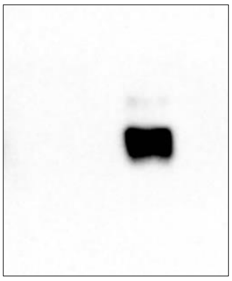

Figure 5I-Input, IB-FLAG

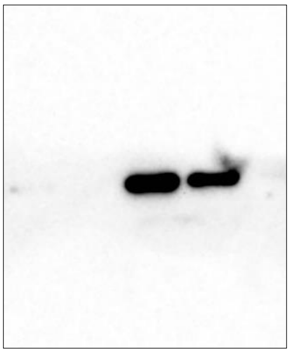

Figure 5I-Input, IB-His

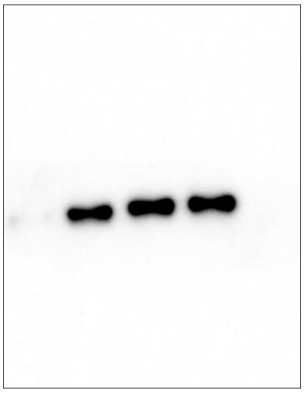

Figure 5I-Input, IB-Tubulin

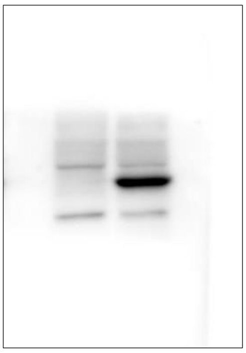
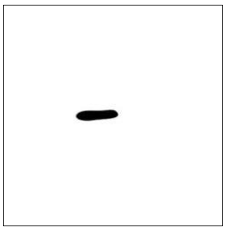
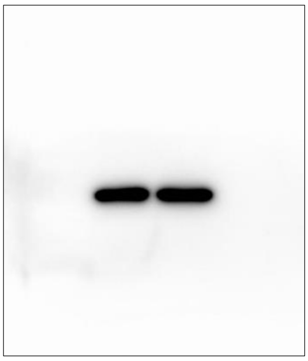

Fig. 5J-IP-SKP2, IB-Moesin Fig. 5J-IP-SKP2, IB-SKP2 Fig. 5J-Input, IB-Moesin

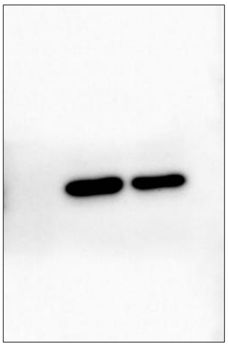
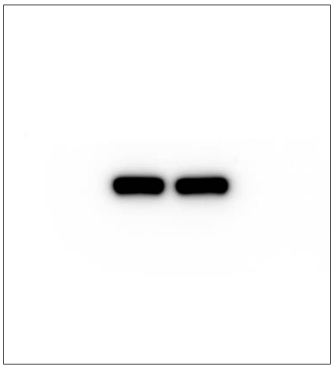

Figure 5J-Input, IB-SKP2 Figure 5J-Input, IB-Tubulin

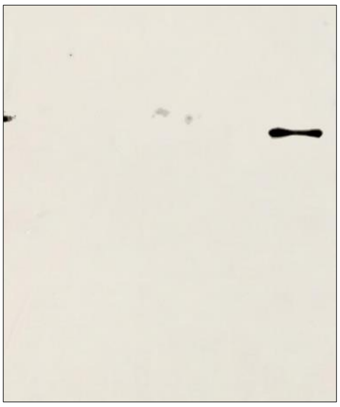

Figure 5K-IP-FLAG, IB-GST

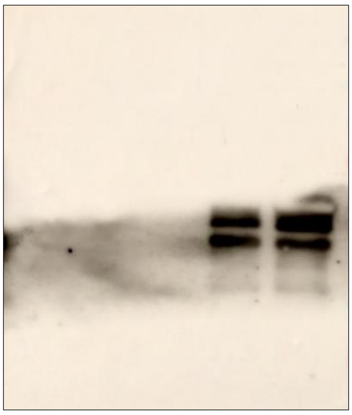

Figure 5K-IP-FLAG, IB-FLAG

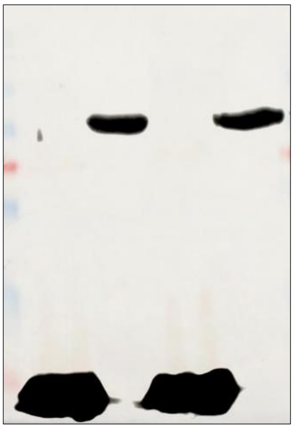

Figure 5K-Input, IB-GST

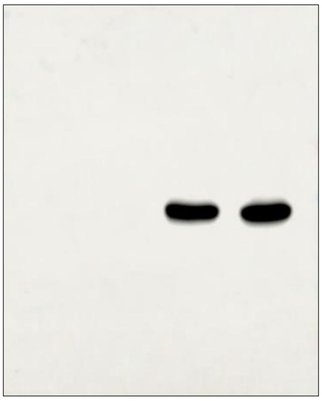

Figure 5K-Input, IB-FLAG

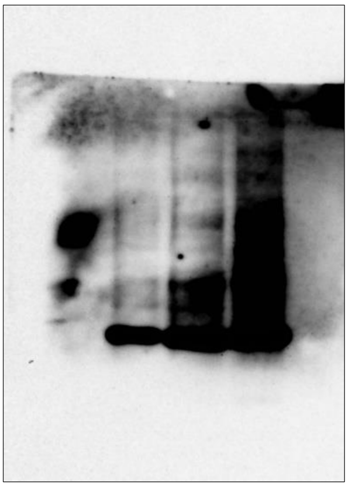

Figure 5L-PD-Ni-NTA, IB-Moesin

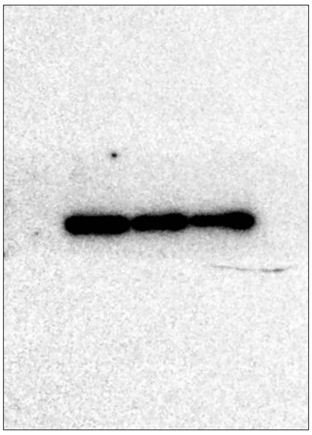

Figure 5L-Input, IB-Moesin

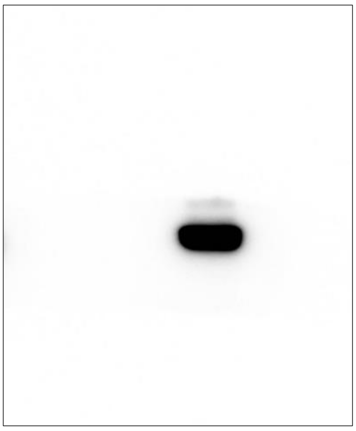

Figure 5L-Input, IB-FLAG

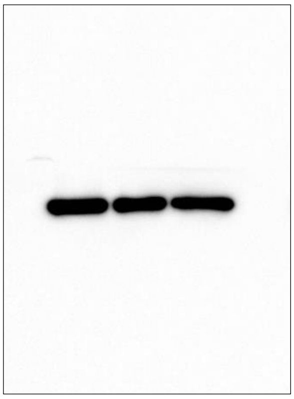

Figure 5L-Input, IB-Tubulin

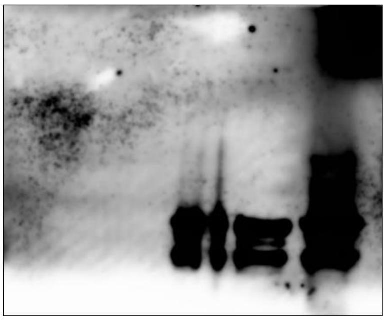

Figure 5M-IP-FLAG, IB-GST

Figure 6

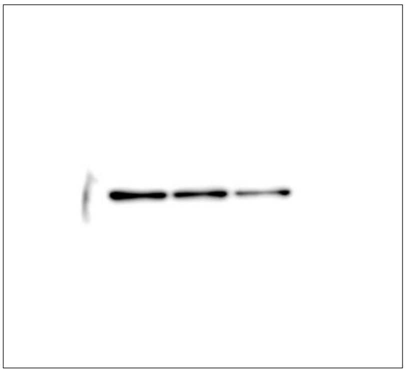

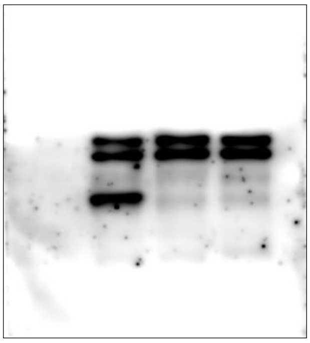

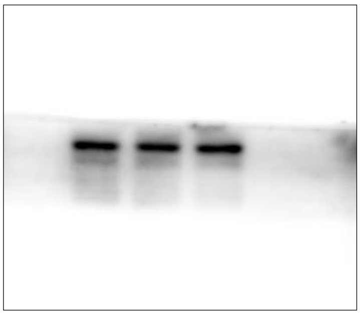

Figure 6A, IB-Moesin

Figure 6A, IB-pAKT (T308)

Figure 6A, IB-tAKT

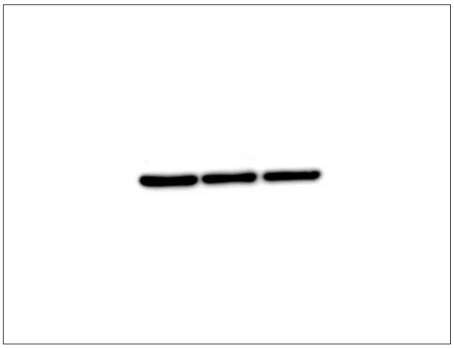

Figure 6A, IB-Tubulin

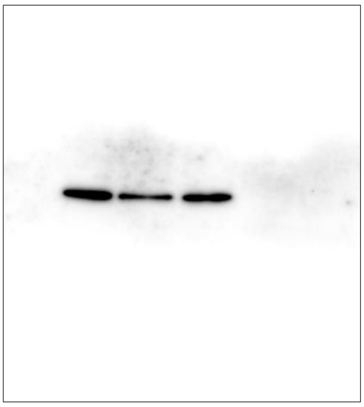

Fig. 6B, IB-Moesin

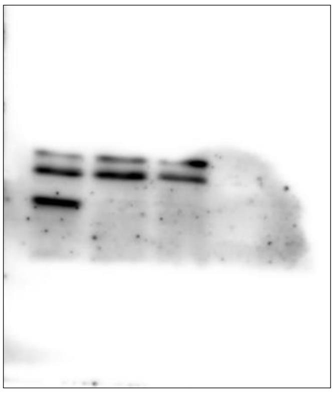

Fig. 6B, IB-pAKT(T308)

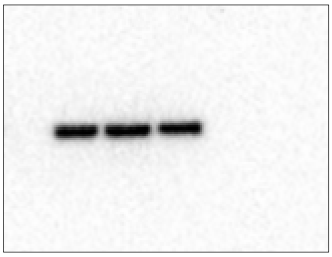

Fig. 6B, IB-tAKT

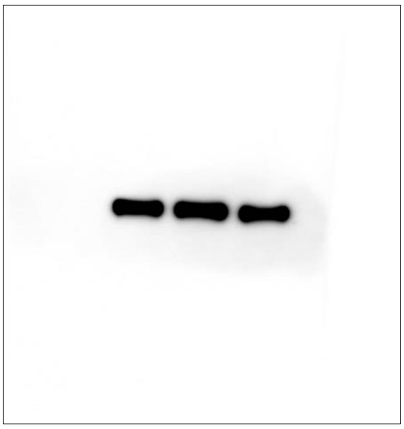

Figure 6B, IB-Tubulin

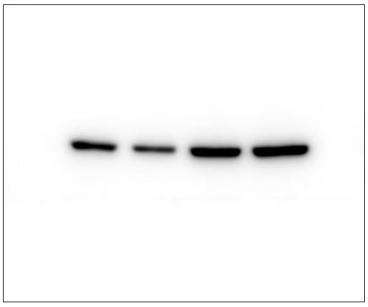

Fig 6C, IB-Moesin

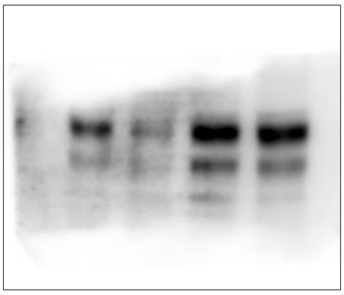

Figure 6C, IB-SKP2

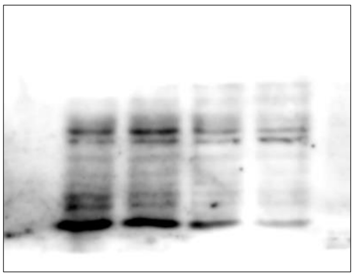

Figure 6C, IB-FBXW2

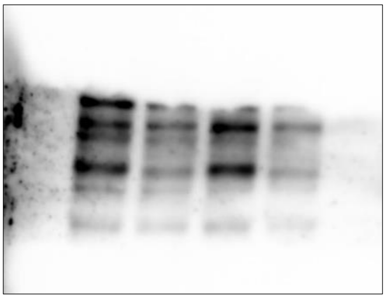

Figure 6C, IB-pAKT (T308)

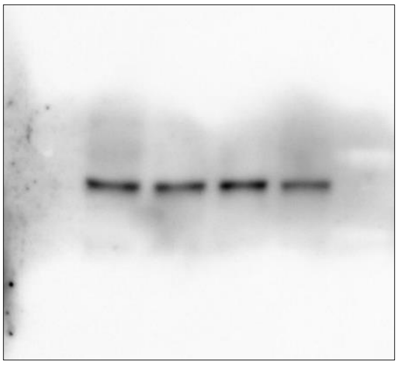

Figure 6C, IB-tAKT

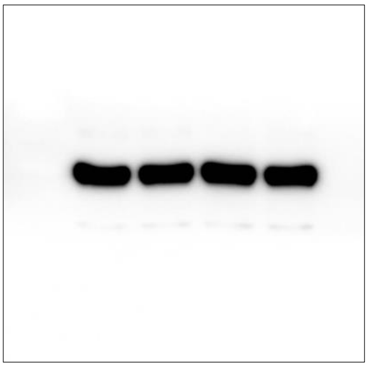

Figure 6C, IB-Tubulin

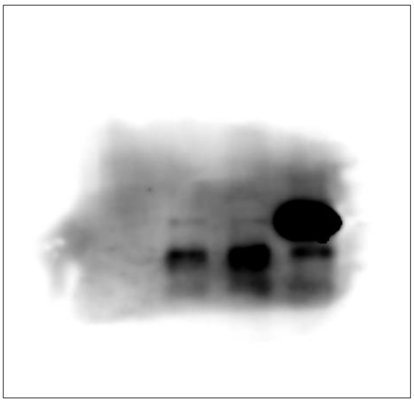

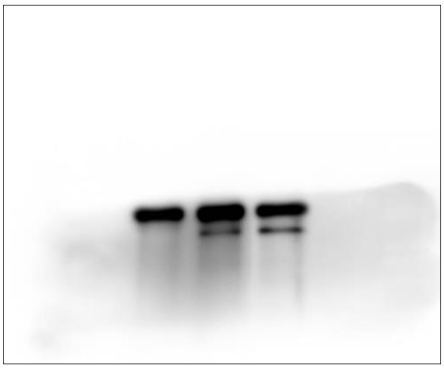

Figure 6D-IP-FLAG, IB-Moesin Figure 6D-IP-FLAG, IB-FLAG

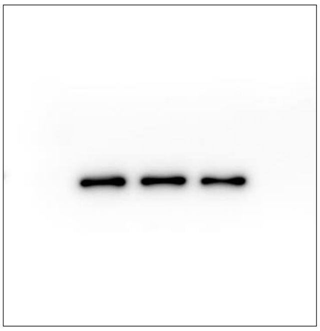

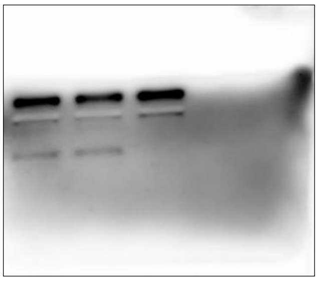

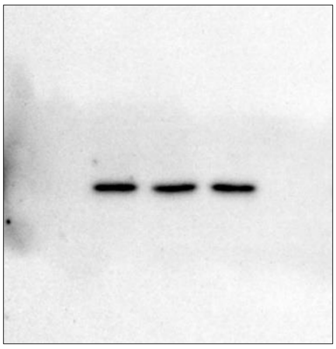

Fig 6D-Input, IB-Moesin Fig 6D-Input, IB-pAKT (T308) Fig 6D-Input, IB-tAKT

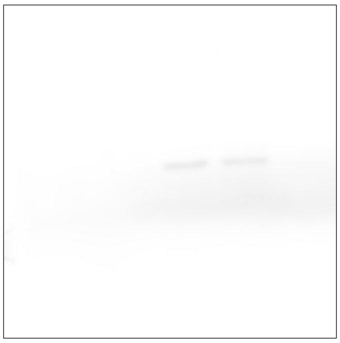

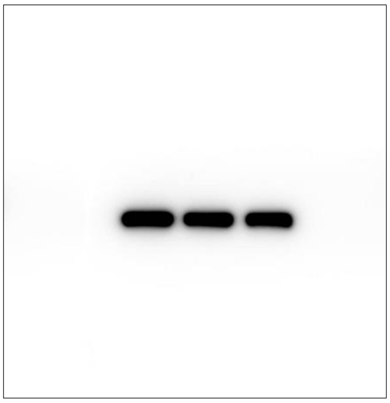

Figure 6D-Input, IB-FLAG Figure 6D-Input, IB-Tubulin

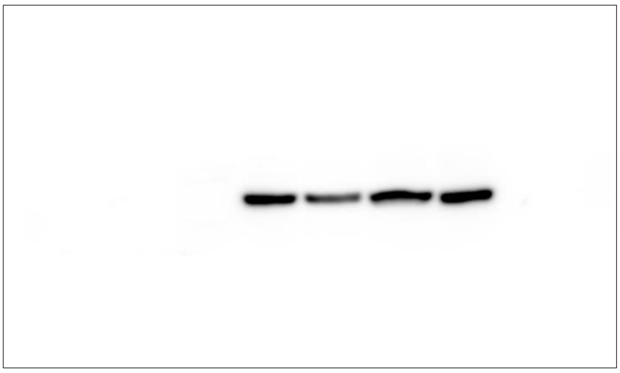

Figure 6E, IB-His

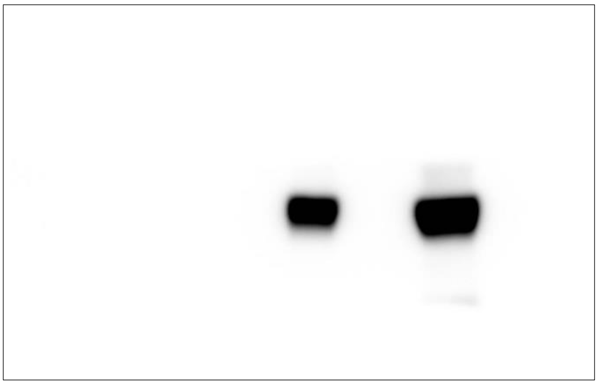

Figure 6E, IB-myc

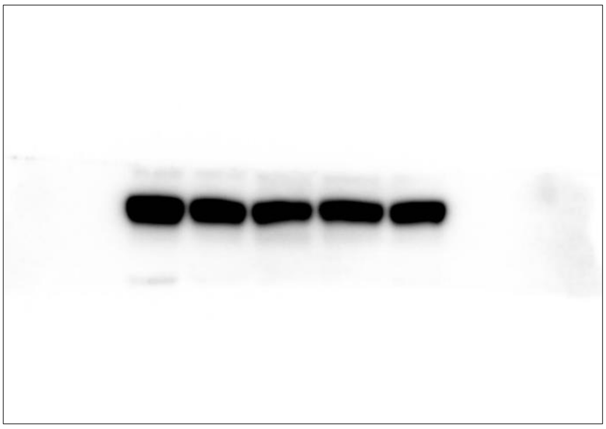

Figure 6E, IB-Tubulin

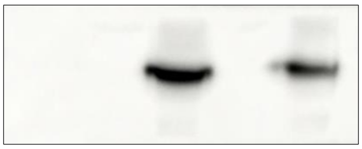

Figure 6F-PD-Ni-NTA, IB-myc

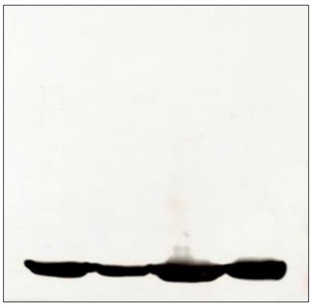

Figure 6F-PD-Ni-NTA, IB-His

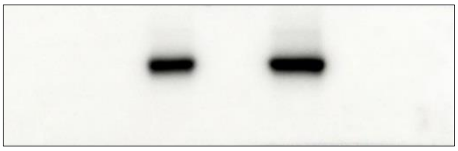

Figure 6F-Input, IB-myc

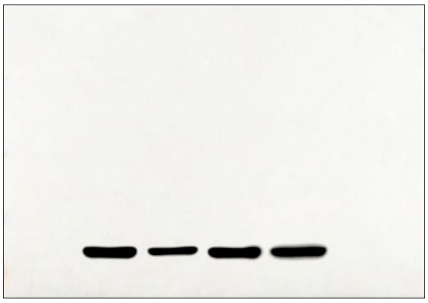

Figure 6F-Input, IB-His

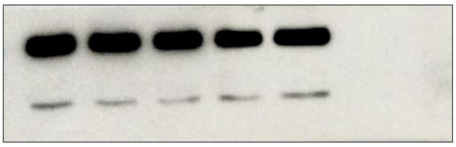

Figure 6F-Input, IB-Tubulin

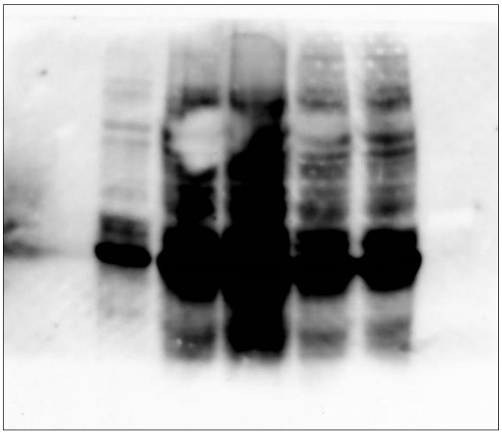

Figure 6G-PD-Ni-NTA, IB-FLAG

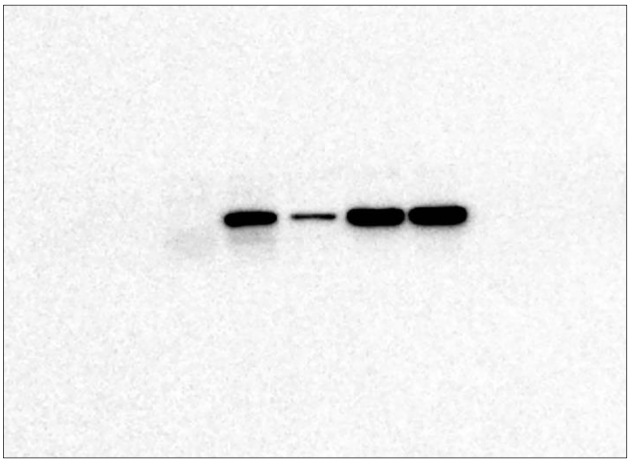

Figure 6G-Input, IB-His

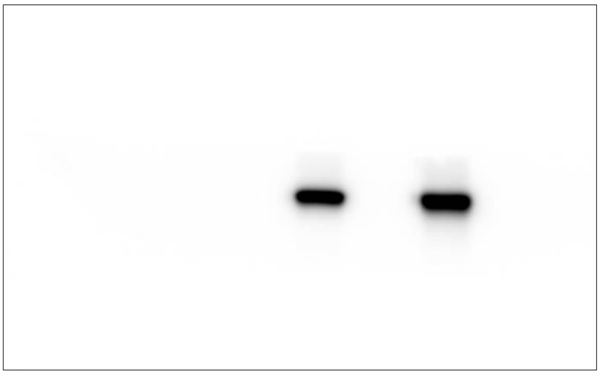

Figure 6G-Input, IB-myc

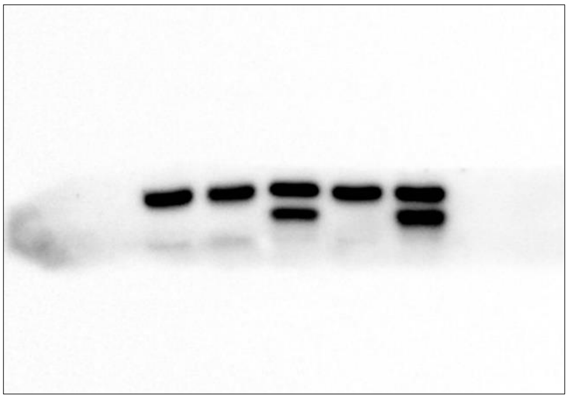

Figure 6G-Input, IB-Tubulin

Supplementary Figure S1

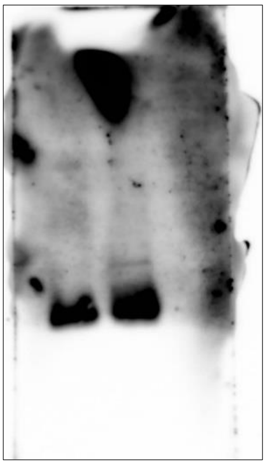

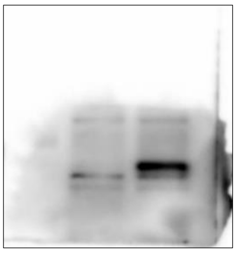

Supp. Figure S1B-IP-myc, IB-Moesin

Supp. Figure S1B-IP-myc, IB-myc

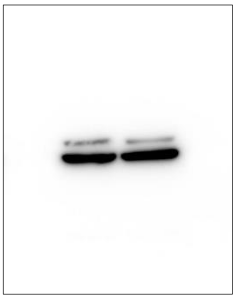

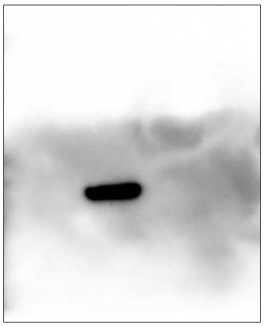

Supp. Fig. S1B-Input, IB-Moesin

Supp. Fig. S1B-Input, IB-myc

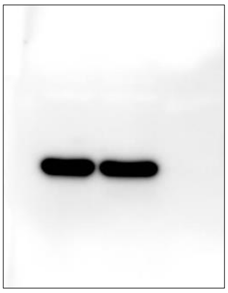

Supp. Fig. S1B-Input, IB-Tubulin

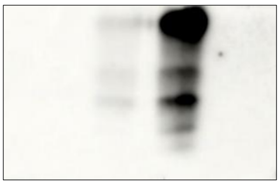

Supp. Figure S1C-IP-Moesin, IB-FBXW2

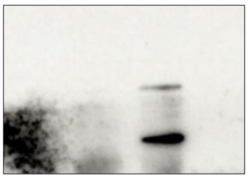

Supp. Figure S1C-IP-Moesin, IB-Moesin

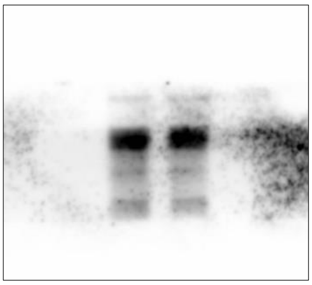

Supp. Figure S1C-Input, IB-FBXW2

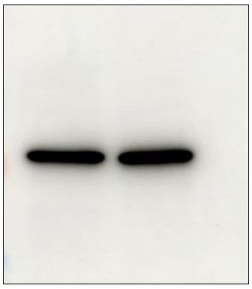

Supp. Figure S1C-Input, IB-Moesin

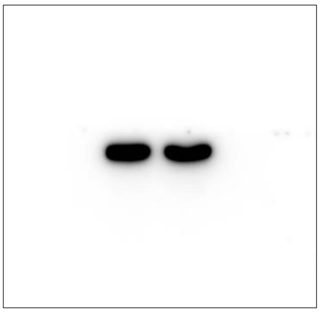

Supp. Figure S1C-Input, IB-Tubulin

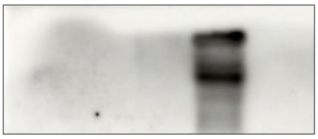

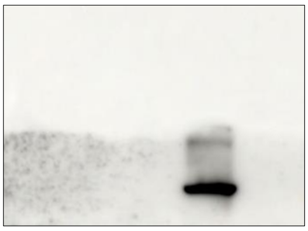

Supp. Figure S1D-IP-Moesin, IB-FBXW2

Supp. Figure S1D-IP-Moesin, IB-Moesin

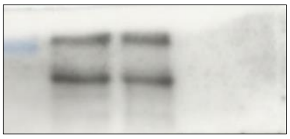

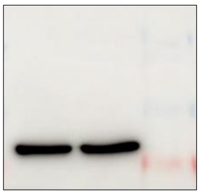

Supp. Figure S1D-Input, IB-FBXW2

Supp. Figure S1D-Input, IB-Moesin

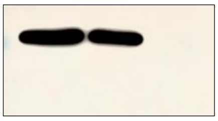

Supp. Figure S1D-Input, IB-Tubulin

Supplementary Figure S2

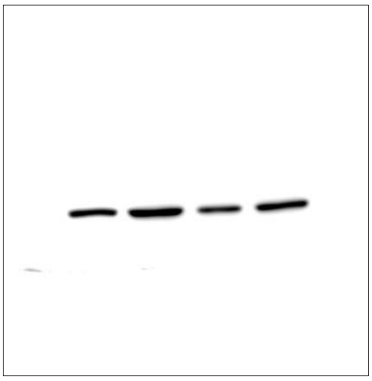

Supp. Figure S2A, IB-Moesin

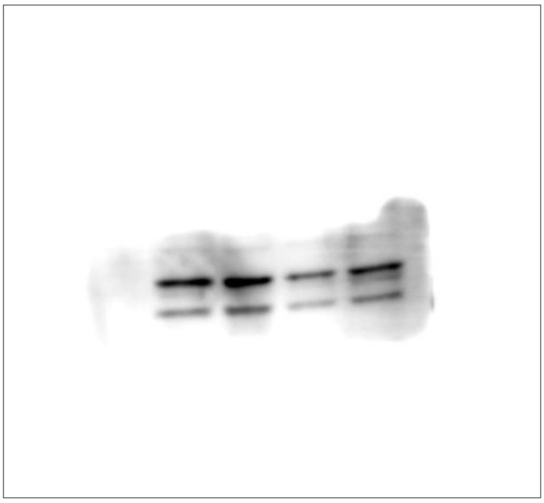

Supp. Figure S2A, IB-SKP2

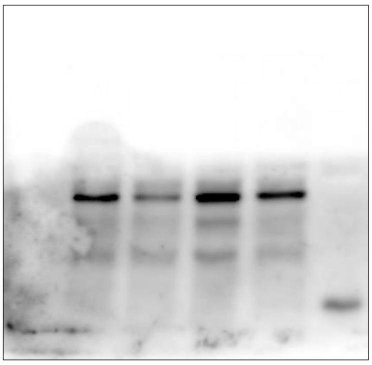

Supp. Figure S2A, IB-FBXW2

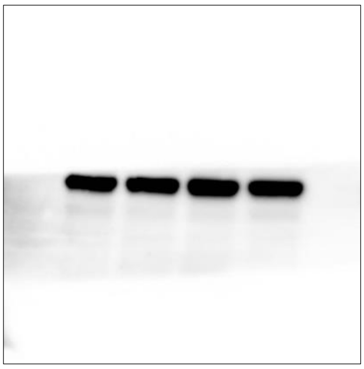

Supp. Figure S2A, IB-Tubulin

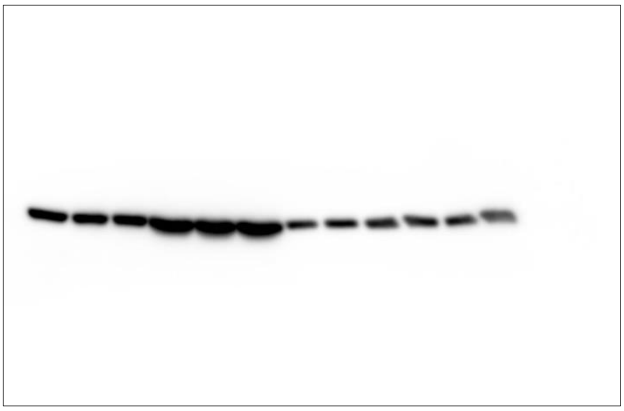

Supp. Figure S2K, IB-Moesin

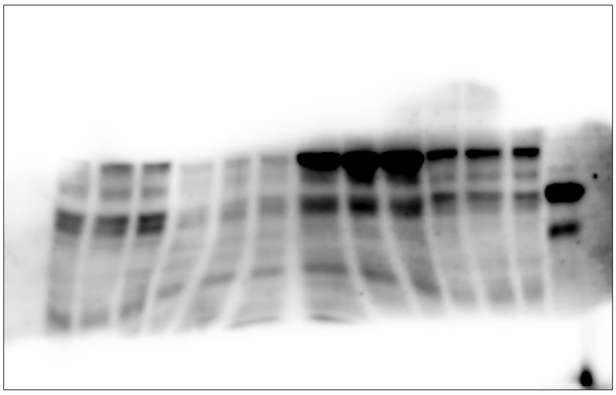

Supp. Figure S2K, IB-FBXW2

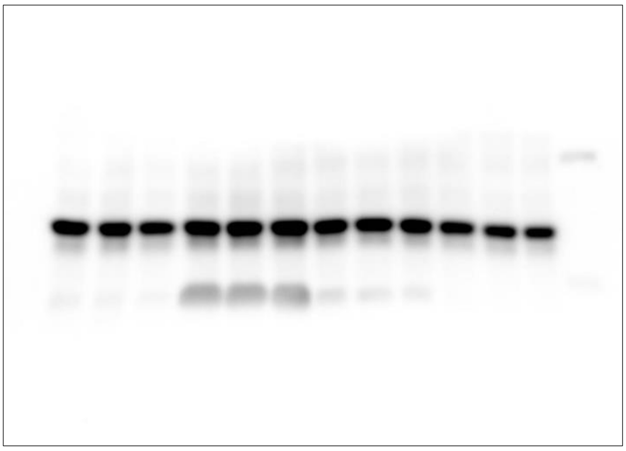

Supp. Figure S2K, IB-GAPDH

Supplementary Figure S3

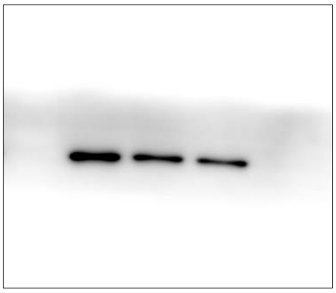

Supp. Figure S3A, IB-Moesin

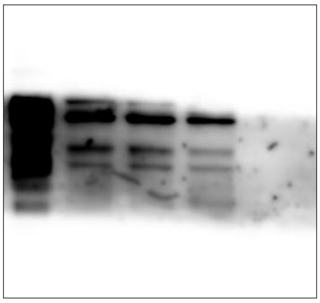

Supp. Figure S3A, IB-SKP2

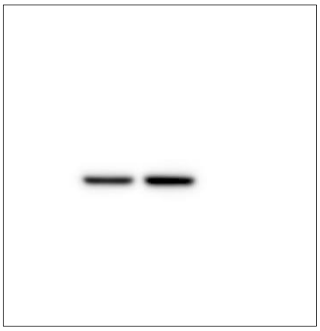

Supp. Figure S3A, IB-myc

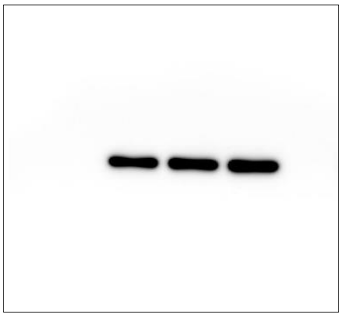

Supp. Figure S3A, IB-Tubulin

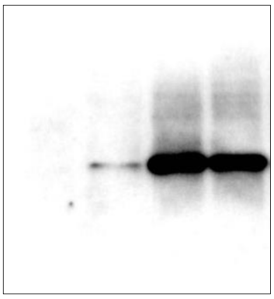

Supp. Figure S3C, IB-Moesin

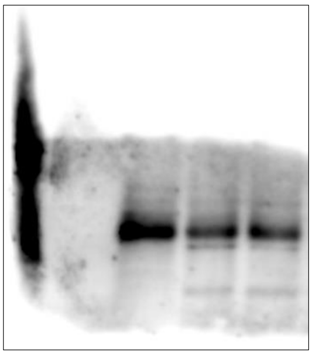

Supp. Figure S3C, IB-FBXW2

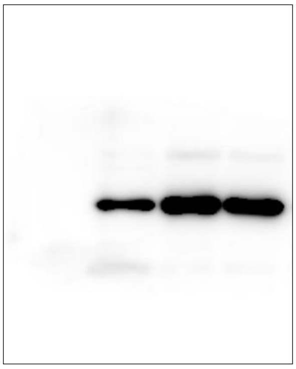

Supp. Figure S3C, IB-β-Actin

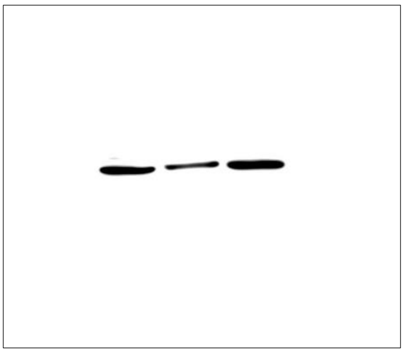

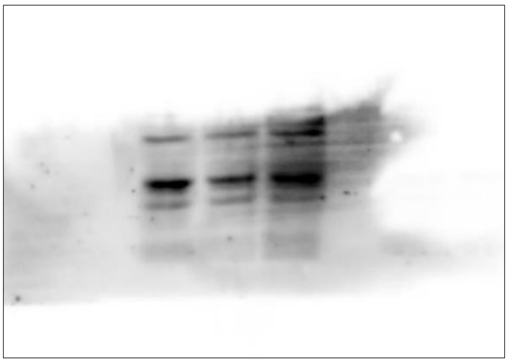

Supp. Figure S3D, IB-Moesin

Supp. Figure S3D, IB-SKP2

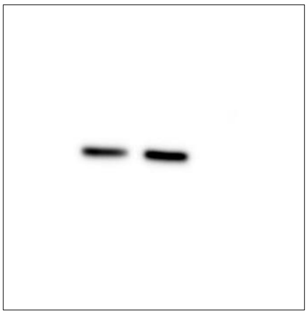

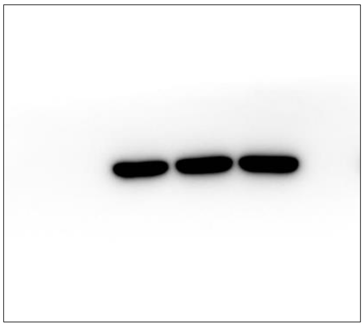

Supp. Figure S3D, IB-myc

Supp. Figure S3D, IB-Tubulin

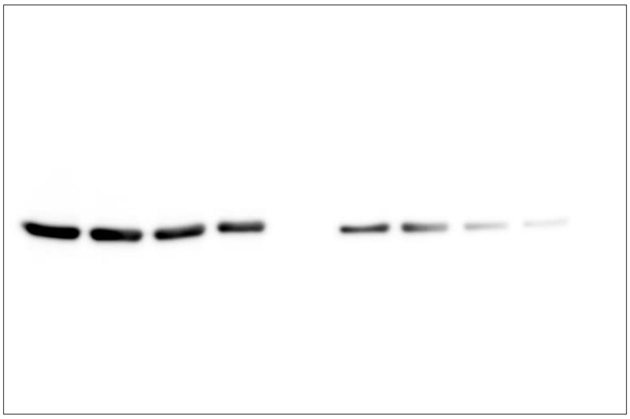

Supp. Figure S3E, IB-Moesin

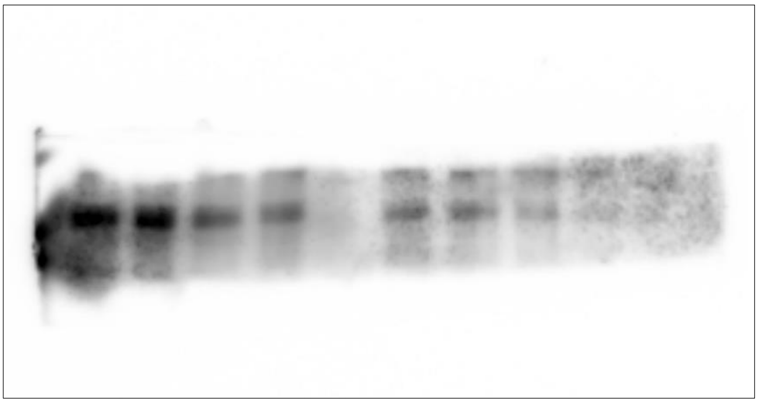

Supp. Figure S3E, IB-SKP2

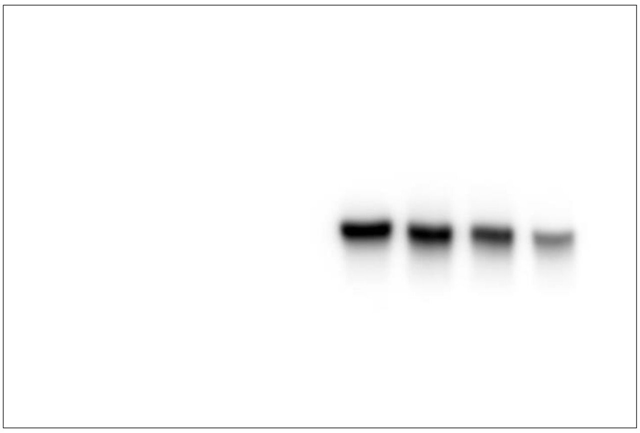

Supp. Figure S3E, IB-myc

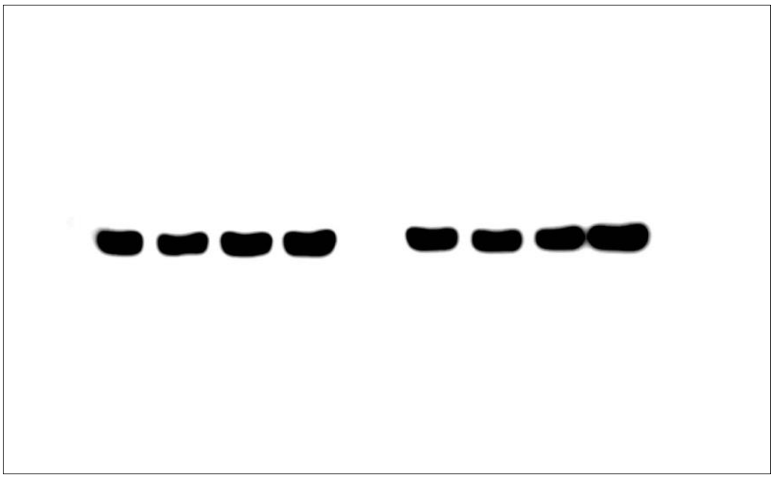

Supp. Figure S3E, IB-Tubulin

Supplementary Figure S4

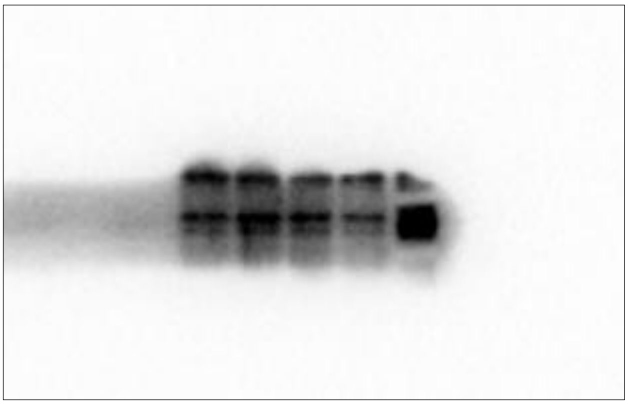

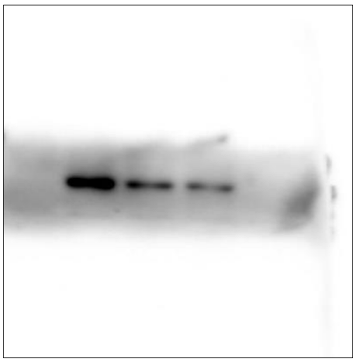

Supp. Figure S4A, IB-SKP2

Supp. Figure S4A, IB-His

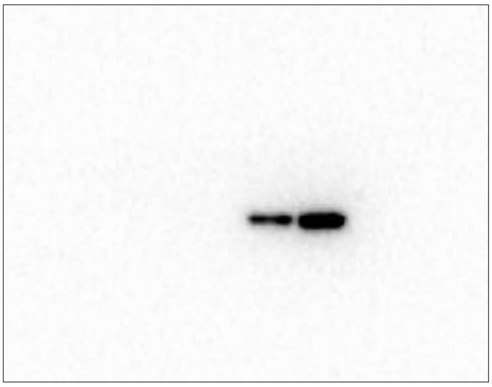

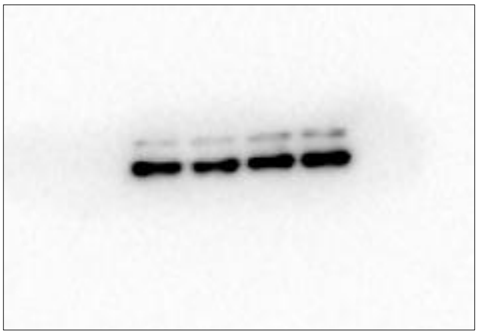

Supp. Figure S4A, IB-FLAG

Supp. Figure S4A, IB-Tubulin

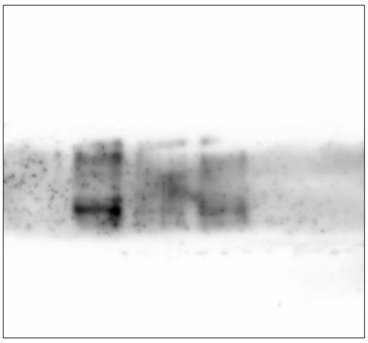
Supp. Figure S4B, IB-SKP2

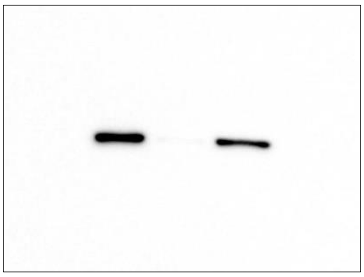
Supp. Fig. S4B, IB-Moesin

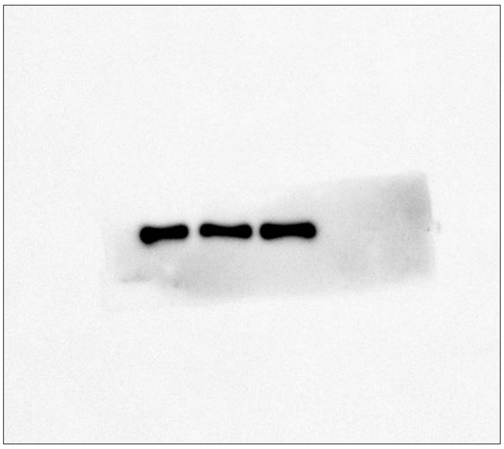
Supp. Fig. S4B, IB-Tubulin

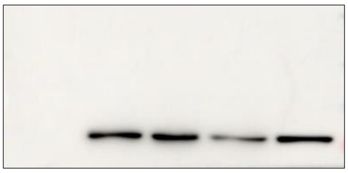
Supp. Figure S4D, IB-Moesin

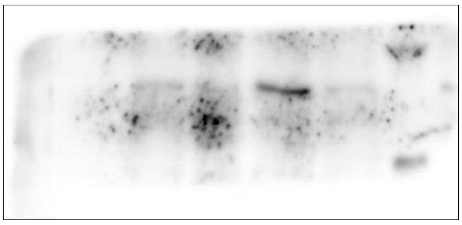
Supp. Figure S4D, IB-FBXW2

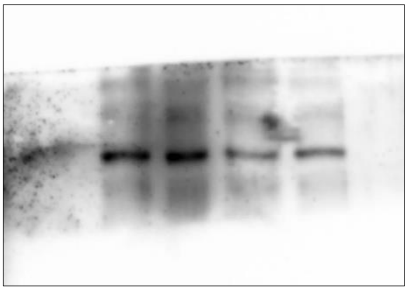
Supp. Figure S4D, IB-SKP2

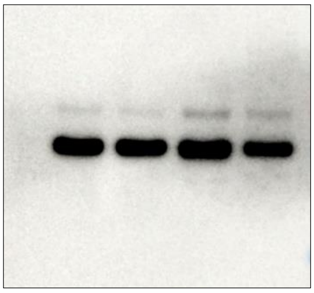
Supp. Figure S4D, IB-Tubulin

Supplementary Figure S5

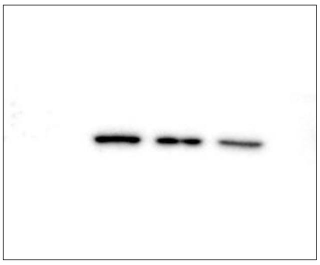

Supp. Figure S5A, IB-Moesin

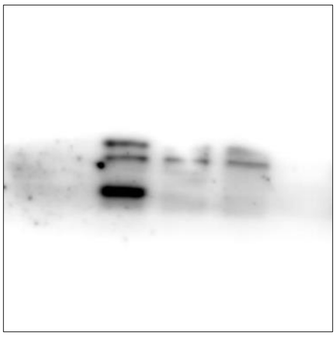

Supp. Figure S5A, IB-pAKT (T308)

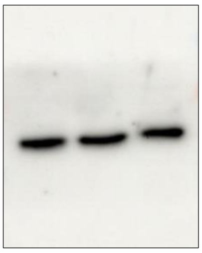

Supp. Figure S5A, IB-tAKT

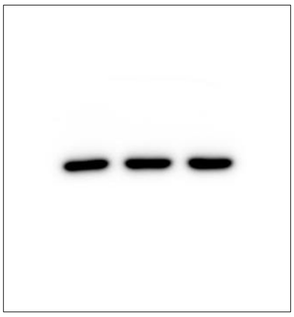

Supp. Figure S5A, IB-GAPDH

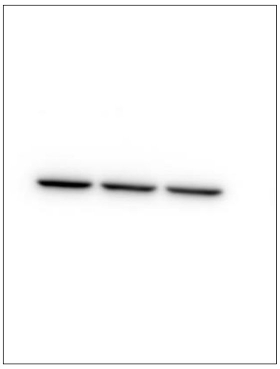

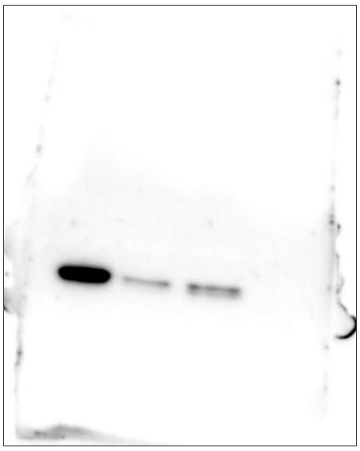

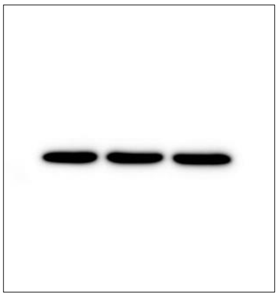

Supp. Figure S5B, IB-Moesin

Supp. Figure S5B, IB-AKT

Supp. Figure S5B, IB-GAPDH

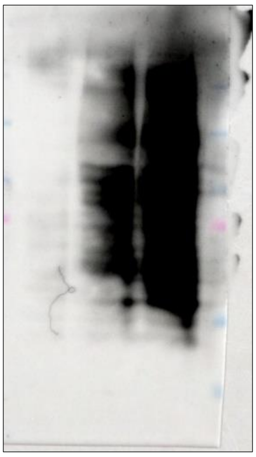

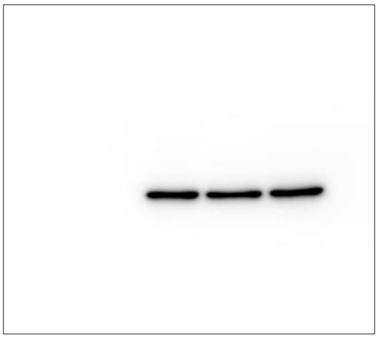

Supp. Figure S5C-PD-Ni-NTA, IB-Moesin

Supp. Figure S5C-Input, IB-Moesin

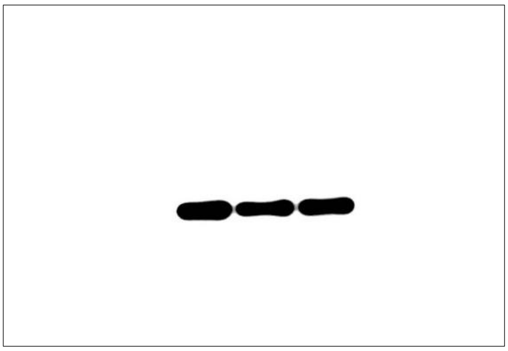

Supp. Figure S5C-Input, IB-Tubulin

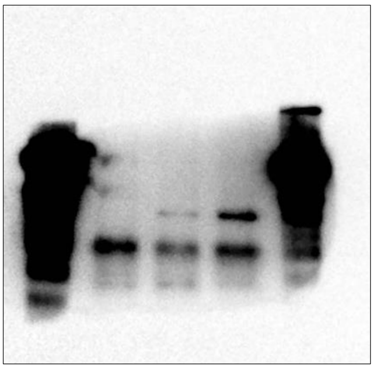

Supp. Fig. S5D-IP-FLAG, IB-His

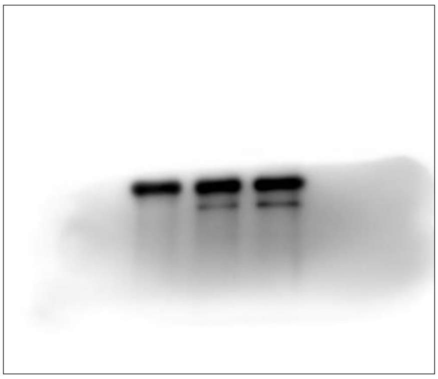

Supp. Fig. S5D-IP-FLAG, IB-FLAG

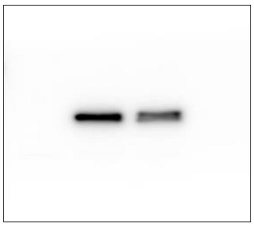

Supp. Fig. S5D-Input, IB-His

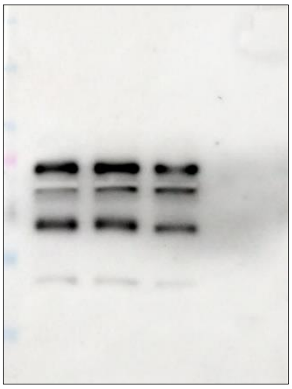

Supp. Figure S5D-Input, IB-pAKT (T308)

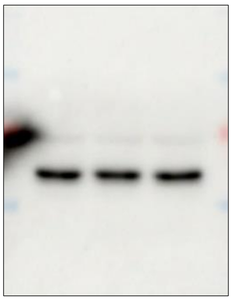

Supp. Figure S5D-Input, IB-AKT

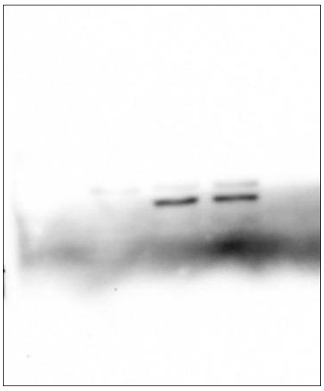

Supp. Figure S5D-Input, IB-FLAG

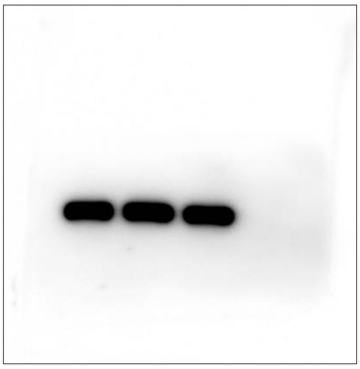

Supp. Figure S5D-Input, IB-Tubulin

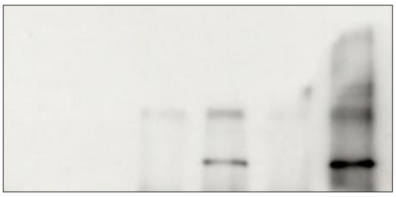

Supp. Fig. S5E-IP-myc, IB-Moesin

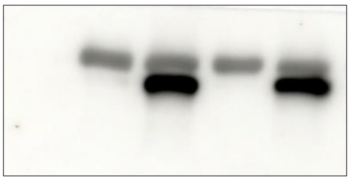

Supp. Fig. S5E-IP-myc, IB-myc

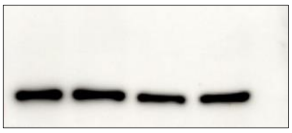

Supp. Fig. S5E-Input, IB-Moesin

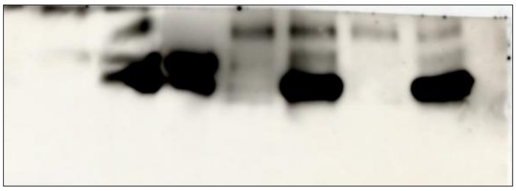

Supp. Fig. S5E-Input, IB-AKT

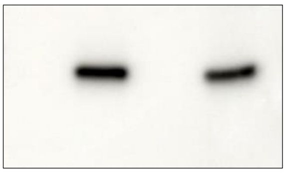

Supp. Fig. S5E-Input, IB-myc

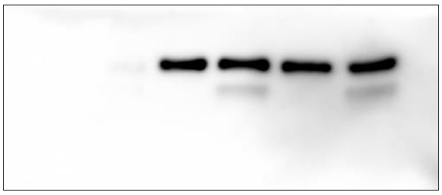

Supp. Fig. S5E-Input, IB-Tubulin

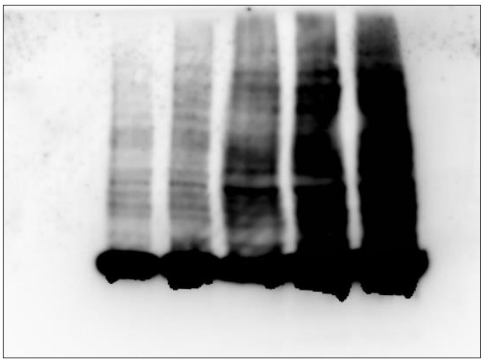

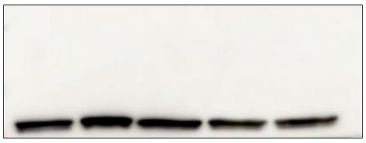

Supp. Fig. S5F-PD-Ni-NTA, IB-Moesin

Supp. Fig. S5F-Input, IB-Moesin

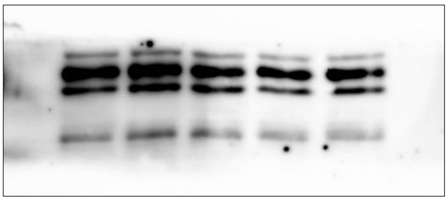

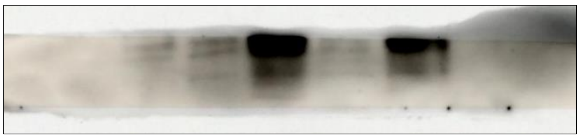

Supp. Fig. S5F-Input, IB-AKT

Supp. Fig. S5F-Input, IB-myc

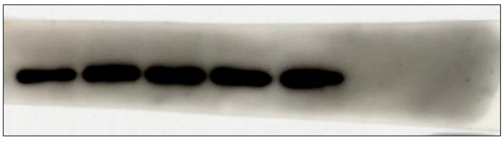

Supp. Fig. S5F-Input, IB-Tubulin
